# Supplementary material for: Astronomically calibrating early Ediacaran evolution
Source: Nat Commun. 2025 Mar 28;16:3049. doi: 10.1038/s41467-025-57201-1 (PMC11953472; doi:10.1038/s41467-025-57201-1)
Supplement: Supplementary file 1 — Supplementary Information [file 41467_2025_57201_MOESM1_ESM.docx]

**Supplementary Information for**

**Astronomically calibrating early Ediacaran evolution**

Tan Zhang^1,2,3^, Chao Ma^1,2,⁎^, Yifan Li^3*^, Chao Li^1^, Anne-Christine Da Silva^4^, Tailiang Fan^3^, Qi Gao^5^, Mingzhi Kuang^6^, Wangwei Liu^7^, Mingsong Li^8^, Mingcai Hou^1,2^

^1^State Key Laboratory of Oil and Gas Reservoir Geology and Exploitation & Institute of Sedimentary Geology, Chengdu University of Technology, Chengdu 610059, China.

^2^Key Laboratory of Deep-time Geography and Environment Reconstruction and Applications of Ministry of Natural Resources, Chengdu University of Technology, Chengdu 610059, China.

^3^School of Energy Resources, China University of Geosciences (Beijing), Beijing 100083, China.

^4^Sedimentary Petrology Laboratory, University of Liege, Sart Tilman B20, Allée du Six Août 12, Liège 4000, Belgium.

^5^College of Geography and Planning, Chengdu University of Technology, Chengdu 610059, China.

^6^College of Energy, Chengdu University of Technology, Chengdu 610059, China.

^7^Wuxi Research Institute of Petroleum Geology, Research Institute of Petroleum Exploration and Production, SINOPEC, Wuxi 214151, China.

^8^Key Laboratory of Orogenic Belts and Crustal Evolution, MOE, School of Earth and Space Sciences, Peking University, Beijing 100871, China.

*Corresponding authors. Email: [machao@cdut.edu.cn](mailto:machao@cdut.edu.cn); [yifangeosci@gmail.com](mailto:yifangeosci@gmail.com).

**The Supplementary Information includes:**

Supplementary Notes 1-6

Supplementary Text 1 Settings and outputs of the TimeOpt analysis

Supplementary Figures 1-31

Supplementary References

**Other Supplementary Material for this manuscript includes the following:**

**Supplementary Data 1** All isotopic results and geochemical data in this study

**Supplementary Data 2** Magnetic susceptibility data, age models, floating astronomical time scale and sedimentation rate on 405 kyr scale

**Supplementary Data 3** The predicted estimates of long eccentricity cycles from the astronomical solution ZB23-N64 at 610 Ma

**Supplementary Data 4** A compilation of the Ediacaran strontium isotope ratios (⁸⁷Sr/⁸⁶Sr) and their age model from South China, Australia, and Namibia

**Supplementary Data 5** A compilation of the Ediacaran δ^13^C_carb_ data and their age model from Australia, South China, Laurientia, Mongolia, Namibia, Oman, and Siberia

**Supplementary Note 1. Evaluation of diagenetic and chemostratigraphic correlation for δ^13^C_carb_ and ^87^Sr/^86^Sr data**

All isotopic results and geochemical data are presented in Fig. 2, Supplementary Figs. 2 and 6 and in Supplementary Data 1. A brief description of diagenetic evaluation for these data is provided below.

**1.1 Carbonate carbon isotopes**

*1.1.1 Evaluation of diagenetic influences on δ^13^C_carb_*

Diagenetic alteration typically results in a positive covariation between δ^13^C_carb_ and δ^18^O_carb_, along with very low δ^18^O values (often < -10‰) due to the simultaneous depletion of ^13^C and ^18^O^1^. However, samples from the EYC2, WD1, and ZK68 drillcores do not exhibit this pattern. In the EYC2 drillcore, 97% of samples (66/68 samples) have δ^18^O > -10‰, with comparable results in WD1 (97%, 82/85 samples) and ZK68 (83.3%, 55/60 samples) (Supplementary Data 1). These results, combined with the absence of positive covariation between δ^13^C_carb_ and δ^18^O_carb_ as shown in Supplementary Figs. 3-5, suggest minimal diagenetic alteration in the majority of samples across all three drillcores. The consistent pattern across different drillcores indicates that the observed isotopic signatures are likely primary rather than the result of post-depositional alteration.

In reducing diagenetic fluids, marine carbonates tend to expel Sr and incorporate Mn, making Mn/Sr ratio an indicator of diagenetic alteration^2^. Samples with Mn/Sr <10 typically retain primary δ^13^C_carb_ signatures^2–4^. Our drillcore samples consistently show low Mn/Sr ratios: all samples from EYC2 and WD1 are <3.53 (99.3% samples <3), while all ZK68 samples are <3 (Supplementary Data 1). Furthermore, petrographic observations revealed no evidence of recrystallization or mineralogical variations. This combined geochemical and petrographic evidence strongly supports the preservation of near-primary seawater δ^13^C_carb_ signatures in our samples.

*1.1.2 Stratigraphic correlation of δ^13^C_carb_*

The Doushantuo Formation of the Yangtze Platform has been the subject of extensive carbon isotope studies, revealing several significant δ^13^C_carb_ excursions. Previous research has identified three major negative excursions (EN1, EN2, and EN3) that correspond to CANCE, BAICE, and DOUCE respectively (i.e., CAp carbonate Negative Carbon isotope Excursion; CANCE, Weng'An Negative Carbon isotope Excursion, WANCE; BAIguoyuan Negative Carbon isotope Excursion, BAICE; and DOUshantuo Negative Carbon isotope Excursion, DOUCE)), along with an additional excursion (WANCE)^5–13^. The EN3 event, also widely referred to as the Shuram excursion, represents the largest negative δ^13^C excursion in Earth's history and is globally recognized in carbonate successions^14^.

Our study of the lower-middle Doushantuo Formation across different depositional settings reveals a consistent pattern: three negative δ^13^C_carb_ excursions (EN1, WANCE, and EN2) in the intrashelf basin (WD1 and EYC2 drillcores) and two (EN1 and EN2) in the lower slope (ZK68 drillcore). These profiles correlate well with other sections across the Yangtze Platform based on lithostratigraphy (Supplementary Fig. 2).

The global significance of these excursions varies. EN1 and EN3 have been identified worldwide in coeval strata^6,10,12,14–19^. EN2 appears to be at least a regional event, observed in South China, Northern Namibia, and Newfoundland^6,20,21^. The WANCE event, primarily documented in intrashelf basin settings on the Yangtze Platform, may be facies-dependent^22–25^. Additionally, a positive δ^13^C_carb_ excursion (EP1) in the middle parts of Member II has been reported across various settings of the Yangtze Platform, including the inner shelf, intrashelf basin, and upper slope^10,13^. This integrated approach to δ^13^C_carb_ stratigraphy across different depositional settings provides a comprehensive view of carbon cycle dynamics during the Ediacaran period, highlighting both global events and potential local variations.

**1.2 Strontium isotope**

*1.2.1* *Evaluating the influence of post-depositional alteration and detrital components on ^87^Sr/^86^Sr*

Marine carbonate Sr isotopic compositions are likely modified by diagenesis and detrital components^25^. Diagenetic processes involving ^87^Sr-rich interstitial fluids can increase ^87^Sr/^86^Sr values^26,27^, while interaction with less radiogenic fluids from volcanic or hydrothermal sources can decrease them while interaction with less radiogenic fluids from volcanic or hydrothermal sources can decrease them^28–30^. To assess the preservation of primary seawater ^87^Sr/^86^Sr ratios in the Doushantuo carbonate, we compared measured ^87^Sr/^86^Sr ratios with conventional diagenetic and detrital alteration indicators (Mn/Sr ratios, δ^18^O_carb_, Mn, Sr, Rb, Al concentrations) (Supplementary Figs. 3-5 and Supplementary Data 1). Our samples exhibit favorable characteristics: high Sr (211.75-1994 ppm, avg. 792.32 ppm), low Mn (77.46-414.01 ppm, avg. 182.08 ppm), low Rb (0.83-22.1 ppm, avg. 8.83 ppm), low Mn/Sr (0.04-1.37, avg. 0.26), and low Rb/Sr ratios (0.0007-0.03, avg. 0.0093) (Supplementary Figs. 3-5 and Supplementary Data 1). The lack of significant covariation between ^87^Sr/^86^Sr ratios and these indicators, including δ^18^O_carb_, suggests minimal diagenetic overprinting (Supplementary Figs. 3-5 and Supplementary Data 1).

To assess detrital influence, we examined correlations between ^87^Sr/^86^Sr ratios and Al content, Rb content, and Rb/Sr ratios^9,25^. The absence of such correlations indicates minimal influence from detrital components in our samples (Supplementary Figs. 3-5). These multiple lines of evidence suggest that the measured ^87^Sr/^86^Sr ratios in the WD1, EYC2, and ZK68 drillcores were not significantly influenced by diagenetic alteration or siliciclastic contamination, likely preserving primary seawater signatures.

*1.2.2 Stratigraphic correlation of ^87^Sr/^86^Sr*

The ^87^Sr/^86^Sr ratios in the EYC2 and WD1 drillcores range from 0.707908 to 0.71259 (with an average of 0.70859), with the highest values in Member I and at the base of Member II. These ratios show a gradual increase throughout the succession, featuring three significant positive excursions (P1, P1-1, and P2; Supplementary Fig. 6). In the ZK68 drillcore, nine samples from the top of Member II and Member III yield ^87^Sr/^86^Sr ratios between 0.708254 and 0.708783 (with an average of 0.708449), with one significant positive excursion (P2) near the Member II-III boundary (Supplementary Fig. 6). These positive excursions in EYC2, WD1, and ZK68 drillcores align with those reported from other Yangtze Platform sections in South China^9,19,23,25,31–33^. Notably, these ^87^Sr/^86^Sr excursions coincide with four negative δ^13^C_carb_ excursions in the Doushantuo Formation: in Member I, middle Member II, near the Member II-III boundary, and from upper Member III to Member IV (Supplementary Fig. 6).

*1.2.3 ^87^Sr/^86^Sr ratio as a paleoclimate proxy*

Seawater strontium isotopic composition is globally homogeneous due to strontium's long residence time (~10^6^ years) compared to ocean circulation time (~10^3^ years)^34–36^. The secular trend of seawater Sr isotopes is primarily controlled by the balance between radiogenic ^87^Sr input from continental weathering and less radiogenic ^87^Sr from mantle sources at mid-ocean ridges^35,37–41^. Elevated ^87^Sr/^86^Sr events in the Ediacaran Period could be attributed to: 1) increased ^87^Sr/^86^Sr ratios in weathered rocks, 2) reduced seafloor spreading rates or mantle strontium input, or 3) increased continental weathering rates^39^. Considering the similarity of continental runoff ^87^Sr/^86^Sr ratios between the Neoproterozoic and present^42^ and the unlikelihood of sustained decline in seafloor spreading rates during rapid continental reorganization^43^, the elevated Ediacaran ^87^Sr/^86^Sr ratios are best explained by an overall increase in continental weathering rates^39^.

**Supplementary Note 2. Early Ediacaran astronomical parameters**

Accurate determination of astronomical parameters is crucial for precise time-calibration of Milankovitch-driven sedimentary cycles. Earth's orbital parameters beyond 50 Ma are highly uncertain due to the Solar system's chaotic behavior^44,45^.

The prediction of obliquity and precession periods accumulates bias further back in time due to the influence of Earth’s past tidal dissipation. To address this issue, previous studies have employed two primary assumptions: a constant lunar recession rate^46,47^ or a constant tidal time lag^48^ for estimating obliquity and precession frequencies. However, these approaches inherently lead to inaccuracies. Assuming a constant lunar recession rate tends to overestimate the Earth–Moon separation, whereas the assumption of a constant tidal time lag results in an underestimation^49^. This study utilizes the latest orbital solutions ZB23-N64 developed by Zeebe and Lantink^50,51^, which are proposed as potential templates for investigating orbital characteristics in deep time beyond −300 Myr (see www.ncdc.noaa.gov/paleo/study/39199)^52^. For approximately 610 Ma (the mean age of the studied Doushantuo Formation), the model predicts a primary obliquity cycle of 30.65 kyr and periods of ~18-15 kyr (averaging ~16.5 kyr) for the precession cycles, of 94.5-132.3 kyr for short eccentricity (averaging ~112.8 kyr) and of 403-408 kyr for long eccentricity (averaging ~405.28 kyr)^50–52^. These theoretically predicted astronomical parameters (~405 kyr, ~112.8 kyr, ~30.65 kyr, and ~16.5 kyr) will be used to identify and interpret Milankovitch-influenced sedimentary cycles in the stratigraphic record.

**Supplementary Note 3. Cyclostratigraphic analysis**

**3.1 Power spectra analyses in depth domain**

The multi-taper method (MTM) power spectrum of the untuned MS series through the entire stratigraphic interval in the WD1 drillcore shows dominant wavelengths of 13.42, 4.97-1.34, 0.72-0.48, 0.19-0.15, and 0.12-0.084 m, with above the 95% confidence level (Supplementary Fig. 7a). In the ZK68 drillcore, the MTM power spectrum of untuned MS series across the entire stratigraphic interval reveals significant wavelengths at ~21.7 m, 5.62-0.50 m, 0.32-0.20 m, 0.08-0.065 m and 0.05-0.041 m, with above the 95% confidence level (Supplementary Fig. 8a). When the sedimentation rate undergoes significant changes, an astronomical orbital period can be recorded at multiple frequencies, making it challenging to accurately attribute these sedimentary cycles to specific astronomical orbital parameters^53^. Thus, we track the frequency changes throughout the stratigraphic interval using the evolution Fast Fourier Transform (i.e., eFFT).

The eFFT of the untuned MS series reveals frequency changes at depths of ~2132 m, ~2110 m, ~2088 m, and ~2067.26 m in the WD1 drillcore (Supplementary Fig. 7b), and at approximate depths of ~1471.99 m, ~1458.38 m, ~1445.72 m, ~1430 m, ~1413.5 m, and ~1399.21 m, in the ZK68 drillcore (Supplementary Fig. 8b). These frequency changes, combined with observed lithofacies transitions and lithostratigraphic boundaries, provide a basis for dividing the untuned MS series into multiple subsets across the entire stratigraphic interval for both the WD1 and ZK68 drillcores. In the WD1 drillcore, we established five subsets: D2-1 (2155.4-2132 m), D2-2 (2132-2110 m), D2-3 (2110-2088 m), D2-4 (2088-2067.26 m), and D3 (2067.2-2040 m) (Supplementary Fig. 7b). The ZK68 drillcore was divided into seven Subsets: D1 (1479.50-1471.99 m), D2-1 (1471.99-1458.38 m), D2-2 (1458.38-1445.72 m), D2-3 (1445.72-1430.00 m), D2-4 (1430-1413.5 m), D2-5 (1413.5-1399.44 m), and D3 (1399.44-1384 m) (Supplementary Fig. 8b).

*3.1.1 WD1 drillcore*

For Subset D2-1 (2155.4-2132 m), the MTM power spectrum exhibits obvious peaks at 6.16, 1.81-1.23 m, 0.33-0.26 m, and 0.095-0.077 m (Supplementary Fig. 7c), fitting with the theoretical periodic ratios of long eccentricity, short eccentricity, and obliquity (i.e., ~405 kyr, ~112.8 kyr, ~30.6 kyr, and ~16.5 kyr) during the Ediacaran Period (See Supplementary Note 2). Based on estimated average sedimentation rates of 0.37 cm/kyr, the sedimentary cycles of 1.81-1.23 m, 0.33-0.26 m, and 0.095-0.077 m likely represent long eccentricity, short eccentricity, and obliquity cycles, respectively (Supplementary Fig. 7c).

For Subset D2-2 (2132-2110 m), the MTM power spectrum of untuned MS series reveals significant wavelengths at ~1.87 m, 0.61-0.40 m, 0.17-0.13 m and 0.09-0.07 m (Supplementary Fig. 7c), matching well with the theoretical ratios of astronomical parameters (i.e., ~405 kyr, ~112.8 kyr, ~30.6 kyr, and ~16.5 kyr; see Supplementary Note 2). Based on the average sedimentation rates of 0.35 cm/kyr, the observed cycles likely correspond to long eccentricity, short eccentricity, obliquity and precession cycles, respectively (Supplementary Fig. 7c).

For Subset D2-3 (2110-2088 m), the MTM power spectrum shows significant wavelength peaks of 6.11 m, 0.75-0.43 m, 0.19-0.16 m, and 0.11-0.09 m (Supplementary Fig. 7c), which are approximately comparable with the theoretical ratios of astronomical parameters (i.e., ~405 kyr, ~112.8 kyr, ~30.6 kyr, and ~16.5 kyr; see Supplementary Note 2 for details). Based on the average sedimentation rates of 0.49 cm/kyr, these cycles likely are interpreted as long eccentricity, short eccentricity, obliquity and precession cycles, respectively (Supplementary Fig. 7c).

For Subset D2-4 (2088-2067.26 m), the MTM power spectrum shows dominant wavelengths of 1.34, 0.33 m, and 0.10-0.089 m (Supplementary Fig. 7c), matching approximately with the theoretical ratios of astronomical parameters (i.e., ~405 kyr, ~112.8 kyr, and ~30.6 kyr; see Supplementary Note 2 for details). Thus, according to the average sedimentation rates of 0.37 cm/kyr, these cycles likely represent long eccentricity, short eccentricity, and obliquity cycles, respectively (Supplementary Fig. 7c).

For Subset D3 (2067.26-2040 m) in the WD1 drillcore, the MTM power spectrum shows dominant wavelengths of 3.60 m, 1.02-0.75 m, 0.28 m, and 0.17-0.12 m (Supplementary Fig. 7c), comparable well with the theoretical ratios of astronomical parameters (i.e., ~405 kyr, ~112.8 kyr, ~30.6 kyr, and ~16.5 kyr). Thus, according to the average sedimentation rates of 0.65 cm/kyr, we interpret these cycles as long eccentricity, short eccentricity, obliquity and precession cycles, respectively (Supplementary Fig. 7c).

These findings are consistent with the wavelengths identified from the same interval through Lomb-Scargle spectrum analysis using smoothed window averages^54^ (Supplementary Fig. 7d). Thus, the sedimentary cycles of 1.81-1.23 m, 1.87 m, 6.11 m, 1.34 m, and 3.60 m in the five Subsets of the WD1 drillcore are likely indicative of variations in the wavelength of the 405-kyr long eccentricity cycles. These variations, attributed to fluctuating sedimentation rates, have been effectively isolated using Gaussian bandpass filtering, revealing approximately 71.2 cycles throughout the entire MS series.

*3.1.2 ZK68 drillcore*

For Subset D1 (1479.50-1471.99 m), the MTM power spectrum displays significant peaks at wavelengths of 6.26-2.09 m, 0.46 m, 0.19 m, and 0.12-0.086 m (Supplementary Fig. 8c), matching well with the theoretical ratios of astronomical parameters (i.e., ~405 kyr, ~112.8 kyr, ~30.6 kyr, and ~16.5 kyr). Based on the average sedimentation rate of 0.66 cm/kyr, the observed cycles likely represent long eccentricity, short eccentricity, obliquity and precession cycles, respectively (Supplementary Fig. 8c).

For Subset D2-1 (1471.99-1458.38 m), the MTM power spectrum exhibits peaks at 4.55 m, 0.57-0.44 m, and 0.137-0.096 m (Supplementary Fig. 8c). The ratios between the 0.57–0.44 m and 0.137–0.096 m cycles closely align with theoretical ratios of astronomical parameters (i.e., ~405 kyr, and ~112.8 kyr). According to the estimated average sedimentation rates of 0.16 cm/kyr, we interpret these cycles as long eccentricity, and short eccentricity cycles, respectively (Supplementary Fig. 8c).

For Subset D2-2 (1458.38-1445.72 m), the MTM power spectrum shows obvious cycles with wavelengths at 4.22 m, ~0.93 m, 0.26-0.19 m, and 0.075-0.066 m (Supplementary Fig. 8c). The ratios between the 0.93 m, 0.26–0.19 m and 0.075-0.066 m cycles closely align with theoretical ratios of astronomical parameters (i.e., ~405 kyr, ~112.8 kyr, and ~30.6 kyr). Based on the estimated average sedimentation rates of 0.18 cm/kyr, these cycles represent long eccentricity, short eccentricity, and obliquity cycles, respectively (Supplementary Fig. 8c).

For Subset D2-3 (1445.72-1430.00 m), the MTM power spectrum displays significant peaks at wavelengths of 2.81 m, 0.44 m, and 0.15-0.09 m (Supplementary Fig. 8c). The ratios between the 0.44 m and 0.15–0.09 m cycles closely align with theoretical ratios of astronomical parameters (i.e., ~405 kyr and ~112.8 kyr). Based on the estimated average sedimentation rates of 0.10 cm/kyr, these cycles are likely interpreted as long eccentricity, and short eccentricity cycles, respectively (Supplementary Fig. 8c).

For Subset D2-4 (1430-1413.5 m), the MTM power spectrum exhibits peaks at 3.93 m, 0.67 m, 0.22-0.15 m and ~0.05-0.04 m (Supplementary Fig. 8c). The ratios between the 0.67 m, 0.22-0.15 m and ~0.05-0.04 m cycles closely align with theoretical ratios of astronomical parameters (i.e., ~405 kyr, ~112.8 kyr, and ~30.6 kyr). Thus, according to the estimated average sedimentation rates of 0.14 cm/kyr, we interpret these cycles as long eccentricity, short eccentricity and obliquity cycles, respectively (Supplementary Fig. 8c).

For Subset D2-5 (1413.5-1399.44 m), the MTM power spectrum exhibits peaks at 2.71 m, 0.62-0.48 m, 0.21-0.16 m, and 0.51-0.45 m (Supplementary Fig. 8c). The ratios between the 0.62-0.48 m, 0.21-0.16 m, and 0.51-0.45 m cycles closely align with theoretical ratios of astronomical parameters (i.e., ~405 kyr, ~112.8 kyr, and ~30.6 kyr). Consequently, based on the estimated average sedimentation rates of 0.15 cm/kyr, these cycles likely represent long eccentricity, short eccentricity and obliquity cycles, respectively (Supplementary Fig. 8c).

For Subset D3 (1399.44-1384 m), the MTM power spectrum shows significant wavelength peaks of 2.2 m, 0.76-0.48 m, 0.16 m, and 0.11-0.087 m (Supplementary Fig. 8c), which are approximately comparable with the theoretical ratios of astronomical parameters (i.e., ~405 kyr, ~112.8 kyr, ~30.6 kyr, and ~16.5 kyr; see Supplementary Note 2). Based on the estimated average sedimentation rates of 0.53 cm/kyr, these cycles likely are interpreted as long eccentricity, short eccentricity, obliquity and precession cycles, respectively (Supplementary Fig. 8c).

These findings are consistent with the wavelengths identified from the same interval through Lomb-Scargle spectrum analysis using smoothed window averages^54^. Thus, the observed wavelengths of 6.26-2.09 m, 0.57-0.44 m, ~0.93 m, 0.44 m, 0.67 m, 0.62-0.48 m, and 2.2 m in the seven Subsets are interpreted as representing variations in the 405-kyr long eccentricity cycle. These variations were then filtered, resulting in 142.2 cycles identified within the entire MS series.

*3.1.3 EYC2 drillcore*

The MTM power spectrum of the entire uncalibrated MS in the EYC2 drillcore displays significant peaks at wavelengths of 3.52 m, 1.82-1.25 m, 0.64-0.42 m, 0.16-0.13 m and 0.09-0.07 m (Supplementary Fig. 9a). These findings are consistent with the wavelengths identified from the same interval through Lomb-Scargle spectrum analysis using smoothed window averages (Supplementary Fig. 9b). The ratios between the observed cycle fit well with the theoretical ratios of astronomical parameters (i.e., ~405 kyr, ~112.8 kyr, ~30.6 kyr, and ~16.5 kyr). Based on the estimated average sedimentation rates of 0.35 cm/kyr, the observed cycles likely represent long eccentricity, short eccentricity, obliquity and precession cycles, respectively (Supplementary Fig. 9a, b). By filtering out the 1.82-1.25 m cycles, 22.5 cycles are identified within the entire MS series.

**3.2 Orbital tuning**

Sedimentary cycles associated with the ~405-kyr long eccentricity cycle, identified in the magnetic susceptibility (MS) series from the EYC2, WD1, and ZK68 drillcores, are filtered and tuned to the time domain. These processes enabled the construction of floating astronomical time scales spanning ca. 9.12 Myr (EYC2), 28.83 Myr (WD1), and 57.59 Myr (ZK68), respectively (Fig. 3 and Supplementary Data 2).

Sedimentation rate curves across the studied interval in the EYC2, WD1, and ZK68 drillcores were reconstructed at the ~405-kyr scale by identifying the maxima of each 405-kyr cycle (Supplementary Data 2; Supplementary Fig. 29). Our analysis reveals systematic variations in sedimentation rates derived from the maxima of the 405-kyr eccentricity cycles, which integrate paleogeographic setting, lithological variation, and the sequence stratigraphic framework. The average sedimentation rates show a clear basinward decrease, declining from WD1 (shallow intra-shelf basin, ~0.4 cm/kyr) through EYC2 (deep intra-shelf basin, ~0.36 cm/kyr) to ZK68 (lower slope setting, ~0.17 cm/kyr) (Supplementary Data 2; Supplementary Fig. 29). These trends are consistent with depositional gradients and the paleogeographic positions of the drill sites. Furthermore, sedimentation rates exhibit an inverse correlation with sea-level reconstructions based on sequence stratigraphy, aligning with sedimentary dynamics (Supplementary Fig. 29). Within depositional sequences, sedimentation rates demonstrate systematic variations tied to the characteristics of different systems tracts. Sedimentation rates reach their peak during the early transgressive systems tract (TST), where carbonate-dominated units are prevalent. This reflects rapid sea-level rise, increased accommodation space, and high carbonate production rates (Supplementary Fig. 29). In contrast, sedimentation rates decrease during the highstand systems tract (HST), particularly in distal settings dominated by mudstone deposits. This decline is attributed to reduced terrigenous input and the diminished creation of accommodation space as sea level stabilizes or begins to fall (Supplementary Fig. 29). These lithological and sedimentation rate changes further capture broader paleoenvironmental shifts, including sea-level fluctuations and variations in sediment supply. Such changes are consistent with the expected impacts of Milankovitch-scale climatic cycles on depositional systems, highlighting the interplay between orbital forcing, sequence stratigraphy, and sedimentary processes.

The MTM power spectra of the 405-kyr-tuned MS series reveal significant astronomical signals across all three drillcores. In the EYC2 drillcore, prominent peaks occur at 1.9 Myr, 405 kyr, and 30.3-25.2 kyr, all exceeding 95% confidence levels, with the 106 kyr, and 18.6-16.7 kyr signal being less pronounced (Supplementary Fig. 28a). The WD1 drillcore exhibits strong peaks at 5.5 Myr, 2.3 Myr, 1.2 Myr, 405 kyr, 133-103 kyr, 30.2-25 kyr, and 18.5-15.3 kyr, most surpassing 95% confidence levels (Supplementary Fig. 28d). The ZK68 drillcore shows significant periods of 8.5 Myr, 2.8 Myr, 1.4 Myr, 405 kyr, and 132.2-97.7 kyr, all above 99% confidence (Supplementary Fig. 28g). Scargle spectrum analysis of smoothed window averages from the 405-kyr-tuned MS series corroborates these findings in the EYC2, WD1 and ZK68 drillcores (Supplementary Fig. 28b, e, h). These results align with the expected astronomical parameters for the Early Ediacaran, as detailed in Supplementary Note 2 and illustrated in Supplementary Fig. 28.

To summarize, multiple lines of evidence support the astronomical origin of the observed cycles: (1) the frequency ratios align with theoretical Milankovitch periods for the early Ediacaran Period (i,.e., ~405 kyr, ~112.8 kyr, ~30.6 kyr, and ~16.5 kyr cycles)^50,51^, (2) the close matches with all Milankovitch cycles when sedimentation rates are constrained by independent geochronology and the TimeOpt statistical method; (3) the sedimentation rates are consistent with the geological and depositional context (Supplementary Fig. 29); (4) clear evidence exists for eccentricity-modulated precession and long eccentricity-modulated short eccentricity cycles (Supplementary Figs. 25-28), (5) the sensitivity of magnetic susceptibility (MS) to astronomical forcing is well-documented in similar depositional environments^55,56^, and (6) the extended >8 Myr length of the records significantly enhances the statistical robustness of the detected cycles (Fig. 3 and Supplementary Data 2).

**Supplementary Note 4. The Gaskiers glaciation in South China**

The Gaskiers glaciation represents a major climatic event during the Ediacaran period, with evidence widely distributed across at least eight ancient continents^57–59^. Among the Ediacaran glacial deposits, the Gaskiers Formation in Avalonian, Newfoundland, Canada is the most well-known and extensively studied^60,61^. However, pinpointing the precise stratigraphic position of the Gaskiers glaciation in the South China Block proves challenging due to the absence of typical glacial tillites in the local sequence^57,62^. Currently, there are two prevailing viewpoints regarding the stratigraphic position of the Gaskiers glaciation within the Doushantuo Formation in South China. One associates it with the Ediacaran positive carbon isotope excursion 1 (EP1) in Member II of the Doushantuo Formation^62^, while the alternative view places it near the boundary between EN2 and EP2 in the lower parts of Member III^63^, where EP2 represents a positive CIE occurring between EN2 and EN3.

Viewpoint #1 is supported by the discovery of silicified glendonites near the EP1 (Ediacaran positive excursion 1) in the middle part of Member II, indicative of cold conditions^62,64,65^. Viewpoint #2 is corroborated by carbonate clumped isotope paleotemperature reconstructions suggesting relatively low temperatures during the EN2-EP2 transition^63^. A recent study of siliciclastic-hosted δ^13^C_carb_ data from fossiliferous strata in the Conception Group of Newfoundland revealed a CIE below the Gaskiers Formation, which is temporally constrained to before 580.9 ± 0.4 Ma^21,66,67^. This CIE can be correlated with the EN2 event in South China^14,21^. This correlation implies that the Gaskiers glaciation occurred shortly after the EN2 event, aligning with the carbonate-clumped isotope paleotemperature reconstructions by Chang et al. ^63^.

Although the lower Doushantuo Formation have an average sedimentation rate of only ~2 cm/kyr based on published radiometric dating data^68,69^, the silicified glendonites extending vertically over 20 meters in the Zhangcunping section^62,70^ challenge the previously estimated ~390,000-year duration for the Gaskiers glaciation in Newfoundland^67^. Furthermore, considering that the South China Block was located closer to the paleoequator than Newfoundland before and after the Gaskiers glaciation^58,71^, the expression of cold climatic conditions associated with the Gaskiers glaciation may have been relatively shorter in South China compared to Newfoundland. Considering these multiple lines of evidence, the second viewpoint appears more compelling, offering a more consistent interpretation of the available data across different geological contexts.

A notable decline in ^87^Sr/^86^Sr ratios and magnetic susceptibility across multiple sections of South China reveals a significant cooling period around the EN2-EP2 boundary (Supplementary Fig. 30). This period is characterized by markedly reduced terrigenous weathering and input (Supplementary Fig. 30; detailed interpretations of strontium isotope and magnetic susceptibility as paleoclimate proxies are provided in the main text). This observation aligns with a decrease in the Chemical Alteration Index (CIA) at the same stratigraphic level, collectively indicating a shift towards cooler and drier conditions (Supplementary Fig. 30). Based on chemo- and litho-stratigraphic correlation, the significant cooling event near the EN2-EP2 boundary, revealed by the ^87^Sr/^86^Sr ratio, magnetic susceptibility, and CIA is consistent with those indicated by carbonate clumped isotope thermometry (Supplementary Fig. 30). We interpret this period as representing the expression of the cooler climate and significantly reduced continental hydrological cycle during the Gaskiers glaciation. During the interpreted Gaskiers glaciation, lithofacies in the studied drillcores exhibit a transition from calcareous shale and muddy dolostone to dolostone, aligning with a sea-level fall associated with glacial conditions (Figs. 1 and 2; Supplementary Fig. 29). Additionally, reconstructed sedimentation rates at the 405-kyr scale show significantly low values during this period, indicating reduced sediment input (Supplementary Fig. 29). Together, these observations reflect the combined impacts of glacially driven cooling, reduced hydrological activity, and accompanying changes in depositional and weathering processes.

The diamictite of the Gaskiers glaciation was deposited after 580.90 ± 0.40 and before 579.24 ± 0.17 Ma (CA-ID-TIMS dates) on the Avalon and Bonavista Peninsulas, with the 579.63 ± 0.15 Ma age from the lower Rocky Harbour Formation strictly representing a synglacial onset constraint, albeit lacking complete ice coverage, and the 580.90 ± 0.40 Ma age from the Mall Bay Fm providing the maximum constraint^67^. Among these age constraints, we adopt 579.63 ± 0.15 Ma as the anchor point for constructing the Ediacaran astronomical time scale (ATS), due to its lower uncertainty and its significance in marking the global onset of the glaciation. The approximate glaciation termination age of 579.24 ± 0.17 Ma provides an additional constraint for the radioisotopically anchored Ediacaran ATS.

To better constrain the onset of the Gaskiers glaciation in South China, we define its beginning as the initiation of simultaneous significant decreases in magnetic susceptibility and ^87^Sr/^86^Sr ratios, reflecting reduced terrestrial detrital flux and the emergence of weakened continental weathering, respectively (Supplementary Fig. 30). Furthermore, we assess the uncertainty in precisely determining the onset of Gaskiers glaciation in the studied drillcores based on ^87^Sr/^86^Sr and MS data (Supplementary Fig. 30b). These data show a significant declining stage, defined by the transition from the initiation of simultaneous significant decreases in MS and ^87^Sr/^86^Sr records to their minimum values during the glaciation (Supplementary Fig. 30b). This stage is observed over intervals of 1.04 m (MS; 2057.68–2056.54 m) and 1.6 m (^87^Sr/^86^Sr; 2058.56–2056.96 m) in the WD1, and 0.64 m (MS; 1390.70-1390.04 m) and 1.0 m (^87^Sr/^86^Sr; 1391.0–1390.0 m) in the ZK68 (Supplementary Fig. 30b), corresponding to durations of 0.16 Myr, 0.22 Myr, 0.10 Myr, and 0.15 Myr (Supplementary Data 2), respectively. Assuming the Gaskiers glaciation onset occurs at each interval's midpoint (2057.11 m, 2056.76 m, 1390.37 m, and 1390.05 m), the corresponding errors are ±0.08 Myr, ±0.11 Myr, ±0.05 Myr, and ±0.075 Myr. Given the higher resolution of MS data (~0.02 m), we adopt errors of ±0.04 Myr for the WD1 and ±0.025 Myr for the ZK68. The termination of the glaciation can be defined as the point when magnetic susceptibility and ^87^Sr/^86^Sr ratios simultaneously return to their pre-glacial values, reflecting the recovery of continental weathering and an increase in terrestrial detrital flux, marking the transition to post-glacial conditions (Supplementary Fig. 30).

**Supplementary Note 5. Age constraints and stratigraphic correlation of fossil assemblage**

**5.1 Lantian biota**

The Lantian biota represents a diverse assemblage of morphologically varied benthic macrofossils, which are primarily preserved as carbonaceous compression fossils within the black shales of the Ediacaran Lantian Fm Member II in southern Anhui Province, South China^72^. The Lantian biota offers crucial paleontological insights into an early Ediacaran assemblage comprising macroscopic and morphologically diverse eukaryotes, shedding light on their co-evolutionary relationship with fluctuating marine redox conditions^72^. However, direct age constraints for the Lantian biota have been lacking. The lower and upper boundaries of the Lantian biota interval in the Lantian section^72^, are located 26.9 m above the base of Member II and 2.3 m below the Member II/III boundary of the Doushantuo Formation, respectively, with a total thickness of 61.86 m (Supplementary Fig. 31). Considering the paleogeographic proximity of the ZK68 drillcore, situated in a lower slope setting, to the Lantian section in the basin setting of southern Anhui Province^12,73^, we correlate the Lantian section with the ZK68 drillcore based on chemostratigraphic and lithostratigraphic data.

The lower and upper boundary of the Lantian biota could be interpolated to 619.10 ± 0.33 Ma and 582.64 ± 0.42 Ma (For more details on the age interpolation and uncertainty calculation, please refer to Supplementary Note 6.1; Fig. 4) based on our astronomically calibrated ages of 634.90 ± 0.43 Ma for the base of Member II and 581.29 ± 0.43 Ma for the Member II/III boundary by assuming constant sediment accumulation rates between these two age constraint points. Although there are still some uncertainties in this interpolation, this interpolation provides the first reasonably inferred age constraints for the occurrence of the Lantian biota.

**5.2 Weng’an biota**

The Ediacaran Weng'an biota, hosted in phosphorite of the upper Doushantuo Formation in the Weng'an area of central Guizhou Province, South China, preserves exceptional eukaryote fossils. including various acanthomorphic acritarchs, pseudoparenchymatous thalli, tubular microfossils, and spheroidal fossils^74,75^. Radiometric dating of the Doushantuo Formation at Weng'an has yielded whole-rock Pb–Pb isochron ages of 599 ± 4 Ma for Unit 4B^76^, 576 ± 14 Ma for Units 4A and 4B^77^, and 572 ± 36 Ma for Unit 5^78^. Due to the large error bars, the inherent limitations of the Pb-Pb isochron approach, and the fact that the studied samples were collected from a condensed section, these dates are considered unreliable. Thus, direct age constraints for the Weng'an biota are still lacking.

The lower boundary of the Weng’an biota interval is positioned near the negative CIE, known as the WANCE, corresponding to the boundary between Sequence 1/2 in the middle the Doushantuo Formation^12^ (Supplementary Fig. 31). This layer was previously dated at 609 ± 5 Ma in the Zhangcunping section using SIMS zircon U-Pb dating^79^. However, a new CA-ID-TIMS U-Pb analysis of zircon from this sample indicates a detrital origin, with the youngest CA-ID-TIMS date of 612.5 ± 0.9 Ma providing a maximum depositional age^69^. Currently, two schemes have been proposed for the stratigraphic correlation of the Doushantuo Formation between Xiangdangping/Jiulongwan and Zhangchunping sections^12,80^ (Supplementary Fig. 31). Zhu et al.^12^ proposes the negative CIE ca. 16 m above the base of the Doushantuo Formation in the Zhangjunping section corresponds to a horizon ca. 50 m above the Doushantuo base at Xiangdangping section and about 60 m at Jiulongwan section based on the chemo- and sequence-stratigraphic correlation. In contrast, Ouyang et al.^80^ suggests that the same negative CIE corresponds to a horizon about 20.4 m above the base of the Doushantuo Formation in the Jiulongwan section, where a CIE occurred. The latter scheme proposed by Ouyang et al.^80^ is argued to be inconsistent both with the two zircon U-Pb dates from the lower Doushantuo Formation^68^ and astrochronology^81^. A recent Re-Os dating has yielded an age of 587.2 ± 3.6 Ma for a horizon located 58 meters above the base of the Doushantuo Formation at the Jiulongwan section, close to a negative CIE ~20 m below the EN2^69^. Based on the former scheme, Yang et al.^69^ suggests that the new Re-Os date of 587.2 ± 3.6 Ma from the Jiulongwan provides age constraint on the lower boundary of Weng’an biota and the negative CIE ca. 16 m above the base of the Doushantuo Formation in the Zhangchunping section (Supplementary Fig. 31).

In this study, we also adopt the former stratigraphic correlation scheme. We correlate the negative CIE located 16 m above the base of the Doushantuo Formation in the Zhangchunping section and about 60 m above the base at the Jiulongwan section with the nadir of WNACE event 34 m below the Member II/III boundary in the WD1 drillcore. Therefore, based on our astrochronologic data, the lower boundary of the Weng’an biota is constrained to 589.89 ± 0.26 Ma, consistent with the recently obtained Re-Os date of 587.2 ± 3.6 Ma (Fig. 4).

**5.3 Microfossil assemblage zones**

Recent geological and micropaleontological studies of the Ediacaran Doushantuo Formation have revealed a significantly larger and more diverse collection of organic-walled microfossils than previously known^82^. By meticulously examining species ranges and establishing first and last appearance datums, researchers have identified potential new Ediacaran assemblage zones in the Yangtze Gorges area. These findings have implications for the global Ediacaran subdivision. The proposed microfossil assemblage zones, arranged in ascending stratigraphic order, comprise four distinct units: the *Appendisphaera grandis–Weissiella grandistella*–*Tianzhushania spinosa* Assemblage Zone (*A.-W.-T*), *Tanarium tuberosum*–*Schizofusa zangwenlongii* Assemblage Zone (*T.-S.*), *Tanarium conoideum*–*Cavaspina basiconica* Assemblage Zone (*Tc-Cb*), and *Tanarium pycnacanthum*–*Ceratosphaeridium glaberosum* Assemblage Zone (*Tp-Cg*).

The chemostratigraphic and paleontological studies in the Yangtze Gorges area indicate that the base of *A.-W.-T*, *T.-S.*, *Tc-Cb*, *Tp-Cg* and unnamed microfossil zones could be correlated to a level of ~5.0 m above the base of the Member Ⅱ, the zenith of a positive CIE ~35.4 m above the base of Member II, the nadir of the WANCE ~32.1 m below the boundary between Member II and Member Ⅲ, and the nadir of the EN2 at the Xiangdangping section, respectively^12,15,69,82^ (Supplementary Fig. 31). Given the paleogeographic proximity between the WD1 drillcore and the Zhangcunping section, age constraints for these microfossil assemblage zones can be established through chemo- and lithostratigraphic correlation between the two localities.

The lower boundary of the *A.-W.-T.* microfossil assemblage zone is interpolated to be 633.64 ± 0.32 Ma (For more details on the age interpolation and uncertainty calculation, please refer to Supplementary Note 6.2). This is based on two age constraints: (1) a basal age of 634.90 ± 0.43 Ma for Member II in the intrashelf basin setting (this study), and (2) a correlated age of 632.5 ± 0.48 Ma at ~9.5 m above the base of Member II in the Xiangdangping section, which is lithostratigraphically correlated with a horizon 5 m above the base of Member II in the Jijiawan section^68,69^.

We correlate the lower boundary of the *T.-S.* microfossil assemblage zone, corresponding to a positive CIE peak ~35.4 m above the base of Member II, in the Zhangcunping section, with a positive CIE peak ~31.3 m above the base of Member II in the ZK68 drillcore. The latter CIE peak is astronomically calibrated at 611.94 ± 0.43 Ma. Thus, the lower boundary of the *T.-S.* microfossil assemblage zones is constrained to 611.94 ± 0.43 Ma (Fig. 4).

The lower boundary of the *Tc-Cb* microfossil assemblage zone, corresponding to the nadir of a negative CIE (i.e., WANCE) ~32.1 m below the boundary between Member II and Member Ⅲ in the Zhangcunping section, can be correlated to the nadir of the WANCE ~32.2 m below the boundary between Member II and Member Ⅲ in the WD1 drillcore. Our astronomical time scale dates the horizon of the nadir of the WANCE in the WD1 drillcore to 589.89 ± 0.26 Ma. Consequently, the lower boundary of the *Tc-Cb* microfossil assemblage zone is constrained to 589.89 ± 0.26 Ma (Fig. 4).

We correlate the lower boundaries of *Tp-Cg* microfossil assemblage zone in the Zhangcunping section to the nadir of EN2 near the Member II/Ⅲ boundary in the WD1 drillcore (Supplementary Fig. 31). This horizon is astronomically calibrated at 580.25 ± 0.26 Ma. Therefore, the lower boundaries of *Tp-Cg* microfossil assemblage zone 580.25 ± 0.26 Ma (Fig. 4).

**Supplementary Note 6** **Age Interpolation and Uncertainty Calculation**

**6.1 The lower and upper boundary ages of Lantian biota**

**Given:**

Given the ages of 634.90 ± 0.43 Ma at 0 m (i.e., Member Ⅰ/Ⅱ boundary; Fig. 3) and 581.29 ± 0.43 Ma at 91.26 m (i.e., Member Ⅱ/Ⅲ boundary in the slope setting; Fig. 3) in the Doushantuo Formation, calculate the age values at depths of 26.9 m (lower boundary) and 88.96 m (upper boundary)) in the drillcore of Lantian (ref. ^72^), Anhui Province, South China (see Supplementary Fig. 31), using linear interpolation. Also, compute the corresponding age uncertainties using the Weighted Uncertainty Propagation.

Here's the Python code to perform these calculations:

*import math*

*# Given data points with uncertainties*

*z1 = 0.0 # Depth at first point (Member I/II boundary) in meters*

*t1 = 634.90 # Age at first point in Ma (Mega-annum)*

*sigma_t1 = 0.43 # Uncertainty of age at first point in Ma*

*z2 = 91.26 # Depth at second point (Member II/III boundary) in meters*

*t2 = 581.29 # Age at second point in Ma*

*sigma_t2 = 0.43 # Uncertainty of age at second point in Ma*

*# Depths at which to interpolate*

*z_values = [26.9, 88.96] # Depths in meters*

*# Function to perform linear interpolation and uncertainty propagation*

*def interpolate_age(z, z1, t1, sigma_t1, z2, t2, sigma_t2):*

*# Linear interpolation coefficient*

*s = (z - z1) / (z2 - z1)*

*# Interpolated age at depth z*

*t_z = t1 + (t2 - t1) * s*

*# Weighted uncertainty propagation*

*# Since uncertainties in t1 and t2 are independent, and z is exact*

*sigma_tz = math.sqrt(((1 - s) * sigma_t1) ** 2 + (s * sigma_t2) ** 2)*

*return t_z, sigma_tz*

*# Perform calculations and print results*

*for z in z_values:*

*t_z, sigma_tz = interpolate_age(z, z1, t1, sigma_t1, z2, t2, sigma_t2)*

*print(f"At depth {z:.2f} m:")*

*print(f"Interpolated age = {t_z:.2f} ± {sigma_tz:.4f} Ma\n")*

**Output**

*At depth 26.90 m:*

*Interpolated age = 619.10 ± 0.33 Ma*

*At depth 88.96 m:*

*Interpolated age = 582.64 ± 0.42 Ma*

**6.2 The lower boundary of *A.-W.-T.* microfossil assemblage zone**

**Given:**

Given the ages of 634.90 ± 0.43 Ma at 0 m (i.e., Member Ⅰ/Ⅱ boundary; Fig. 3) and 632.5 ± 0.48 Ma^69^ (Supplementary Fig. 31) at 9.5 m above the Member Ⅰ/Ⅱ boundary in the Doushantuo Formation, calculate the age value at a depth of ~5 m (corresponding to the lower boundary of *A.-W.-T.* microfossil assemblage zone; Supplementary Fig. 31) using linear interpolation. Also, compute the corresponding age uncertainty using the Weighted Uncertainty Propagation.

Here's the Python code to perform these calculations:

*import math*

*# Given data points with uncertainties*

*z1 = 0.0 # Depth at first point (Member I/II boundary) in meters*

*t1 = 634.90 # Age at first point in Ma (Mega-annum)*

*sigma_t1 = 0.43 # Uncertainty of age at first point in Ma*

*z2 = 9.5 # Depth at second point (9.5 m above Member I/II boundary) in meters*

*t2 = 632.5 # Age at second point in Ma*

*sigma_t2 = 0.48 # Uncertainty of age at second point in Ma*

*# Depth at which to interpolate*

*z = 5.0 # Depth in meters (lower boundary of A.-W.-T. microfossil assemblage zone)*

*# Function to perform linear interpolation and uncertainty propagation*

*def interpolate_age(z, z1, t1, sigma_t1, z2, t2, sigma_t2):*

*# Linear interpolation coefficient*

*s = (z - z1) / (z2 - z1)*

*# Interpolated age at depth z*

*t_z = t1 + (t2 - t1) * s*

*# Weighted uncertainty propagation*

*# Since uncertainties in t1 and t2 are independent, and z is exact*

*sigma_tz = math.sqrt(((1 - s) * sigma_t1) ** 2 + (s * sigma_t2) ** 2)*

*return t_z, sigma_tz*

*# Perform calculation and print result*

*t_z, sigma_tz = interpolate_age(z, z1, t1, sigma_t1, z2, t2, sigma_t2)*

*print(f"At depth {z:.2f} m:")*

*print(f"Interpolated age = {t_z:.2f} ± {sigma_tz:.4f} Ma")*

**Output**

*At depth 5.00 m:*

*Interpolated age = 633.64 ± 0.32 Ma*

**Supplementary Text 1** **Settings and outputs of the TimeOpt analysis**

**1.1 EYC2 drillcore**

> # (1) Load the package Astrochron

> library(astrochron)

>

> # (2) Obtained the MS data (3123.35-3154.37 m) in the EYC2 drillcore

> dat <- read()

----- READ STRATIGRAPHIC SERIES FROM DATA FILE -----

The following options are selected:

* What type of column delimiter are you using?: Comma

* Does your file have column titles/headers?: auto detect

<PLEASE CHOOSE YOUR FILE>

* No column titles/headers detected

* Number of stratigraphic samples (rows)= 1552

* Number of variables (columns)= 1 (excluding depth/height/time)

* Sorting data into increasing depth/height/time order.

Will remove empty entries (from either column).

* Number of samples (rows) post-sorting= 1552

* Mean sampling interval= 0.02

* Median sampling interval= 0.02

* Maximum sampling interval= 0.02

* Minimum sampling interval= 0.02

* First 3 lines of data file:

V1 V2

1 3123.35 0.3870

2 3123.37 0.3646

3 3123.39 0.3498

>

> # (3) Isolate data of interest, between 3124.9 and 3127.0 m

> dat1=iso(dat,xmin=3124.9,xmax=3127.0);

----- ISOLATE STRATIGRAPHIC DATA BY LOCATION -----

* Number of data points= 1552

* Number of columns= 2

* Minimum= 3123.35 , Maximum= 3154.37

* Isolating data between 3124.9 and 3127

* Number of data points following culling= 105

>

> # (4) Define the target periods from Waltham (2015)

> targetP=c(19.8,18.9,16.5,16.4)

>

> # (5) run nominal timeOpt

> # output sedimentation rate grid and fit

> res1=timeOpt(dat1,sedmin=.29,sedmax=0.45,numsed=100,targetP=targetP,flow=1/19.8-0.01,fhigh=1/16.4+0.01,roll=10^7,limit=T,output=1);

----- TimeOpt: Assessment of Amplitude Modulation & Bundling-----

* Number of data points in stratigraphic series: 105

* Stratigraphic series length (meters): 2.08

* Sampling interval (meters): 0.02

* Linear trend subtracted. m= -0.01016544 b= 31.88394

* Using default eccentricity target periods (ka)= 405.6795 130.719 123.839 98.86307 94.87666

* PLEASE WAIT: Performing Optimization

0% 25% 50% 75% 100%

===========================================

* Maximum (spectral power r^2)= 0.723037 at sedimentation rate of 0.3572619 cm/ka

* Maximum (envelope r^2)= 0.6421451 at sedimentation rate of 0.3478742 cm/ka

* Maximum (envelope r^2) x (spectral power r^2) = 0.4562411 at sedimentation rate of 0.3556799 cm/ka

* Plotting/outputting time series calibrated to (envelope r^2) x (spectral power r^2) maximum

>

> # output optimal time series, bandpassed series, amplitude envelope and

> # TimeOpt-reconstructed eccentricity

> res2=timeOpt(dat1,sedmin=.29,sedmax=0.45,numsed=100,targetP=targetP,flow=1/19.8-0.01,fhigh=1/16.4+0.01,roll=10^7,limit=T,output=2);

----- TimeOpt: Assessment of Amplitude Modulation & Bundling-----

* Number of data points in stratigraphic series: 105

* Stratigraphic series length (meters): 2.08

* Sampling interval (meters): 0.02

* Linear trend subtracted. m= -0.01016544 b= 31.88394

* Using default eccentricity target periods (ka)= 405.6795 130.719 123.839 98.86307 94.87666

* PLEASE WAIT: Performing Optimization

0% 25% 50% 75% 100%

===========================================

* Maximum (spectral power r^2)= 0.723037 at sedimentation rate of 0.3572619 cm/ka

* Maximum (envelope r^2)= 0.6421451 at sedimentation rate of 0.3478742 cm/ka

* Maximum (envelope r^2) x (spectral power r^2) = 0.4562411 at sedimentation rate of 0.3556799 cm/ka

* Plotting/outputting time series calibrated to (envelope r^2) x (spectral power r^2) maximum

>

> # (6) perform nominal timeOpt significance testing (this function call uses

> # (this function call uses 9 cores for parallel processing)

> simres=timeOptSim(dat1,sedmin=.29,sedmax=0.45,numsed=100,targetP=targetP,flow=1/19.8-0.01,fhigh=1/16.4+0.01,roll=10^7,numsim=2000,output=2,ncores=9);

----- TimeOpt Monte Carlo Simulation -----

* Number of data points in stratigraphic series: 105

* Stratigraphic series length (meters): 2.08

* Sampling interval (meters): 0.02

* Linear trend subtracted. m= -0.01016544 b= 31.88394

* Raw AR1 = 0.7735907

* (Envelope r^2) x (Spectral Power r^2) = 0.4562411

* PLEASE WAIT: Performing 2000 simulations using 9 cores

* (Envelope r^2) * (Spectral Power r^2) p-value = 0.062

> # (7) plot summary figure

> timeOptPlot(dat1,res1,res2,simres,flow=1/19.8-0.01,fhigh=1/16.4+0.01,roll=10^7,targetE=c(405,132,125,95),targetP=targetP,xlab="Height(m)",ylab="MS (×10-3 SI)",fitR=0.4562411,verbose=T);

----- Generating summary plot for TimeOpt analysis----

**** WARNING: using default height and width of 7 inches.

>

**1.2 The Subset D2-1 in the WD1 drillcore**

> # (1) Load the package Astrochron

> library(astrochron)

>

> # (2) Obtained the MS data of Subset D2-1 (2132-2155.4 m) in the WD1 drillcore

> dat <- read()

----- READ STRATIGRAPHIC SERIES FROM DATA FILE -----

The following options are selected:

* What type of column delimiter are you using?: Comma

* Does your file have column titles/headers?: auto detect

<PLEASE CHOOSE YOUR FILE>

* No column titles/headers detected

* Number of stratigraphic samples (rows)= 1171

* Number of variables (columns)= 1 (excluding depth/height/time)

* Sorting data into increasing depth/height/time order.

Will remove empty entries (from either column).

* Number of samples (rows) post-sorting= 1171

* Mean sampling interval= 0.02

* Median sampling interval= 0.02

* Maximum sampling interval= 0.02

* Minimum sampling interval= 0.02

* First 3 lines of data file:

V1 V2

1 2132.00 0.208

2 2132.02 0.232

3 2132.04 0.228

> # (3) Isolate data of interest, between 2143.25 and 2145.5 m

> dat1=iso(dat,xmin=2143.25,xmax=2145.5);

----- ISOLATE STRATIGRAPHIC DATA BY LOCATION -----

* Number of data points= 1171

* Number of columns= 2

* Minimum= 2132 , Maximum= 2155.4

* Isolating data between 2143.25 and 2145.5

* Number of data points following culling= 113

>

> # (4) Define the target periods from Waltham (2015)

> targetP=c(20,19.1,16.6,16.5)

>

> # (5) run nominal timeOpt

> # output sedimentation rate grid and fit

> res1=timeOpt(dat1,sedmin=.29,sedmax=0.45,numsed=100,targetP=targetP,flow=1/20-0.01,fhigh=1/16.5+0.01,roll=10^7,limit=T,output=1);

----- TimeOpt: Assessment of Amplitude Modulation & Bundling-----

* Number of data points in stratigraphic series: 113

* Stratigraphic series length (meters): 2.24

* Sampling interval (meters): 0.02

* Linear trend subtracted. m= -0.01218515 b= 26.29153

* Using default eccentricity target periods (ka)= 405.6795 130.719 123.839 98.86307 94.87666

* PLEASE WAIT: Performing Optimization

0% 25% 50% 75% 100%

===========================================

* Maximum (spectral power r^2)= 0.3843546 at sedimentation rate of 0.4210185 cm/ka

* Maximum (envelope r^2)= 0.9082488 at sedimentation rate of 0.366903 cm/ka

* Maximum (envelope r^2) x (spectral power r^2) = 0.3237431 at sedimentation rate of 0.3718206 cm/ka

* Plotting/outputting time series calibrated to (envelope r^2) x (spectral power r^2) maximum

>

> # output optimal time series, bandpassed series, amplitude envelope and

> # TimeOpt-reconstructed eccentricity

> res2=timeOpt(dat1,sedmin=.29,sedmax=0.45,numsed=100,targetP=targetP,flow=1/20-0.01,fhigh=1/16.5+0.01,roll=10^7,limit=T,output=2);

----- TimeOpt: Assessment of Amplitude Modulation & Bundling-----

* Number of data points in stratigraphic series: 113

* Stratigraphic series length (meters): 2.24

* Sampling interval (meters): 0.02

* Linear trend subtracted. m= -0.01218515 b= 26.29153

* Using default eccentricity target periods (ka)= 405.6795 130.719 123.839 98.86307 94.87666

* PLEASE WAIT: Performing Optimization

0% 25% 50% 75% 100%

===========================================

* Maximum (spectral power r^2)= 0.3843546 at sedimentation rate of 0.4210185 cm/ka

* Maximum (envelope r^2)= 0.9082488 at sedimentation rate of 0.366903 cm/ka

* Maximum (envelope r^2) x (spectral power r^2) = 0.3237431 at sedimentation rate of 0.3718206 cm/ka

* Plotting/outputting time series calibrated to (envelope r^2) x (spectral power r^2) maximum

>

> # (6) perform nominal timeOpt significance testing (this function call uses

> # (this function call uses 9 cores for parallel processing)

> simres=timeOptSim(dat1,sedmin=.29,sedmax=0.45,numsed=100,targetP=targetP,flow=1/20-0.01,fhigh=1/16.5+0.01,roll=10^7,numsim=2000,output=2,ncores=9);

----- TimeOpt Monte Carlo Simulation -----

* Number of data points in stratigraphic series: 113

* Stratigraphic series length (meters): 2.24

* Sampling interval (meters): 0.02

* Linear trend subtracted. m= -0.01218515 b= 26.29153

* Raw AR1 = 0.3798388

* (Envelope r^2) x (Spectral Power r^2) = 0.3237431

* PLEASE WAIT: Performing 2000 simulations using 9 cores

* (Envelope r^2) * (Spectral Power r^2) p-value = 0.006

> # (7) plot summary figure

> timeOptPlot(dat1,res1,res2,simres,flow=1/20.1-0.01,fhigh=1/16.6+0.01,roll=10^7,targetE=c(405,125,95),targetP=targetP,xlab="Height(m)",ylab="MS (×10-3 SI)",fitR= 0.32374,verbose=T);

----- Generating summary plot for TimeOpt analysis----

**** WARNING: using default height and width of 7 inches.

>

**1.3 The Subset D2-2 in the WD1 drillcore**

> # (1) Load the package Astrochron

> library(astrochron)

>

> # (2) Obtained the MS data of Subset D2-2 (2110-2132 m) in the WD1 drillcore

> dat <- read()

----- READ STRATIGRAPHIC SERIES FROM DATA FILE -----

The following options are selected:

* What type of column delimiter are you using?: Comma

* Does your file have column titles/headers?: auto detect

<PLEASE CHOOSE YOUR FILE>

* No column titles/headers detected

* Number of stratigraphic samples (rows)= 1101

* Number of variables (columns)= 1 (excluding depth/height/time)

* Sorting data into increasing depth/height/time order.

Will remove empty entries (from either column).

* Number of samples (rows) post-sorting= 1101

* Mean sampling interval= 0.02

* Median sampling interval= 0.02

* Maximum sampling interval= 0.02

* Minimum sampling interval= 0.02

* First 3 lines of data file:

V1 V2

1 2110.00 0.140

2 2110.02 0.166

3 2110.04 0.152

>

> # (3) Isolate data of interest, between 2116.4 and 2118.8 m

> dat1=iso(dat,xmin=2116.4,xmax=2118.8);

----- ISOLATE STRATIGRAPHIC DATA BY LOCATION -----

* Number of data points= 1101

* Number of columns= 2

* Minimum= 2110 , Maximum= 2132

* Isolating data between 2116.4 and 2118.8

* Number of data points following culling= 121

>

> # (4) Define the target periods from Waltham (2015)

> targetP=c(20,19.1,16.6,16.5)

>

> # (5) run nominal timeOpt

> # output sedimentation rate grid and fit

> res1=timeOpt(dat1,sedmin=.29,sedmax=0.50,numsed=100,targetP=targetP,flow=1/20-0.01,fhigh=1/16.5+0.01,roll=10^7,limit=T,output=1);

----- TimeOpt: Assessment of Amplitude Modulation & Bundling-----

* Number of data points in stratigraphic series: 121

* Stratigraphic series length (meters): 2.4

* Sampling interval (meters): 0.02

* Linear trend subtracted. m= -0.01173449 b= 25.01501

* Using default eccentricity target periods (ka)= 405.6795 130.719 123.839 98.86307 94.87666

* PLEASE WAIT: Performing Optimization

0% 25% 50% 75% 100%

===========================================

* Maximum (spectral power r^2)= 0.5367444 at sedimentation rate of 0.4079005 cm/ka

* Maximum (envelope r^2)= 0.6351202 at sedimentation rate of 0.3574402 cm/ka

* Maximum (envelope r^2) x (spectral power r^2) = 0.3291043 at sedimentation rate of 0.3554789 cm/ka

* Plotting/outputting time series calibrated to (envelope r^2) x (spectral power r^2) maximum

>

> # output optimal time series, bandpassed series, amplitude envelope and

> # TimeOpt-reconstructed eccentricity

> res2=timeOpt(dat1,sedmin=.29,sedmax=0.50,numsed=100,targetP=targetP,flow=1/20-0.01,fhigh=1/16.5+0.01,roll=10^7,limit=T,output=2);

----- TimeOpt: Assessment of Amplitude Modulation & Bundling-----

* Number of data points in stratigraphic series: 121

* Stratigraphic series length (meters): 2.4

* Sampling interval (meters): 0.02

* Linear trend subtracted. m= -0.01173449 b= 25.01501

* Using default eccentricity target periods (ka)= 405.6795 130.719 123.839 98.86307 94.87666

* PLEASE WAIT: Performing Optimization

0% 25% 50% 75% 100%

===========================================

* Maximum (spectral power r^2)= 0.5367444 at sedimentation rate of 0.4079005 cm/ka

* Maximum (envelope r^2)= 0.6351202 at sedimentation rate of 0.3574402 cm/ka

* Maximum (envelope r^2) x (spectral power r^2) = 0.3291043 at sedimentation rate of 0.3554789 cm/ka

* Plotting/outputting time series calibrated to (envelope r^2) x (spectral power r^2) maximum

>

> # (6) perform nominal timeOpt significance testing (this function call uses

> # (this function call uses 9 cores for parallel processing)

> simres=timeOptSim(dat1,sedmin=.29,sedmax=0.50,numsed=100,targetP=targetP,flow=1/20-0.01,fhigh=1/16.5+0.01,roll=10^7,numsim=2000,output=2,ncores=9);

----- TimeOpt Monte Carlo Simulation -----

* Number of data points in stratigraphic series: 121

* Stratigraphic series length (meters): 2.4

* Sampling interval (meters): 0.02

* Linear trend subtracted. m= -0.01173449 b= 25.01501

* Raw AR1 = 0.503233

* (Envelope r^2) x (Spectral Power r^2) = 0.3291043

* PLEASE WAIT: Performing 2000 simulations using 9 cores

* (Envelope r^2) * (Spectral Power r^2) p-value = 0.016

>

> # (7) plot summary figure

> timeOptPlot(dat1,res1,res2,simres,flow=1/20.1-0.01,fhigh=1/16.6+0.01,roll=10^7,targetE=c(405,125,95),targetP=targetP,xlab="Height(m)",ylab="MS (×10-3 SI)",fitR= 0.3291043,verbose=T);

----- Generating summary plot for TimeOpt analysis----

**** WARNING: using default height and width of 7 inches.

>

**1.4 The Subset D2-3 in the WD1 drillcore**

> # (1) Load the package Astrochron

> library(astrochron)

>

> # (2) Obtained the MS data of Subset D2-3 (2088-2110 m) in the WD1 drillcore

> dat <- read()

----- READ STRATIGRAPHIC SERIES FROM DATA FILE -----

The following options are selected:

* What type of column delimiter are you using?: Comma

* Does your file have column titles/headers?: auto detect

<PLEASE CHOOSE YOUR FILE>

* No column titles/headers detected

* Number of stratigraphic samples (rows)= 1101

* Number of variables (columns)= 1 (excluding depth/height/time)

* Sorting data into increasing depth/height/time order.

Will remove empty entries (from either column).

* Number of samples (rows) post-sorting= 1101

* Mean sampling interval= 0.02

* Median sampling interval= 0.02

* Maximum sampling interval= 0.02

* Minimum sampling interval= 0.02

* First 3 lines of data file:

V1 V2

1 2088.00 0.176

2 2088.02 0.190

3 2088.04 0.146

>

> # (3) Isolate data of interest, between 2091.1 and 2094.8 m

> dat1=iso(dat,xmin=2091.1,xmax=2094.8);

----- ISOLATE STRATIGRAPHIC DATA BY LOCATION -----

* Number of data points= 1101

* Number of columns= 2

* Minimum= 2088 , Maximum= 2110

* Isolating data between 2091.1 and 2094.8

* Number of data points following culling= 186

>

> # (4) Define the target periods from Waltham (2015)

> targetP=c(20.1,19.1,16.7,16.6)

>

> # (5) run nominal timeOpt

> # output sedimentation rate grid and fit

> res1=timeOpt(dat1,sedmin=.29,sedmax=0.65,numsed=100,targetP=targetP,flow=1/20.1-0.01,fhigh=1/16.6+0.01,roll=10^7,limit=T,output=1);

----- TimeOpt: Assessment of Amplitude Modulation & Bundling-----

* Number of data points in stratigraphic series: 186

* Stratigraphic series length (meters): 3.7

* Sampling interval (meters): 0.02

* Linear trend subtracted. m= 0.0484975 b= -101.2935

* Using default eccentricity target periods (ka)= 405.6795 130.719 123.839 98.86307 94.87666

* PLEASE WAIT: Performing Optimization

0% 25% 50% 75% 100%

===========================================

* Maximum (spectral power r^2)= 0.3381762 at sedimentation rate of 0.4653186 cm/ka

* Maximum (envelope r^2)= 0.6032959 at sedimentation rate of 0.4846779 cm/ka

* Maximum (envelope r^2) x (spectral power r^2) = 0.2031225 at sedimentation rate of 0.4886454 cm/ka

* Plotting/outputting time series calibrated to (envelope r^2) x (spectral power r^2) maximum

>

> # output optimal time series, bandpassed series, amplitude envelope and

> # TimeOpt-reconstructed eccentricity

> res2=timeOpt(dat1,sedmin=.29,sedmax=0.65,numsed=100,targetP=targetP,flow=1/20.1-0.01,fhigh=1/16.6+0.01,roll=10^7,limit=T,output=2);

----- TimeOpt: Assessment of Amplitude Modulation & Bundling-----

* Number of data points in stratigraphic series: 186

* Stratigraphic series length (meters): 3.7

* Sampling interval (meters): 0.02

* Linear trend subtracted. m= 0.0484975 b= -101.2935

* Using default eccentricity target periods (ka)= 405.6795 130.719 123.839 98.86307 94.87666

* PLEASE WAIT: Performing Optimization

0% 25% 50% 75% 100%

===========================================

* Maximum (spectral power r^2)= 0.3381762 at sedimentation rate of 0.4653186 cm/ka

* Maximum (envelope r^2)= 0.6032959 at sedimentation rate of 0.4846779 cm/ka

* Maximum (envelope r^2) x (spectral power r^2) = 0.2031225 at sedimentation rate of 0.4886454 cm/ka

* Plotting/outputting time series calibrated to (envelope r^2) x (spectral power r^2) maximum

>

> # (6) perform nominal timeOpt significance testing (this function call uses

> # (this function call uses 9 cores for parallel processing)

> simres=timeOptSim(dat1,sedmin=.29,sedmax=0.65,numsed=100,targetP=targetP,flow=1/20.1-0.01,fhigh=1/16.6+0.01,roll=10^7,numsim=2000,output=2,ncores=9);

----- TimeOpt Monte Carlo Simulation -----

* Number of data points in stratigraphic series: 186

* Stratigraphic series length (meters): 3.7

* Sampling interval (meters): 0.02

* Linear trend subtracted. m= 0.0484975 b= -101.2935

* Raw AR1 = 0.550077

* (Envelope r^2) x (Spectral Power r^2) = 0.2031225

* PLEASE WAIT: Performing 2000 simulations using 9 cores

* (Envelope r^2) * (Spectral Power r^2) p-value = 0.0525

> # (7) plot summary figure

> timeOptPlot(dat1,res1,res2,simres,flow=1/20.1-0.01,fhigh=1/16.6+0.01,roll=10^7,targetE=c(405,125,95),targetP=targetP,xlab="Height(m)",ylab="MS (×10-3 SI)",fitR= 0.2031225,verbose=T);

----- Generating summary plot for TimeOpt analysis----

**** WARNING: using default height and width of 7 inches.

>

**1.5 The Subset D2-4 in the WD1 drillcore**

> # (1) Load the package Astrochron

> library(astrochron)

>

> # (2) Obtained the MS data of Subset D2-4 (2067.2-2088 m) in the WD1 drillcore

> dat <- read()

----- READ STRATIGRAPHIC SERIES FROM DATA FILE -----

The following options are selected:

* What type of column delimiter are you using?: Comma

* Does your file have column titles/headers?: auto detect

<PLEASE CHOOSE YOUR FILE>

* No column titles/headers detected

* Number of stratigraphic samples (rows)= 1038

* Number of variables (columns)= 1 (excluding depth/height/time)

* Sorting data into increasing depth/height/time order.

Will remove empty entries (from either column).

* Number of samples (rows) post-sorting= 1038

* Mean sampling interval= 0.02

* Median sampling interval= 0.02

* Maximum sampling interval= 0.02

* Minimum sampling interval= 0.02

* First 3 lines of data file:

V1 V2

1 2067.26 0.144

2 2067.28 0.171

3 2067.30 0.194

>

> # (3) Isolate data of interest, between 2070.3 and 2072.8 m

> dat1=iso(dat,xmin=2070.3,xmax=2072.8);

----- ISOLATE STRATIGRAPHIC DATA BY LOCATION -----

* Number of data points= 1038

* Number of columns= 2

* Minimum= 2067.26 , Maximum= 2088

* Isolating data between 2070.3 and 2072.8

* Number of data points following culling= 126

>

> # (4) Define the target periods from Waltham (2015)

> targetP=c(20.1,19.1,16.7,16.6)

>

> # (5) run nominal timeOpt

> # output sedimentation rate grid and fit

> res1=timeOpt(dat1,sedmin=.29,sedmax=0.45,numsed=100,targetP=targetP,flow=1/20.1-0.01,fhigh=1/16.6+0.01,roll=10^7,limit=T,output=1);

----- TimeOpt: Assessment of Amplitude Modulation & Bundling-----

* Number of data points in stratigraphic series: 126

* Stratigraphic series length (meters): 2.5

* Sampling interval (meters): 0.02

* Linear trend subtracted. m= -0.04326844 b= 89.85181

* Using default eccentricity target periods (ka)= 405.6795 130.719 123.839 98.86307 94.87666

* PLEASE WAIT: Performing Optimization

0% 25% 50% 75% 100%

===========================================

* Maximum (spectral power r^2)= 0.4587374 at sedimentation rate of 0.3718206 cm/ka

* Maximum (envelope r^2)= 0.6998962 at sedimentation rate of 0.3718206 cm/ka

* Maximum (envelope r^2) x (spectral power r^2) = 0.3210686 at sedimentation rate of 0.3718206 cm/ka

* Plotting/outputting time series calibrated to (envelope r^2) x (spectral power r^2) maximum

>

> # output optimal time series, bandpassed series, amplitude envelope and

> # TimeOpt-reconstructed eccentricity

> res2=timeOpt(dat1,sedmin=.29,sedmax=0.45,numsed=100,targetP=targetP,flow=1/20.1-0.01,fhigh=1/16.6+0.01,roll=10^7,limit=T,output=2);

----- TimeOpt: Assessment of Amplitude Modulation & Bundling-----

* Number of data points in stratigraphic series: 126

* Stratigraphic series length (meters): 2.5

* Sampling interval (meters): 0.02

* Linear trend subtracted. m= -0.04326844 b= 89.85181

* Using default eccentricity target periods (ka)= 405.6795 130.719 123.839 98.86307 94.87666

* PLEASE WAIT: Performing Optimization

0% 25% 50% 75% 100%

===========================================

* Maximum (spectral power r^2)= 0.4587374 at sedimentation rate of 0.3718206 cm/ka

* Maximum (envelope r^2)= 0.6998962 at sedimentation rate of 0.3718206 cm/ka

* Maximum (envelope r^2) x (spectral power r^2) = 0.3210686 at sedimentation rate of 0.3718206 cm/ka

* Plotting/outputting time series calibrated to (envelope r^2) x (spectral power r^2) maximum

>

> # (6) perform nominal timeOpt significance testing (this function call uses

> # (this function call uses 9 cores for parallel processing)

> simres=timeOptSim(dat1,sedmin=.29,sedmax=0.45,numsed=100,targetP=targetP,flow=1/20.1-0.01,fhigh=1/16.6+0.01,roll=10^7,numsim=2000,output=2,ncores=9);

----- TimeOpt Monte Carlo Simulation -----

* Number of data points in stratigraphic series: 126

* Stratigraphic series length (meters): 2.5

* Sampling interval (meters): 0.02

* Linear trend subtracted. m= -0.04326844 b= 89.85181

* Raw AR1 = 0.6199614

* (Envelope r^2) x (Spectral Power r^2) = 0.3210686

* PLEASE WAIT: Performing 2000 simulations using 9 cores

* (Envelope r^2) * (Spectral Power r^2) p-value = 0.021

> # (7) plot summary figure

> timeOptPlot(dat1,res1,res2,simres,flow=1/20.1-0.01,fhigh=1/16.6+0.01,roll=10^7,targetE=c(405,125,95),targetP=targetP,xlab="Height(m)",ylab="MS (×10-3 SI)",fitR=0.3210686,verbose=T);

----- Generating summary plot for TimeOpt analysis----

**** WARNING: using default height and width of 7 inches.

>

**1.6 The Subset D3 in the WD1 drillcore**

> # (1) Load the package Astrochron

> library(astrochron)

>

> # (2) Obtained the MS data of Subset D3 (2040-2067.2 m) in the WD1 drillcore

> dat <- read()

----- READ STRATIGRAPHIC SERIES FROM DATA FILE -----

The following options are selected:

* What type of column delimiter are you using?: Comma

* Does your file have column titles/headers?: auto detect

<PLEASE CHOOSE YOUR FILE>

* No column titles/headers detected

* Number of stratigraphic samples (rows)= 1364

* Number of variables (columns)= 1 (excluding depth/height/time)

* Sorting data into increasing depth/height/time order.

Will remove empty entries (from either column).

* Number of samples (rows) post-sorting= 1364

* Mean sampling interval= 0.02

* Median sampling interval= 0.02

* Maximum sampling interval= 0.02

* Minimum sampling interval= 0.02

* First 3 lines of data file:

V1 V2

1 2040.00 0.221

2 2040.02 0.279

3 2040.04 0.214

>

> # (3) Isolate data of interest, between 2051.2-2054.8 m

> dat1=iso(dat,xmin=2051.2,xmax=2054.8);

----- ISOLATE STRATIGRAPHIC DATA BY LOCATION -----

* Number of data points= 1364

* Number of columns= 2

* Minimum= 2040 , Maximum= 2067.26

* Isolating data between 2051.2 and 2054.8

* Number of data points following culling= 181

>

> # (4) Define the target periods from Waltham (2015)

> targetP=c(20.1,19.1,16.7,16.6)

>

> # (5) run nominal timeOpt

> # output sedimentation rate grid and fit

> res1=timeOpt(dat1,sedmin=.29,sedmax=0.75,numsed=100,targetP=targetP,flow=1/20.1-0.01,fhigh=1/16.6+0.01,roll=10^7,limit=T,output=1);

----- TimeOpt: Assessment of Amplitude Modulation & Bundling-----

* Number of data points in stratigraphic series: 181

* Stratigraphic series length (meters): 3.6

* Sampling interval (meters): 0.02

* Linear trend subtracted. m= -0.01286665 b= 26.5464

* Using default eccentricity target periods (ka)= 405.6795 130.719 123.839 98.86307 94.87666

* PLEASE WAIT: Performing Optimization

0% 25% 50% 75% 100%

===========================================

* Maximum (spectral power r^2)= 0.6520236 at sedimentation rate of 0.721752 cm/ka

* Maximum (envelope r^2)= 0.7356797 at sedimentation rate of 0.6432328 cm/ka

* Maximum (envelope r^2) x (spectral power r^2) = 0.4323877 at sedimentation rate of 0.6494362 cm/ka

* Plotting/outputting time series calibrated to (envelope r^2) x (spectral power r^2) maximum

>

> # output optimal time series, bandpassed series, amplitude envelope and

> # TimeOpt-reconstructed eccentricity

> res2=timeOpt(dat1,sedmin=.29,sedmax=0.75,numsed=100,targetP=targetP,flow=1/20.1-0.01,fhigh=1/16.6+0.01,roll=10^7,limit=T,output=2);

----- TimeOpt: Assessment of Amplitude Modulation & Bundling-----

* Number of data points in stratigraphic series: 181

* Stratigraphic series length (meters): 3.6

* Sampling interval (meters): 0.02

* Linear trend subtracted. m= -0.01286665 b= 26.5464

* Using default eccentricity target periods (ka)= 405.6795 130.719 123.839 98.86307 94.87666

* PLEASE WAIT: Performing Optimization

0% 25% 50% 75% 100%

===========================================

* Maximum (spectral power r^2)= 0.6520236 at sedimentation rate of 0.721752 cm/ka

* Maximum (envelope r^2)= 0.7356797 at sedimentation rate of 0.6432328 cm/ka

* Maximum (envelope r^2) x (spectral power r^2) = 0.4323877 at sedimentation rate of 0.6494362 cm/ka

* Plotting/outputting time series calibrated to (envelope r^2) x (spectral power r^2) maximum

>

> # (6) perform nominal timeOpt significance testing (this function call uses

> # (this function call uses 9 cores for parallel processing)

> simres=timeOptSim(dat1,sedmin=.29,sedmax=0.75,numsed=100,targetP=targetP,flow=1/20.1-0.01,fhigh=1/16.6+0.01,roll=10^7,numsim=2000,output=2,ncores=9);

----- TimeOpt Monte Carlo Simulation -----

* Number of data points in stratigraphic series: 181

* Stratigraphic series length (meters): 3.6

* Sampling interval (meters): 0.02

* Linear trend subtracted. m= -0.01286665 b= 26.5464

* Raw AR1 = 0.8065303

* (Envelope r^2) x (Spectral Power r^2) = 0.4323877

* PLEASE WAIT: Performing 2000 simulations using 9 cores

* (Envelope r^2) * (Spectral Power r^2) p-value = 0.019

>

> # (7) plot summary figure

> timeOptPlot(dat1,res1,res2,simres,flow=1/20.1-0.01,fhigh=1/16.6+0.01,roll=10^7,targetE=c(405,125,95),targetP=targetP,xlab="Height(m)",ylab="MS (×10-3 SI)",fitR=0.4323877,verbose=T);

----- Generating summary plot for TimeOpt analysis----

**** WARNING: using default height and width of 7 inches.

>

**1.7 The Subset D1 in the ZK68 drillcore**

> # (1) Load the package Astrochron

> library(astrochron)

>

> # (2) Obtained the MS data of Subset D1 (1479.5-1472 m) in the ZK68 drillcore

> dat <- read()

----- READ STRATIGRAPHIC SERIES FROM DATA FILE -----

The following options are selected:

* What type of column delimiter are you using?: Comma

* Does your file have column titles/headers?: auto detect

<PLEASE CHOOSE YOUR FILE>

* No column titles/headers detected

* Number of stratigraphic samples (rows)= 376

* Number of variables (columns)= 1 (excluding depth/height/time)

* Sorting data into increasing depth/height/time order.

Will remove empty entries (from either column).

* Number of samples (rows) post-sorting= 376

* Mean sampling interval= 0.02

* Median sampling interval= 0.02

* Maximum sampling interval= 0.02

* Minimum sampling interval= 0.02

* First 3 lines of data file:

V1 V2

1 1472.00 0.04900000

2 1472.02 0.05331667

3 1472.04 0.05301250

>

> # (3) Isolate data of interest, between 1472.7 and 1476.2 m

> dat1=iso(dat,xmin=1472.7,xmax=1476.2);

----- ISOLATE STRATIGRAPHIC DATA BY LOCATION -----

* Number of data points= 376

* Number of columns= 2

* Minimum= 1472 , Maximum= 1479.5

* Isolating data between 1472.7 and 1476.2

* Number of data points following culling= 176

>

> # (4) Define the target periods from Waltham (2015)

> targetP=c(19.8,18.9,16.5,16.4)

>

> # (5) run nominal timeOpt

> # output sedimentation rate grid and fit

> res1=timeOpt(dat1,sedmin=.29,sedmax=0.80,numsed=100,targetP=targetP,flow=1/19.8-0.01,fhigh=1/16.4+0.01,roll=10^7,limit=T,output=1);

----- TimeOpt: Assessment of Amplitude Modulation & Bundling-----

* Number of data points in stratigraphic series: 176

* Stratigraphic series length (meters): 3.5

* Sampling interval (meters): 0.02

* Linear trend subtracted. m= -0.002182244 b= 3.313136

* Using default eccentricity target periods (ka)= 405.6795 130.719 123.839 98.86307 94.87666

* PLEASE WAIT: Performing Optimization

0% 25% 50% 75% 100%

===========================================

* Maximum (spectral power r^2)= 0.5989703 at sedimentation rate of 0.7220639 cm/ka

* Maximum (envelope r^2)= 0.8253843 at sedimentation rate of 0.6517204 cm/ka

* Maximum (envelope r^2) x (spectral power r^2) = 0.4749952 at sedimentation rate of 0.6652183 cm/ka

* Plotting/outputting time series calibrated to (envelope r^2) x (spectral power r^2) maximum

>

> # output optimal time series, bandpassed series, amplitude envelope and

> # TimeOpt-reconstructed eccentricity

> res2=timeOpt(dat1,sedmin=.29,sedmax=0.80,numsed=100,targetP=targetP,flow=1/19.8-0.01,fhigh=1/16.4+0.01,roll=10^7,limit=T,output=2);

----- TimeOpt: Assessment of Amplitude Modulation & Bundling-----

* Number of data points in stratigraphic series: 176

* Stratigraphic series length (meters): 3.5

* Sampling interval (meters): 0.02

* Linear trend subtracted. m= -0.002182244 b= 3.313136

* Using default eccentricity target periods (ka)= 405.6795 130.719 123.839 98.86307 94.87666

* PLEASE WAIT: Performing Optimization

0% 25% 50% 75% 100%

===========================================

* Maximum (spectral power r^2)= 0.5989703 at sedimentation rate of 0.7220639 cm/ka

* Maximum (envelope r^2)= 0.8253843 at sedimentation rate of 0.6517204 cm/ka

* Maximum (envelope r^2) x (spectral power r^2) = 0.4749952 at sedimentation rate of 0.6652183 cm/ka

* Plotting/outputting time series calibrated to (envelope r^2) x (spectral power r^2) maximum

>

> # (6) perform nominal timeOpt significance testing (this function call uses

> # (this function call uses 9 cores for parallel processing)

> simres=timeOptSim(dat1,sedmin=.29,sedmax=0.80,numsed=100,targetP=targetP,flow=1/19.8-0.01,fhigh=1/16.4+0.01,roll=10^7,numsim=2000,output=2,ncores=9);

----- TimeOpt Monte Carlo Simulation -----

* Number of data points in stratigraphic series: 176

* Stratigraphic series length (meters): 3.5

* Sampling interval (meters): 0.02

* Linear trend subtracted. m= -0.002182244 b= 3.313136

* Raw AR1 = 0.7495227

* (Envelope r^2) x (Spectral Power r^2) = 0.4749952

* PLEASE WAIT: Performing 2000 simulations using 9 cores

* (Envelope r^2) * (Spectral Power r^2) p-value = 0.005

> # (7) plot summary figure

> timeOptPlot(dat1,res1,res2,simres,flow=1/19.8-0.01,fhigh=1/16.4+0.01,roll=10^7,targetE=c(405,132,125,95),targetP=targetP,xlab="Height(m)",ylab="MS (×10-3 SI)",fitR=0.4749952,verbose=T);

----- Generating summary plot for TimeOpt analysis----

**** WARNING: using default height and width of 7 inches.

>

**1.8 The Subset D2-1 in the ZK68 drillcore**

> # (1) Load the package Astrochron

> library(astrochron)

>

> # (2) Obtained the MS data of Subset D2-1 (1471.99-1458.38 m) in the ZK68 drillcore

> dat <- read()

----- READ STRATIGRAPHIC SERIES FROM DATA FILE -----

The following options are selected:

* What type of column delimiter are you using?: Comma

* Does your file have column titles/headers?: auto detect

<PLEASE CHOOSE YOUR FILE>

* No column titles/headers detected

* Number of stratigraphic samples (rows)= 682

* Number of variables (columns)= 1 (excluding depth/height/time)

* Sorting data into increasing depth/height/time order.

Will remove empty entries (from either column).

* Number of samples (rows) post-sorting= 682

* Mean sampling interval= 0.02

* Median sampling interval= 0.02

* Maximum sampling interval= 0.02

* Minimum sampling interval= 0.02

* First 3 lines of data file:

V1 V2

1 1458.38 0.0407

2 1458.40 0.0453

3 1458.42 0.0480

>

> # (3) Isolate data of interest, between 1460.1 and 1462.4 m

> dat1=iso(dat,xmin=1460.1,xmax=1462.4);

----- ISOLATE STRATIGRAPHIC DATA BY LOCATION -----

* Number of data points= 682

* Number of columns= 2

* Minimum= 1458.38 , Maximum= 1472

* Isolating data between 1460.1 and 1462.4

* Number of data points following culling= 116

>

> # (4) Define the target periods from Laskar (2004)

> targetP=c(132,124,99,95,90)

>

> # (5) run nominal timeOpt

> # output sedimentation rate grid and fit

> res1=timeOpt(dat1,sedmin=0.065,sedmax=0.18,numsed=200,targetP=targetP,fitModPwr=TRUE,flow=1/132-0.005,fhigh=1/90+0.005,roll=10^7,limit=T,output=1);

----- TimeOpt: Assessment of Amplitude Modulation & Bundling-----

* Number of data points in stratigraphic series: 116

* Stratigraphic series length (meters): 2.3

* Sampling interval (meters): 0.02

* Linear trend subtracted. m= 0.001403068 b= -1.998286

* Using default eccentricity target periods (ka)= 405.6795 130.719 123.839 98.86307 94.87666

* PLEASE WAIT: Performing Optimization

0% 25% 50% 75% 100%

===========================================

* Maximum (spectral power r^2)= 0.5941429 at sedimentation rate of 0.1641573 cm/ka

* Maximum (envelope r^2)= 0.361623 at sedimentation rate of 0.1600094 cm/ka

* Maximum (envelope r^2) x (spectral power r^2) = 0.2042748 at sedimentation rate of 0.1616558 cm/ka

* Plotting/outputting time series calibrated to (envelope r^2) x (spectral power r^2) maximum

>

> # output optimal time series, bandpassed series, amplitude envelope and

> # TimeOpt-reconstructed eccentricity

> res2=timeOpt(dat1,sedmin=0.065,sedmax=0.18,numsed=200,targetP=targetP,fitModPwr=TRUE, flow=1/132-0.005,fhigh=1/90+0.005,roll=10^7,limit=T,output=2);

----- TimeOpt: Assessment of Amplitude Modulation & Bundling-----

* Number of data points in stratigraphic series: 116

* Stratigraphic series length (meters): 2.3

* Sampling interval (meters): 0.02

* Linear trend subtracted. m= 0.001403068 b= -1.998286

* Using default eccentricity target periods (ka)= 405.6795 130.719 123.839 98.86307 94.87666

* PLEASE WAIT: Performing Optimization

0% 25% 50% 75% 100%

===========================================

* Maximum (spectral power r^2)= 0.5941429 at sedimentation rate of 0.1641573 cm/ka

* Maximum (envelope r^2)= 0.361623 at sedimentation rate of 0.1600094 cm/ka

* Maximum (envelope r^2) x (spectral power r^2) = 0.2042748 at sedimentation rate of 0.1616558 cm/ka

* Plotting/outputting time series calibrated to (envelope r^2) x (spectral power r^2) maximum

>

> # (6) perform nominal timeOpt significance testing (this function call uses

> # (this function call uses 9 cores for parallel processing)

> simres=timeOptSim(dat1, sedmin=0.065,sedmax=0.18,numsed=200,targetP=targetP,fitModPwr=TRUE, flow=1/132-0.005,fhigh=1/90+0.005,roll=10^7,limit=T,numsim=2000,output=2,ncores=9);

----- TimeOpt Monte Carlo Simulation -----

* Number of data points in stratigraphic series: 116

* Stratigraphic series length (meters): 2.3

* Sampling interval (meters): 0.02

* Linear trend subtracted. m= 0.001403068 b= -1.998286

* Raw AR1 = 0.6931489

* (Envelope r^2) x (Spectral Power r^2) = 0.2042748

* PLEASE WAIT: Performing 2000 simulations using 9 cores

* (Envelope r^2) * (Spectral Power r^2) p-value = 0.0235

> # (7) plot summary figure

> timeOptPlot(dat1,res1,res2,simres, flow=1/132-0.005,fhigh=1/90+0.005,roll=10^7,targetE=c(405),targetP=targetP,xlab="Height(m)",ylab="MS (×10-3 SI)",fitR=0.2042748,verbose=T);

----- Generating summary plot for TimeOpt analysis----

**** WARNING: using default height and width of 7 inches.

>

**1.9 The Subset D2-2 in the ZK68 drillcore**

> # (1) Load the package Astrochron

> library(astrochron)

>

> # (2) Obtained the MS data of Subset D2-2 (1458.38-1445.72m) in the ZK68 drillcore

> dat <- read()

----- READ STRATIGRAPHIC SERIES FROM DATA FILE -----

The following options are selected:

* What type of column delimiter are you using?: Comma

* Does your file have column titles/headers?: auto detect

<PLEASE CHOOSE YOUR FILE>

* No column titles/headers detected

* Number of stratigraphic samples (rows)= 634

* Number of variables (columns)= 1 (excluding depth/height/time)

* Sorting data into increasing depth/height/time order.

Will remove empty entries (from either column).

* Number of samples (rows) post-sorting= 634

* Mean sampling interval= 0.02

* Median sampling interval= 0.02

* Maximum sampling interval= 0.02

* Minimum sampling interval= 0.02

* First 3 lines of data file:

V1 V2

1 1445.72 0.158

2 1445.74 0.144

3 1445.76 0.145

>

> # (3) Isolate data of interest, between 1448.8 and 1450.65 m

> dat1=iso(dat,xmin=1448.8,xmax=1450.65);

----- ISOLATE STRATIGRAPHIC DATA BY LOCATION -----

* Number of data points= 634

* Number of columns= 2

* Minimum= 1445.72 , Maximum= 1458.38

* Isolating data between 1448.8 and 1450.65

* Number of data points following culling= 93

>

> # (4) Define the target periods from Laskar (2004)

> targetP=c(132,124,99,95,90)

>

> # (5) run nominal timeOpt

> # output sedimentation rate grid and fit

> res1=timeOpt(dat1,sedmin=0.065,sedmax=0.19,numsed=200,targetP=targetP,fitModPwr=TRUE,flow=1/132-0.005,fhigh=1/90+0.005,roll=10^7,limit=T,output=1);

----- TimeOpt: Assessment of Amplitude Modulation & Bundling-----

* Number of data points in stratigraphic series: 93

* Stratigraphic series length (meters): 1.84

* Sampling interval (meters): 0.02

* Linear trend subtracted. m= 0.0007311032 b= -0.936395

* Using default eccentricity target periods (ka)= 405.6795 130.719 123.839 98.86307 94.87666

* PLEASE WAIT: Performing Optimization

0% 25% 50% 75% 100%

===========================================

* Maximum (spectral power r^2)= 0.6845105 at sedimentation rate of 0.1869523 cm/ka

* Maximum (envelope r^2)= 0.4250241 at sedimentation rate of 0.1790621 cm/ka

* Maximum (envelope r^2) x (spectral power r^2) = 0.2864368 at sedimentation rate of 0.1859473 cm/ka

* Plotting/outputting time series calibrated to (envelope r^2) x (spectral power r^2) maximum

> # output optimal time series, bandpassed series, amplitude envelope and

> # TimeOpt-reconstructed eccentricity

> res2=timeOpt(dat1,sedmin=0.065,sedmax=0.19,numsed=200,targetP=targetP,fitModPwr=TRUE, flow=1/132-0.005,fhigh=1/90+0.005,roll=10^7,limit=T,output=2);

----- TimeOpt: Assessment of Amplitude Modulation & Bundling-----

* Number of data points in stratigraphic series: 93

* Stratigraphic series length (meters): 1.84

* Sampling interval (meters): 0.02

* Linear trend subtracted. m= 0.0007311032 b= -0.936395

* Using default eccentricity target periods (ka)= 405.6795 130.719 123.839 98.86307 94.87666

* PLEASE WAIT: Performing Optimization

0% 25% 50% 75% 100%

===========================================

* Maximum (spectral power r^2)= 0.6845105 at sedimentation rate of 0.1869523 cm/ka

* Maximum (envelope r^2)= 0.4250241 at sedimentation rate of 0.1790621 cm/ka

* Maximum (envelope r^2) x (spectral power r^2) = 0.2864368 at sedimentation rate of 0.1859473 cm/ka

* Plotting/outputting time series calibrated to (envelope r^2) x (spectral power r^2) maximum

>

> # (6) perform nominal timeOpt significance testing (this function call uses

> # (this function call uses 9 cores for parallel processing)

> simres=timeOptSim(dat1, sedmin=0.065,sedmax=0.19,numsed=200,targetP=targetP,fitModPwr=TRUE, flow=1/132-0.005,fhigh=1/90+0.005,roll=10^7,limit=T,numsim=2000,output=2,ncores=9);

----- TimeOpt Monte Carlo Simulation -----

* Number of data points in stratigraphic series: 93

* Stratigraphic series length (meters): 1.84

* Sampling interval (meters): 0.02

* Linear trend subtracted. m= 0.0007311032 b= -0.936395

* Raw AR1 = 0.8132879

* (Envelope r^2) x (Spectral Power r^2) = 0.2864368

* PLEASE WAIT: Performing 2000 simulations using 9 cores

* (Envelope r^2) * (Spectral Power r^2) p-value = 0.078

> # (7) plot summary figure

> timeOptPlot(dat1,res1,res2,simres, flow=1/132-0.005,fhigh=1/90+0.005,roll=10^7,targetE=c(405),targetP=targetP,xlab="Height(m)",ylab="MS (×10-3 SI)",fitR=0.2864368,verbose=T);

----- Generating summary plot for TimeOpt analysis----

**** WARNING: using default height and width of 7 inches.

>

**1.10 The Subset D2-3 in the ZK68 drillcore**

> # (1) Load the package Astrochron

> library(astrochron)

>

> # (2) Obtained the MS data of Subset D2-3 (1445.72-1430 m) in the ZK68 drillcore

> dat <- read()

----- READ STRATIGRAPHIC SERIES FROM DATA FILE -----

The following options are selected:

* What type of column delimiter are you using?: Comma

* Does your file have column titles/headers?: auto detect

<PLEASE CHOOSE YOUR FILE>

* No column titles/headers detected

* Number of stratigraphic samples (rows)= 787

* Number of variables (columns)= 1 (excluding depth/height/time)

* Sorting data into increasing depth/height/time order.

Will remove empty entries (from either column).

* Number of samples (rows) post-sorting= 787

* Mean sampling interval= 0.02

* Median sampling interval= 0.02

* Maximum sampling interval= 0.02

* Minimum sampling interval= 0.02

* First 3 lines of data file:

V1 V2

1 1430.00 0.124

2 1430.02 0.132

3 1430.04 0.134

> # (3) Isolate data of interest, between 1443.2 and 1445.1 m

> dat1=iso(dat,xmin=1443.2,xmax=1445.1);

----- ISOLATE STRATIGRAPHIC DATA BY LOCATION -----

* Number of data points= 787

* Number of columns= 2

* Minimum= 1430 , Maximum= 1445.72

* Isolating data between 1443.2 and 1445.1

* Number of data points following culling= 96

>

> # (4) Define the target periods from Laskar (2004)

> targetP=c(132,124,99,95,90)

>

> # (5) run nominal timeOpt

> # output sedimentation rate grid and fit

> res1=timeOpt(dat1,sedmin=0.065,sedmax=0.12,numsed=200,targetP=targetP,fitModPwr=TRUE,flow=1/132-0.005,fhigh=1/90+0.005,roll=10^7,limit=T,output=1);

----- TimeOpt: Assessment of Amplitude Modulation & Bundling-----

* Number of data points in stratigraphic series: 96

* Stratigraphic series length (meters): 1.9

* Sampling interval (meters): 0.02

* Linear trend subtracted. m= -0.005074471 b= 7.497483

* Using default eccentricity target periods (ka)= 405.6795 130.719 123.839 98.86307 94.87666

* PLEASE WAIT: Performing Optimization

0% 25% 50% 75% 100%

===========================================

* Maximum (spectral power r^2)= 0.3496338 at sedimentation rate of 0.1044648 cm/ka

* Maximum (envelope r^2)= 0.3647931 at sedimentation rate of 0.1012954 cm/ka

* Maximum (envelope r^2) x (spectral power r^2) = 0.1203902 at sedimentation rate of 0.1025514 cm/ka

* Plotting/outputting time series calibrated to (envelope r^2) x (spectral power r^2) maximum

>

> # output optimal time series, bandpassed series, amplitude envelope and

> # TimeOpt-reconstructed eccentricity

> res2=timeOpt(dat1,sedmin=0.065,sedmax=0.12,numsed=200,targetP=targetP,fitModPwr=TRUE, flow=1/132-0.005,fhigh=1/90+0.005,roll=10^7,limit=T,output=2);

----- TimeOpt: Assessment of Amplitude Modulation & Bundling-----

* Number of data points in stratigraphic series: 96

* Stratigraphic series length (meters): 1.9

* Sampling interval (meters): 0.02

* Linear trend subtracted. m= -0.005074471 b= 7.497483

* Using default eccentricity target periods (ka)= 405.6795 130.719 123.839 98.86307 94.87666

* PLEASE WAIT: Performing Optimization

0% 25% 50% 75% 100%

===========================================

* Maximum (spectral power r^2)= 0.3496338 at sedimentation rate of 0.1044648 cm/ka

* Maximum (envelope r^2)= 0.3647931 at sedimentation rate of 0.1012954 cm/ka

* Maximum (envelope r^2) x (spectral power r^2) = 0.1203902 at sedimentation rate of 0.1025514 cm/ka

* Plotting/outputting time series calibrated to (envelope r^2) x (spectral power r^2) maximum

>

> # (6) perform nominal timeOpt significance testing (this function call uses

> # (this function call uses 9 cores for parallel processing)

> simres=timeOptSim(dat1, sedmin=0.065,sedmax=0.12,numsed=200,targetP=targetP,fitModPwr=TRUE, flow=1/132-0.005,fhigh=1/90+0.005,roll=10^7,limit=T,numsim=2000,output=2,ncores=9);

----- TimeOpt Monte Carlo Simulation -----

* Number of data points in stratigraphic series: 96

* Stratigraphic series length (meters): 1.9

* Sampling interval (meters): 0.02

* Linear trend subtracted. m= -0.005074471 b= 7.497483

* Raw AR1 = 0.6037285

* (Envelope r^2) x (Spectral Power r^2) = 0.1203902

* PLEASE WAIT: Performing 2000 simulations using 9 cores

* (Envelope r^2) * (Spectral Power r^2) p-value = 0.064

>

> # (7) plot summary figure

> timeOptPlot(dat1,res1,res2,simres, flow=1/132-0.005,fhigh=1/90+0.005,roll=10^7,targetE=c(405),targetP=targetP,xlab="Height(m)",ylab="MS (×10-3 SI)",fitR=0.1203902,verbose=T);

----- Generating summary plot for TimeOpt analysis----

**** WARNING: using default height and width of 7 inches.

>

**1.11 The Subset D2-4 in the ZK68 drillcore**

> # (1) Load the package Astrochron

> library(astrochron)

>

> # (2) Obtained the MS data of Subset D2-4 (1430-1413.5 m) in the ZK68 drillcore

> dat <- read()

----- READ STRATIGRAPHIC SERIES FROM DATA FILE -----

The following options are selected:

* What type of column delimiter are you using?: Comma

* Does your file have column titles/headers?: auto detect

<PLEASE CHOOSE YOUR FILE>

* No column titles/headers detected

* Number of stratigraphic samples (rows)= 827

* Number of variables (columns)= 1 (excluding depth/height/time)

* Sorting data into increasing depth/height/time order.

Will remove empty entries (from either column).

* Number of samples (rows) post-sorting= 827

* Duplicates found

* Duplicates values will be averaged.

* New number of samples (rows)= 826

* Mean sampling interval= 0.02

* Median sampling interval= 0.02

* Maximum sampling interval= 0.02

* Minimum sampling interval= 0.02

* First 3 lines of data file:

V1 V2

1 1413.50 0.131

2 1413.52 0.130

3 1413.54 0.131

>

> # (3) Isolate data of interest, between 1424 and 1425.8 m

> dat1=iso(dat,xmin=1424,xmax=1425.8);

----- ISOLATE STRATIGRAPHIC DATA BY LOCATION -----

* Number of data points= 826

* Number of columns= 2

* Minimum= 1413.5 , Maximum= 1430

* Isolating data between 1424 and 1425.8

* Number of data points following culling= 91

>

> # (4) Define the target periods from Laskar (2004)

> targetP=c(132,124,99,95,90)

>

> # (5) run nominal timeOpt

> # output sedimentation rate grid and fit

> res1=timeOpt(dat1,sedmin=0.065,sedmax=0.15,numsed=200,targetP=targetP,fitModPwr=TRUE,flow=1/132-0.005,fhigh=1/90+0.005,roll=10^7,limit=T,output=1);

----- TimeOpt: Assessment of Amplitude Modulation & Bundling-----

* Number of data points in stratigraphic series: 91

* Stratigraphic series length (meters): 1.8

* Sampling interval (meters): 0.02

* Linear trend subtracted. m= -0.007315416 b= 10.54634

* Using default eccentricity target periods (ka)= 405.6795 130.719 123.839 98.86307 94.87666

* PLEASE WAIT: Performing Optimization

0% 25% 50% 75% 100%

===========================================

* Maximum (spectral power r^2)= 0.4721349 at sedimentation rate of 0.1402462 cm/ka

* Maximum (envelope r^2)= 0.6032749 at sedimentation rate of 0.1384892 cm/ka

* Maximum (envelope r^2) x (spectral power r^2) = 0.282837 at sedimentation rate of 0.1396581 cm/ka

* Plotting/outputting time series calibrated to (envelope r^2) x (spectral power r^2) maximum

>

> # output optimal time series, bandpassed series, amplitude envelope and

> # TimeOpt-reconstructed eccentricity

> res2=timeOpt(dat1,sedmin=0.065,sedmax=0.15,numsed=200,targetP=targetP,fitModPwr=TRUE, flow=1/132-0.005,fhigh=1/90+0.005,roll=10^7,limit=T,output=2);

----- TimeOpt: Assessment of Amplitude Modulation & Bundling-----

* Number of data points in stratigraphic series: 91

* Stratigraphic series length (meters): 1.8

* Sampling interval (meters): 0.02

* Linear trend subtracted. m= -0.007315416 b= 10.54634

* Using default eccentricity target periods (ka)= 405.6795 130.719 123.839 98.86307 94.87666

* PLEASE WAIT: Performing Optimization

0% 25% 50% 75% 100%

===========================================

* Maximum (spectral power r^2)= 0.4721349 at sedimentation rate of 0.1402462 cm/ka

* Maximum (envelope r^2)= 0.6032749 at sedimentation rate of 0.1384892 cm/ka

* Maximum (envelope r^2) x (spectral power r^2) = 0.282837 at sedimentation rate of 0.1396581 cm/ka

* Plotting/outputting time series calibrated to (envelope r^2) x (spectral power r^2) maximum

>

> # (6) perform nominal timeOpt significance testing (this function call uses

> # (this function call uses 9 cores for parallel processing)

> simres=timeOptSim(dat1, sedmin=0.065,sedmax=0.15,numsed=200,targetP=targetP,fitModPwr=TRUE, flow=1/132-0.005,fhigh=1/90+0.005,roll=10^7,limit=T,numsim=2000,output=2,ncores=9);

----- TimeOpt Monte Carlo Simulation -----

* Number of data points in stratigraphic series: 91

* Stratigraphic series length (meters): 1.8

* Sampling interval (meters): 0.02

* Linear trend subtracted. m= -0.007315416 b= 10.54634

* Raw AR1 = 0.6787661

* (Envelope r^2) x (Spectral Power r^2) = 0.282837

* PLEASE WAIT: Performing 2000 simulations using 9 cores

* (Envelope r^2) * (Spectral Power r^2) p-value = 0.005

> # (7) plot summary figure

> timeOptPlot(dat1,res1,res2,simres, flow=1/132-0.005,fhigh=1/90+0.005,roll=10^7,targetE=c(405),targetP=targetP,xlab="Height(m)",ylab="MS (×10-3 SI)",fitR=0.282837,verbose=T);

----- Generating summary plot for TimeOpt analysis----

**** WARNING: using default height and width of 7 inches.

>

**1.12 The Subset D2-5 in the ZK68 drillcore**

> # (1) Load the package Astrochron

> library(astrochron)

>

> # (2) Obtained the MS data of Subset D2-5 (1413.5-1399.4 m) in the ZK68 drillcore

> dat <- read()

----- READ STRATIGRAPHIC SERIES FROM DATA FILE -----

The following options are selected:

* What type of column delimiter are you using?: Comma

* Does your file have column titles/headers?: auto detect

<PLEASE CHOOSE YOUR FILE>

* No column titles/headers detected

* Number of stratigraphic samples (rows)= 704

* Number of variables (columns)= 1 (excluding depth/height/time)

* Sorting data into increasing depth/height/time order.

Will remove empty entries (from either column).

* Number of samples (rows) post-sorting= 704

* Mean sampling interval= 0.02

* Median sampling interval= 0.02

* Maximum sampling interval= 0.02

* Minimum sampling interval= 0.02

* First 3 lines of data file:

V1 V2

1 1399.44 0.0330

2 1399.46 0.0300

3 1399.48 0.0535

>

> # (3) Isolate data of interest, between 1410 and 1411.9 m

> dat1=iso(dat,xmin=1410,xmax=1411.9);

----- ISOLATE STRATIGRAPHIC DATA BY LOCATION -----

* Number of data points= 704

* Number of columns= 2

* Minimum= 1399.44 , Maximum= 1413.5

* Isolating data between 1410 and 1411.9

* Number of data points following culling= 96

>

> # (4) Define the target periods from Laskar (2004)

> targetP=c(132,124,99,95,90)

>

> # (5) run nominal timeOpt

> # output sedimentation rate grid and fit

> res1=timeOpt(dat1,sedmin=0.088,sedmax=0.18,numsed=200,targetP=targetP,fitModPwr=TRUE,flow=1/132-0.005,fhigh=1/90+0.005,roll=10^7,limit=T,output=1);

----- TimeOpt: Assessment of Amplitude Modulation & Bundling-----

* Number of data points in stratigraphic series: 96

* Stratigraphic series length (meters): 1.9

* Sampling interval (meters): 0.02

* Linear trend subtracted. m= 0.01302587 b= -18.25891

* Using default eccentricity target periods (ka)= 405.6795 130.719 123.839 98.86307 94.87666

* PLEASE WAIT: Performing Optimization

0% 25% 50% 75% 100%

===========================================

* Maximum (spectral power r^2)= 0.5220214 at sedimentation rate of 0.1493004 cm/ka

* Maximum (envelope r^2)= 0.5405111 at sedimentation rate of 0.1693256 cm/ka

* Maximum (envelope r^2) x (spectral power r^2) = 0.2713258 at sedimentation rate of 0.1531064 cm/ka

* Plotting/outputting time series calibrated to (envelope r^2) x (spectral power r^2) maximum

>

> # output optimal time series, bandpassed series, amplitude envelope and

> # TimeOpt-reconstructed eccentricity

> res2=timeOpt(dat1,sedmin=0.088,sedmax=0.18,numsed=200,targetP=targetP,fitModPwr=TRUE, flow=1/132-0.005,fhigh=1/90+0.005,roll=10^7,limit=T,output=2);

----- TimeOpt: Assessment of Amplitude Modulation & Bundling-----

* Number of data points in stratigraphic series: 96

* Stratigraphic series length (meters): 1.9

* Sampling interval (meters): 0.02

* Linear trend subtracted. m= 0.01302587 b= -18.25891

* Using default eccentricity target periods (ka)= 405.6795 130.719 123.839 98.86307 94.87666

* PLEASE WAIT: Performing Optimization

0% 25% 50% 75% 100%

===========================================

* Maximum (spectral power r^2)= 0.5220214 at sedimentation rate of 0.1493004 cm/ka

* Maximum (envelope r^2)= 0.5405111 at sedimentation rate of 0.1693256 cm/ka

* Maximum (envelope r^2) x (spectral power r^2) = 0.2713258 at sedimentation rate of 0.1531064 cm/ka

* Plotting/outputting time series calibrated to (envelope r^2) x (spectral power r^2) maximum

>

> # (6) perform nominal timeOpt significance testing (this function call uses

> # (this function call uses 7 cores for parallel processing)

> simres=timeOptSim(dat1, sedmin=0.088,sedmax=0.18,numsed=200,targetP=targetP,fitModPwr=TRUE, flow=1/132-0.005,fhigh=1/90+0.005,roll=10^7,limit=T,numsim=2000,output=2,ncores=7);

----- TimeOpt Monte Carlo Simulation -----

* Number of data points in stratigraphic series: 96

* Stratigraphic series length (meters): 1.9

* Sampling interval (meters): 0.02

* Linear trend subtracted. m= 0.01302587 b= -18.25891

* Raw AR1 = 0.7578902

* (Envelope r^2) x (Spectral Power r^2) = 0.2713258

* PLEASE WAIT: Performing 2000 simulations using 7 cores

* (Envelope r^2) * (Spectral Power r^2) p-value = 0.041

> # (7) plot summary figure

> timeOptPlot(dat1,res1,res2,simres, flow=1/132-0.005,fhigh=1/90+0.005,roll=10^7,targetE=c(405),targetP=targetP,xlab="Height(m)",ylab="MS (×10-3 SI)",fitR= 0.2713258,verbose=T);

----- Generating summary plot for TimeOpt analysis----

**** WARNING: using default height and width of 7 inches.

>

**1.13 The Subset D3 in the ZK68 drillcore**

> # (1) Load the package Astrochron

> library(astrochron)

>

> # (2) Obtained the MS data of Subset D3 (1399.4-1332.28 m) in the ZK68 drillcore

> dat <- read()

----- READ STRATIGRAPHIC SERIES FROM DATA FILE -----

The following options are selected:

* What type of column delimiter are you using?: Comma

* Does your file have column titles/headers?: auto detect

<PLEASE CHOOSE YOUR FILE>

* No column titles/headers detected

* Number of stratigraphic samples (rows)= 773

* Number of variables (columns)= 1 (excluding depth/height/time)

* Sorting data into increasing depth/height/time order.

Will remove empty entries (from either column).

* Number of samples (rows) post-sorting= 773

* Mean sampling interval= 0.02

* Median sampling interval= 0.02

* Maximum sampling interval= 0.02

* Minimum sampling interval= 0.02

* First 3 lines of data file:

V1 V2

1 1384.00 0.171

2 1384.02 0.203

3 1384.04 0.227

>

> # (3) Isolate data of interest, between 1396 and 1399.3 m

> dat1=iso(dat,xmin=1396,xmax=1399.3);

----- ISOLATE STRATIGRAPHIC DATA BY LOCATION -----

* Number of data points= 773

* Number of columns= 2

* Minimum= 1384 , Maximum= 1399.44

* Isolating data between 1396 and 1399.3

* Number of data points following culling= 166

>

> # (4) Define the target periods from Waltham (2015)

> targetP=c(20.1,19.1,16.7,16.6)

>

> # (5) run nominal timeOpt

> # output sedimentation rate grid and fit

> res1=timeOpt(dat1,sedmin=.29,sedmax=0.60,numsed=100,targetP=targetP,flow=1/20.1-0.01,fhigh=1/16.6+0.01,roll=10^7,limit=T,output=1);

----- TimeOpt: Assessment of Amplitude Modulation & Bundling-----

* Number of data points in stratigraphic series: 166

* Stratigraphic series length (meters): 3.3

* Sampling interval (meters): 0.02

* Linear trend subtracted. m= -0.0008218809 b= 1.198917

* Using default eccentricity target periods (ka)= 405.6795 130.719 123.839 98.86307 94.87666

* PLEASE WAIT: Performing Optimization

0% 25% 50% 75% 100%

===========================================

* Maximum (spectral power r^2)= 0.3782907 at sedimentation rate of 0.5295793 cm/ka

* Maximum (envelope r^2)= 0.5785241 at sedimentation rate of 0.5295793 cm/ka

* Maximum (envelope r^2) x (spectral power r^2) = 0.2188503 at sedimentation rate of 0.5295793 cm/ka

* Plotting/outputting time series calibrated to (envelope r^2) x (spectral power r^2) maximum

>

> # output optimal time series, bandpassed series, amplitude envelope and

> # TimeOpt-reconstructed eccentricity

> res2=timeOpt(dat1,sedmin=.29,sedmax=0.60,numsed=100,targetP=targetP,flow=1/20.1-0.01,fhigh=1/16.6+0.01,roll=10^7,limit=T,output=2);

----- TimeOpt: Assessment of Amplitude Modulation & Bundling-----

* Number of data points in stratigraphic series: 166

* Stratigraphic series length (meters): 3.3

* Sampling interval (meters): 0.02

* Linear trend subtracted. m= -0.0008218809 b= 1.198917

* Using default eccentricity target periods (ka)= 405.6795 130.719 123.839 98.86307 94.87666

* PLEASE WAIT: Performing Optimization

0% 25% 50% 75% 100%

===========================================

* Maximum (spectral power r^2)= 0.3782907 at sedimentation rate of 0.5295793 cm/ka

* Maximum (envelope r^2)= 0.5785241 at sedimentation rate of 0.5295793 cm/ka

* Maximum (envelope r^2) x (spectral power r^2) = 0.2188503 at sedimentation rate of 0.5295793 cm/ka

* Plotting/outputting time series calibrated to (envelope r^2) x (spectral power r^2) maximum

>

> # (6) perform nominal timeOpt significance testing (this function call uses

> # (this function call uses 9 cores for parallel processing)

> simres=timeOptSim(dat1,sedmin=.29,sedmax=0.60,numsed=100,targetP=targetP,flow=1/20.1-0.01,fhigh=1/16.6+0.01,roll=10^7,numsim=2000,output=2,ncores=9);

----- TimeOpt Monte Carlo Simulation -----

* Number of data points in stratigraphic series: 166

* Stratigraphic series length (meters): 3.3

* Sampling interval (meters): 0.02

* Linear trend subtracted. m= -0.0008218809 b= 1.198917

* Raw AR1 = 0.4998404

* (Envelope r^2) x (Spectral Power r^2) = 0.2188503

* PLEASE WAIT: Performing 2000 simulations using 9 cores

* (Envelope r^2) * (Spectral Power r^2) p-value = 0.04

> # (7) plot summary figure

> timeOptPlot(dat1,res1,res2,simres,flow=1/20.1-0.01,fhigh=1/16.6+0.01,roll=10^7,targetE=c(405,125,95),targetP=targetP,xlab="Height(m)",ylab="MS (×10-3 SI)",fitR=0.2188503,verbose=T);

----- Generating summary plot for TimeOpt analysis----

**** WARNING: using default height and width of 7 inches.

>

**1.14 Supplementary TimeOpt analysis for the Subset D2-4 in the WD1 drillcore**

> # (1) Load the package Astrochron

> library(astrochron)

>

> # (2) Obtained the MS data of Subset D2-4 (2067.2-2088 m) in the WD1 drillcore

> dat <- read()

----- READ STRATIGRAPHIC SERIES FROM DATA FILE -----

The following options are selected:

* What type of column delimiter are you using?: Comma

* Does your file have column titles/headers?: auto detect

<PLEASE CHOOSE YOUR FILE>

* No column titles/headers detected

* Number of stratigraphic samples (rows)= 1038

* Number of variables (columns)= 1 (excluding depth/height/time)

* Sorting data into increasing depth/height/time order.

Will remove empty entries (from either column).

* Number of samples (rows) post-sorting= 1038

* Mean sampling interval= 0.02

* Median sampling interval= 0.02

* Maximum sampling interval= 0.02

* Minimum sampling interval= 0.02

* First 3 lines of data file:

V1 V2

1 2067.26 0.144

2 2067.28 0.171

3 2067.30 0.194

>

> # (3) Isolate data of interest, between 2070.3 and 2072.8 m

> dat1=iso(dat,xmin=2070.3,xmax=2072.8);

----- ISOLATE STRATIGRAPHIC DATA BY LOCATION -----

* Number of data points= 1038

* Number of columns= 2

* Minimum= 2067.26 , Maximum= 2088

* Isolating data between 2070.3 and 2072.8

* Number of data points following culling= 126

>

> # (4) Define the target periods from Waltham (2015)

> targetP=c(20.1,19.1,16.7,16.6)

>

> # (5) run nominal timeOpt

> # output sedimentation rate grid and fit

> res1=timeOpt(dat1,sedmin=.1,sedmax=0.8,numsed=100,targetP=targetP,flow=1/20.1-0.01,fhigh=1/16.6+0.01,roll=10^7,limit=T,output=1);

----- TimeOpt: Assessment of Amplitude Modulation & Bundling-----

* Number of data points in stratigraphic series: 126

* Stratigraphic series length (meters): 2.5

* Sampling interval (meters): 0.02

* Linear trend subtracted. m= -0.04326844 b= 89.85181

* Using default eccentricity target periods (ka)= 405.6795 130.719 123.839 98.86307 94.87666

**** WARNING: minimum sedimentation rate is too low for full signal recovery.

sedmin reset to 0.2809639 cm/ka

**** WARNING: maximum sedimentation rate is too high for full signal recovery.

sedmax reset to 0.62118 cm/ka

* PLEASE WAIT: Performing Optimization

0% 25% 50% 75% 100%

===========================================

* Maximum (spectral power r^2)= 0.4586824 at sedimentation rate of 0.371935 cm/ka

* Maximum (envelope r^2)= 0.7454977 at sedimentation rate of 0.5826043 cm/ka

* Maximum (envelope r^2) x (spectral power r^2) = 0.3210326 at sedimentation rate of 0.371935 cm/ka

* Plotting/outputting time series calibrated to (envelope r^2) x (spectral power r^2) maximum

>

> # output optimal time series, bandpassed series, amplitude envelope and

> # TimeOpt-reconstructed eccentricity

> res2=timeOpt(dat1,sedmin=.1,sedmax=0.8,numsed=100,targetP=targetP,flow=1/20.1-0.01,fhigh=1/16.6+0.01,roll=10^7,limit=T,output=2);

----- TimeOpt: Assessment of Amplitude Modulation & Bundling-----

* Number of data points in stratigraphic series: 126

* Stratigraphic series length (meters): 2.5

* Sampling interval (meters): 0.02

* Linear trend subtracted. m= -0.04326844 b= 89.85181

* Using default eccentricity target periods (ka)= 405.6795 130.719 123.839 98.86307 94.87666

**** WARNING: minimum sedimentation rate is too low for full signal recovery.

sedmin reset to 0.2809639 cm/ka

**** WARNING: maximum sedimentation rate is too high for full signal recovery.

sedmax reset to 0.62118 cm/ka

* PLEASE WAIT: Performing Optimization

0% 25% 50% 75% 100%

===========================================

* Maximum (spectral power r^2)= 0.4586824 at sedimentation rate of 0.371935 cm/ka

* Maximum (envelope r^2)= 0.7454977 at sedimentation rate of 0.5826043 cm/ka

* Maximum (envelope r^2) x (spectral power r^2) = 0.3210326 at sedimentation rate of 0.371935 cm/ka

* Plotting/outputting time series calibrated to (envelope r^2) x (spectral power r^2) maximum

>

> # (6) perform nominal timeOpt significance testing (this function call uses

> # (this function call uses 9 cores for parallel processing)

> simres=timeOptSim(dat1,sedmin=.1,sedmax=0.8,numsed=100,targetP=targetP,flow=1/20.1-0.01,fhigh=1/16.6+0.01,roll=10^7,numsim=2000,output=2,ncores=9);

----- TimeOpt Monte Carlo Simulation -----

* Number of data points in stratigraphic series: 126

* Stratigraphic series length (meters): 2.5

* Sampling interval (meters): 0.02

* Linear trend subtracted. m= -0.04326844 b= 89.85181

* Raw AR1 = 0.6199614

* (Envelope r^2) x (Spectral Power r^2) = 0.3210326

* PLEASE WAIT: Performing 2000 simulations using 9 cores

* (Envelope r^2) * (Spectral Power r^2) p-value = 0.2005

> # (7) plot summary figure

> timeOptPlot(dat1,res1,res2,simres,flow=1/20.1-0.01,fhigh=1/16.6+0.01,roll=10^7,targetE=c(405,125,95),targetP=targetP,xlab="Height(m)",ylab="MS (×10-3 SI)",fitR=0.3210686,verbose=T);

----- Generating summary plot for TimeOpt analysis----

**** WARNING: using default height and width of 7 inches.

>

**1.15 Supplementary TimeOpt analysis for the Subset D3 in the WD1 drillcore**

> # (1) Load the package Astrochron

> library(astrochron)

>

> # (2) Obtained the MS data of Subset D3 (2040-2067.2 m) in the WD1 drillcore

> dat <- read()

----- READ STRATIGRAPHIC SERIES FROM DATA FILE -----

The following options are selected:

* What type of column delimiter are you using?: Comma

* Does your file have column titles/headers?: auto detect

<PLEASE CHOOSE YOUR FILE>

* No column titles/headers detected

* Number of stratigraphic samples (rows)= 1364

* Number of variables (columns)= 1 (excluding depth/height/time)

* Sorting data into increasing depth/height/time order.

Will remove empty entries (from either column).

* Number of samples (rows) post-sorting= 1364

* Mean sampling interval= 0.02

* Median sampling interval= 0.02

* Maximum sampling interval= 0.02

* Minimum sampling interval= 0.02

* First 3 lines of data file:

V1 V2

1 2040.00 0.221

2 2040.02 0.279

3 2040.04 0.214

>

> # (3) Isolate data of interest, between 2051.2-2054.8 m

> dat1=iso(dat,xmin=2051.2,xmax=2054.8);

----- ISOLATE STRATIGRAPHIC DATA BY LOCATION -----

* Number of data points= 1364

* Number of columns= 2

* Minimum= 2040 , Maximum= 2067.26

* Isolating data between 2051.2 and 2054.8

* Number of data points following culling= 181

>

> # (4) Define the target periods from Waltham (2015)

> targetP=c(20.1,19.1,16.7,16.6)

>

> # (5) run nominal timeOpt

> # output sedimentation rate grid and fit

> res1=timeOpt(dat1,sedmin=.1,sedmax=1.0,numsed=100,targetP=targetP,flow=1/20.1-0.01,fhigh=1/16.6+0.01,roll=10^7,limit=T,output=1);

----- TimeOpt: Assessment of Amplitude Modulation & Bundling-----

* Number of data points in stratigraphic series: 181

* Stratigraphic series length (meters): 3.6

* Sampling interval (meters): 0.02

* Linear trend subtracted. m= -0.01286665 b= 26.5464

* Using default eccentricity target periods (ka)= 405.6795 130.719 123.839 98.86307 94.87666

**** WARNING: minimum sedimentation rate is too low for full signal recovery.

sedmin reset to 0.2809639 cm/ka

**** WARNING: maximum sedimentation rate is too high for full signal recovery.

sedmax reset to 0.89233 cm/ka

* PLEASE WAIT: Performing Optimization

0% 25% 50% 75% 100%

===========================================

* Maximum (spectral power r^2)= 0.6591493 at sedimentation rate of 0.89233 cm/ka

* Maximum (envelope r^2)= 0.7647042 at sedimentation rate of 0.8717393 cm/ka

* Maximum (envelope r^2) x (spectral power r^2) = 0.4895498 at sedimentation rate of 0.89233 cm/ka

* Plotting/outputting time series calibrated to (envelope r^2) x (spectral power r^2) maximum

>

> # output optimal time series, bandpassed series, amplitude envelope and

> # TimeOpt-reconstructed eccentricity

> res2=timeOpt(dat1,sedmin=.1,sedmax=1.0,numsed=100,targetP=targetP,flow=1/20.1-0.01,fhigh=1/16.6+0.01,roll=10^7,limit=T,output=2);

----- TimeOpt: Assessment of Amplitude Modulation & Bundling-----

* Number of data points in stratigraphic series: 181

* Stratigraphic series length (meters): 3.6

* Sampling interval (meters): 0.02

* Linear trend subtracted. m= -0.01286665 b= 26.5464

* Using default eccentricity target periods (ka)= 405.6795 130.719 123.839 98.86307 94.87666

**** WARNING: minimum sedimentation rate is too low for full signal recovery.

sedmin reset to 0.2809639 cm/ka

**** WARNING: maximum sedimentation rate is too high for full signal recovery.

sedmax reset to 0.89233 cm/ka

* PLEASE WAIT: Performing Optimization

0% 25% 50% 75% 100%

===========================================

* Maximum (spectral power r^2)= 0.6591493 at sedimentation rate of 0.89233 cm/ka

* Maximum (envelope r^2)= 0.7647042 at sedimentation rate of 0.8717393 cm/ka

* Maximum (envelope r^2) x (spectral power r^2) = 0.4895498 at sedimentation rate of 0.89233 cm/ka

* Plotting/outputting time series calibrated to (envelope r^2) x (spectral power r^2) maximum

>

> # (6) perform nominal timeOpt significance testing (this function call uses

> # (this function call uses 9 cores for parallel processing)

> simres=timeOptSim(dat1,sedmin=.1,sedmax=1.0,numsed=100,targetP=targetP,flow=1/20.1-0.01,fhigh=1/16.6+0.01,roll=10^7,numsim=2000,output=2,ncores=9);

----- TimeOpt Monte Carlo Simulation -----

* Number of data points in stratigraphic series: 181

* Stratigraphic series length (meters): 3.6

* Sampling interval (meters): 0.02

* Linear trend subtracted. m= -0.01286665 b= 26.5464

* Raw AR1 = 0.8065303

* (Envelope r^2) x (Spectral Power r^2) = 0.4895498

* PLEASE WAIT: Performing 2000 simulations using 9 cores

* (Envelope r^2) * (Spectral Power r^2) p-value = 0.0265

> # (7) plot summary figure

> timeOptPlot(dat1,res1,res2,simres,flow=1/20.1-0.01,fhigh=1/16.6+0.01,roll=10^7,targetE=c(405,125,95),targetP=targetP,xlab="Height(m)",ylab="MS (×10-3 SI)",fitR=0.4895498,verbose=T);

----- Generating summary plot for TimeOpt analysis----

**** WARNING: using default height and width of 7 inches.

>

**Supplementary Figures and Captions**

**
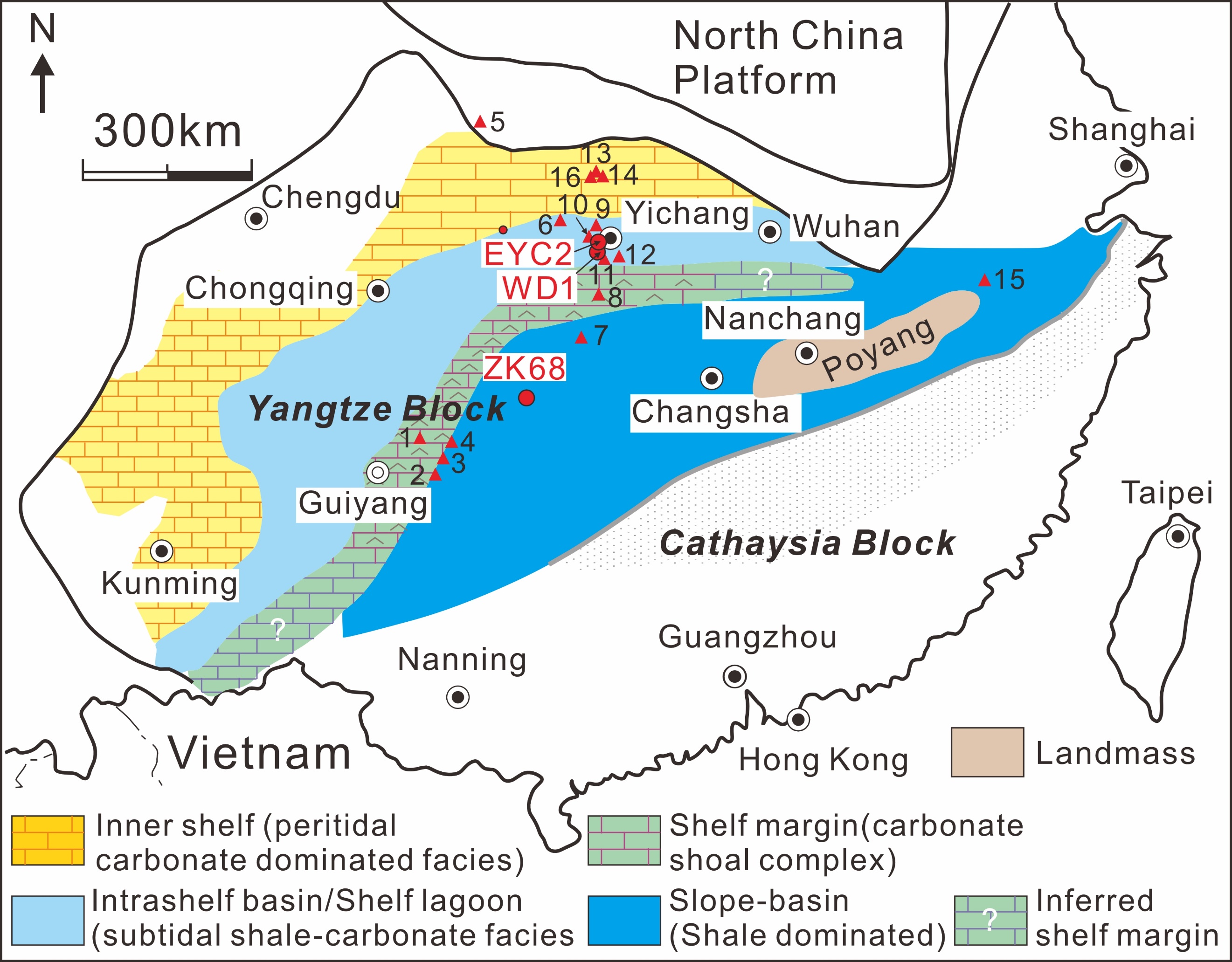
**

**Supplementary Fig. 1 Paleogeographic reconstruction of the Ediacaran Yangtze Platform (modified from refs.** 73**), showing locations of three studied drillcores (red circle) and relevant sections (red triangle).** Reprinted from [Gondwana Research, 19, Ganging Jiang, Xiaoying Shi, Shihong Zhang, Yue Wang, Shuhai Xiao, Stratigraphy and paleogeography of the Ediacaran Doushantuo Formation (ca. 635–551 Ma) in South China, 831-849, 2011], with permission from Elsevier. 1, Weng’an; 2, Nangao; 3, Wengxiu; 4, Xiajiaogmeng (Fanglong); 5, Xiaoyang; 6, Sixi, 7; Siduping; 8, Yangjiaping; 9, Jiulongwan; 10, Three Gorges Drillcore; 11, Wangjiapeng; 12, YD4 drillcore; 13, Zhangcunping, 14, ZK312-P312 drillcore; 15, Lantian; 16, Zhengjiatang. SC South China; NC North China.


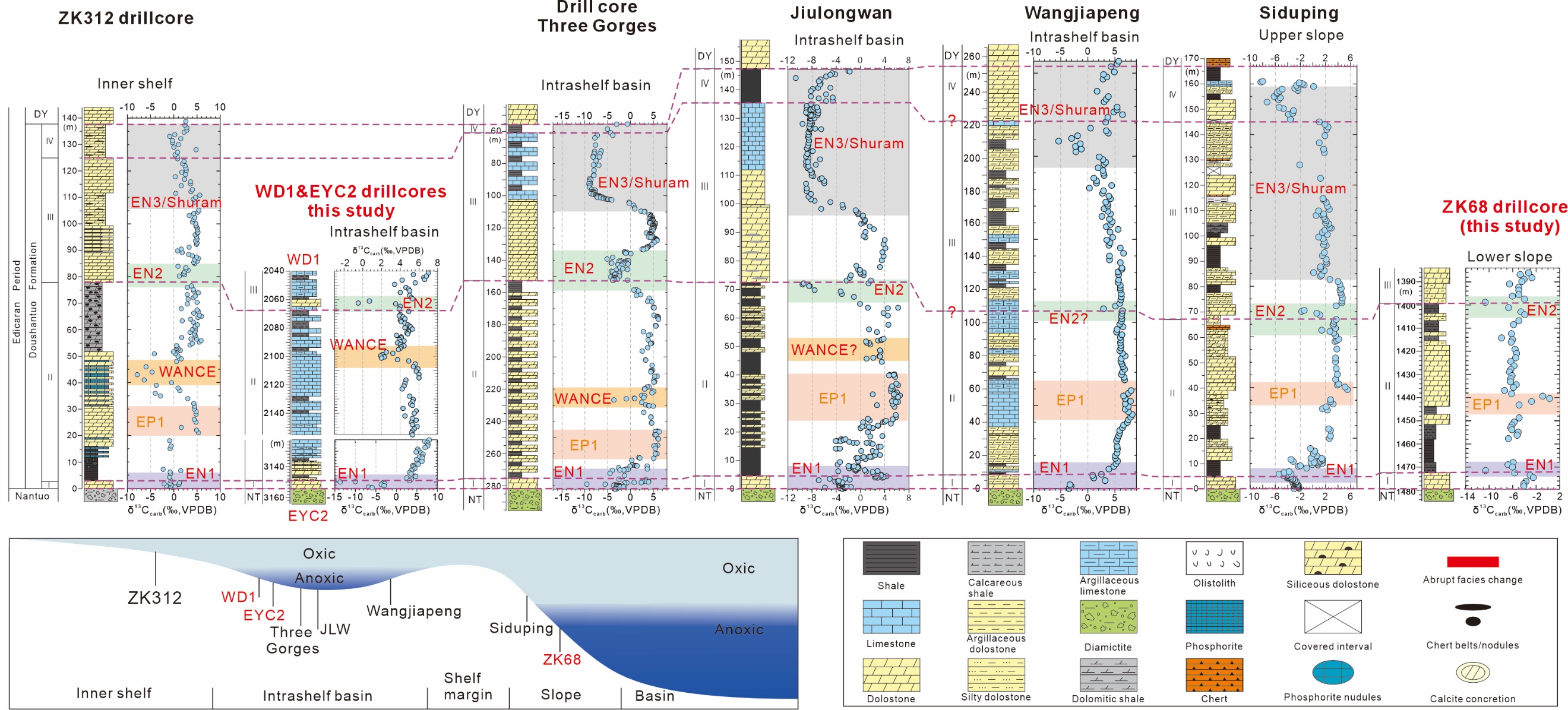


**Supplementary Fig. 2** **Correlation of δ^13^C_carb_ profiles of the Doushantuo Formation, across a shelf-to-basin transect of the Yangtze platform, South China.** Stratigraphic data sources: ZK312 drillcore^13^, WD1, EYC2 and ZK68 drillcores (this study), Three Gorges drillcore^83^, Jiulongwan section^6^, Wangjiapeng section^9^, Siduping section^13^. The dashed red line delineates the boundary between formations or members. The bottom left schematic diagram illustrates sedimentary facies variations of the Doushantuo Formation along a transect from northwest to southeast across the central Yangtze Platform (modified from ref. 12). Note that the distances between sections are not to scale.


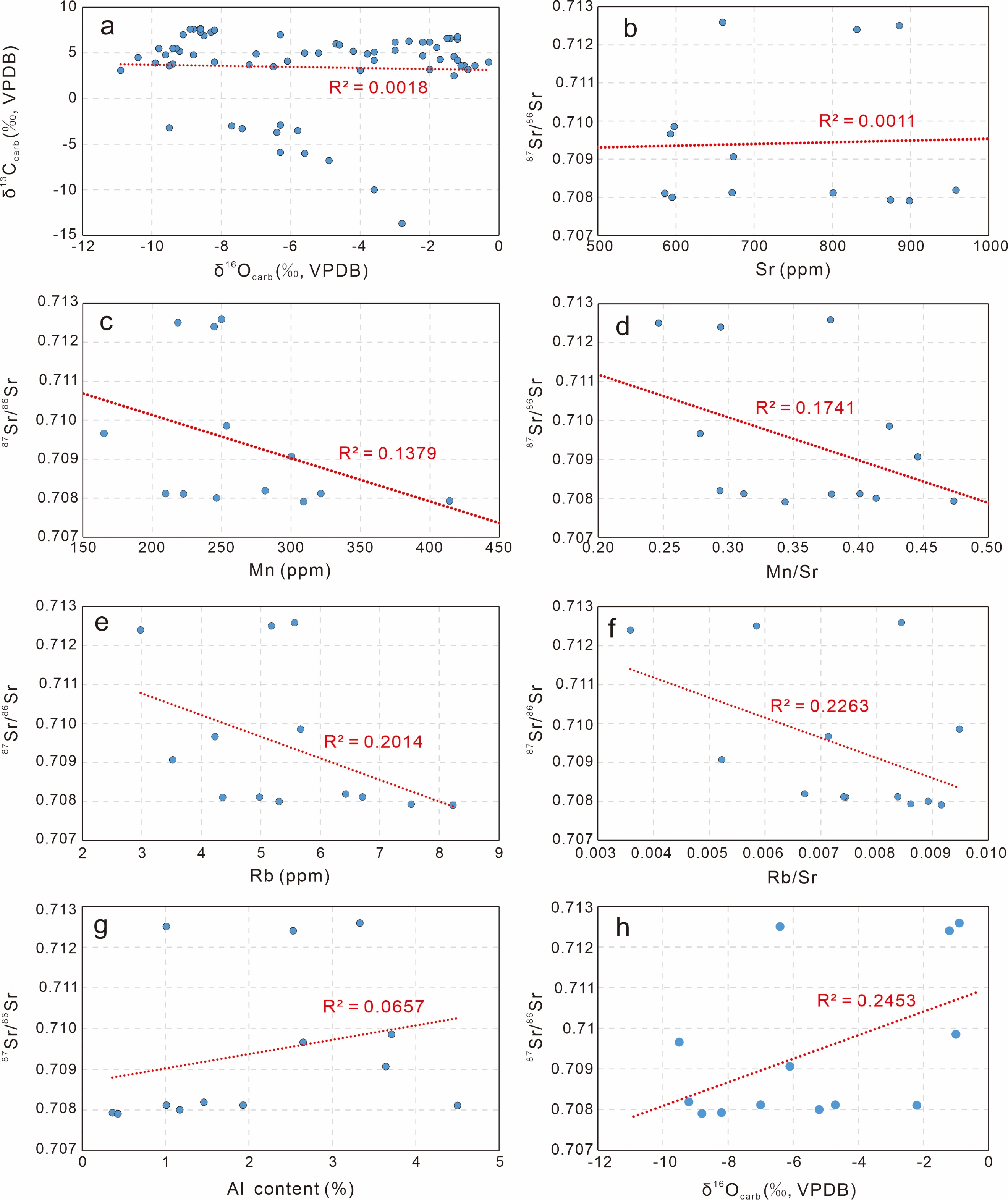


**Supplementary Fig. 3** **Cross-plots of the geochemical data from the EYC2 drillcore, showing linear regression lines (red dashed lines) and cross-correlation coefficients (R^2^)**. a. δ^13^C (‰)–δ^18^O (‰); b. ^87^Sr/^86^Sr–Sr (ppm); c. ^87^Sr/^86^Sr–Mn (ppm); d. ^87^Sr/^86^Sr–Mn/Sr ratios; e. ^87^Sr/^86^Sr–Rb (ppm); f. ^87^Sr/^86^Sr–Rb/Sr ratios; g. ^87^Sr/^86^Sr–Al (%); h. ^87^Sr/^86^Sr–δ^18^O_carb_ (‰).


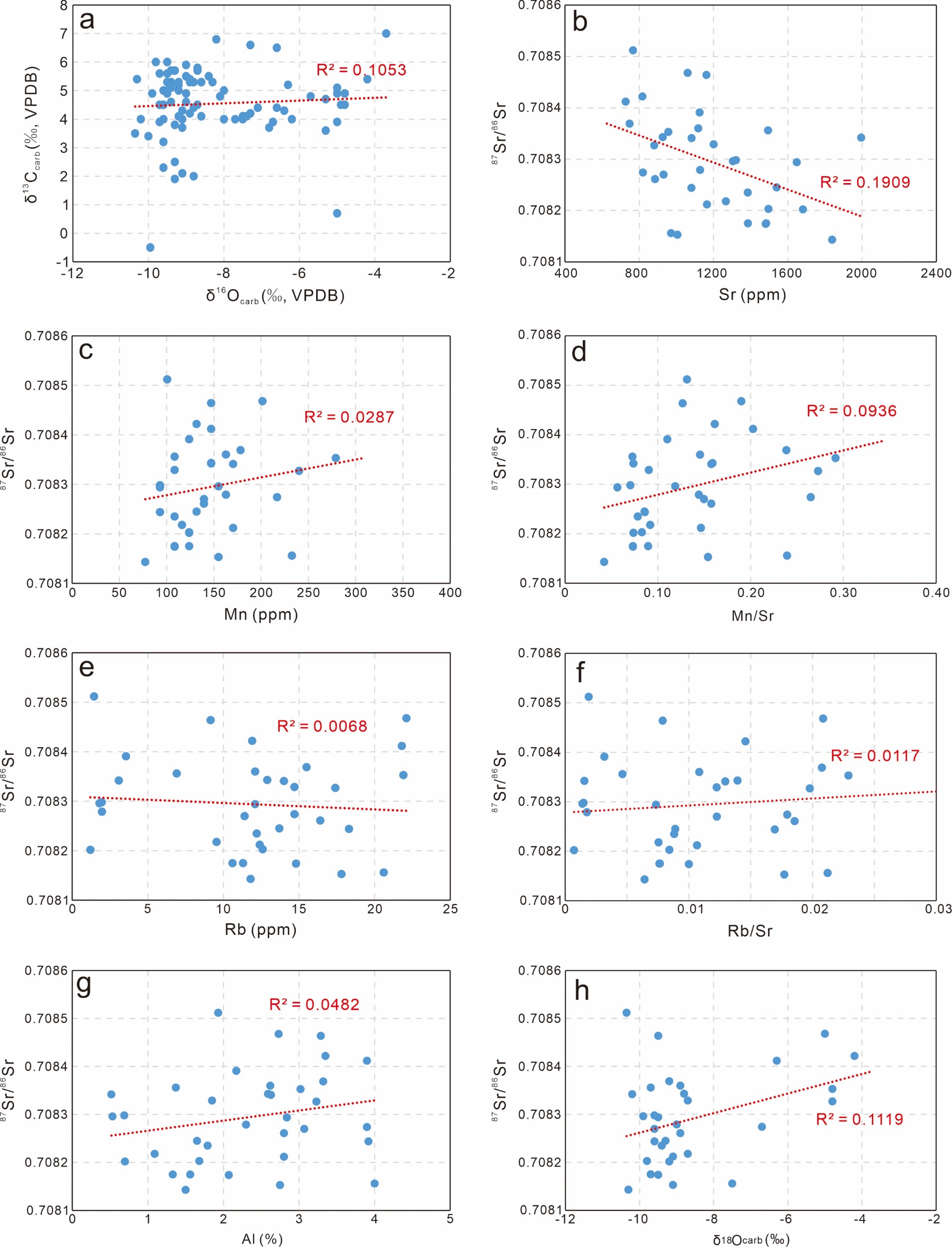
**Supplementary Fig. 4** **Cross-plots of the geochemical data from the WD1 drillcore, showing linear regression lines (red dashed lines) and cross-correlation coefficients (R^2^)**. **a**. δ^13^C (‰)–δ^18^O (‰); **b**. ^87^Sr/^86^Sr–Sr (ppm); **c**. ^87^Sr/^86^Sr–Mn (ppm); **d**. ^87^Sr/^86^Sr–Mn/Sr ratios; **e**. ^87^Sr/^86^Sr–Rb (ppm); **f**. ^87^Sr/^86^Sr–Rb/Sr ratios; **g**. ^87^Sr/^86^Sr–Al (%); **h**. ^87^Sr/^86^Sr–δ^18^O_carb_ (‰).


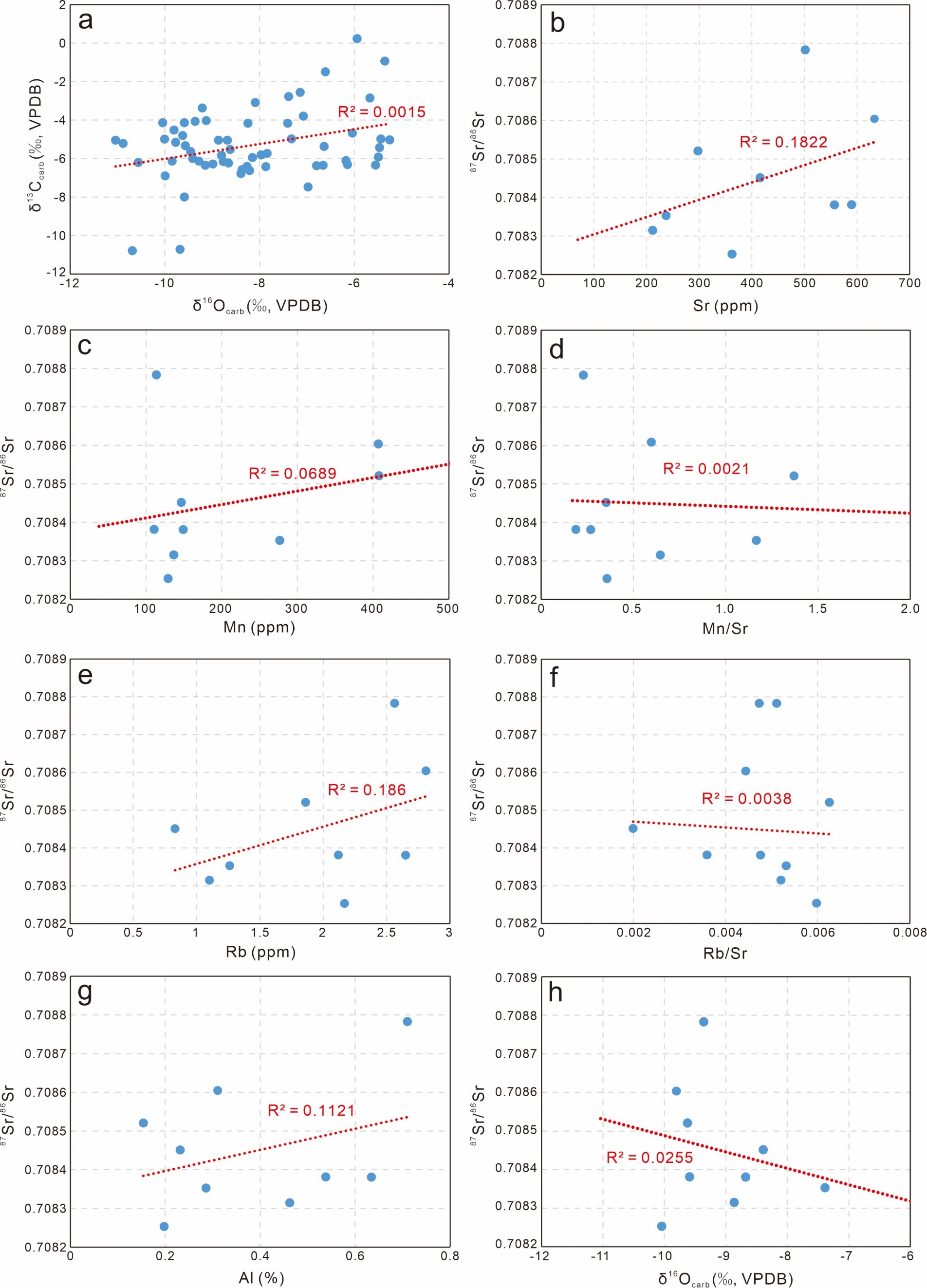


**Supplementary Fig. 5** **Cross-plots of the geochemical data from the ZK68 drillcore, showing linear regression lines (red dashed lines) and cross-correlation coefficients (R^2^)**. **a**. δ^13^C (‰)–δ^18^O (‰); b. ^87^Sr/^86^Sr–Sr (ppm); c. ^87^Sr/^86^Sr–Mn (ppm); d. ^87^Sr/^86^Sr–Mn/Sr ratios; e. ^87^Sr/^86^Sr–Rb (ppm); f. ^87^Sr/^86^Sr–Rb/Sr ratios; g. ^87^Sr/^86^Sr–Al (%); h. ^87^Sr/^86^Sr–δ^18^O_carb_ (‰).


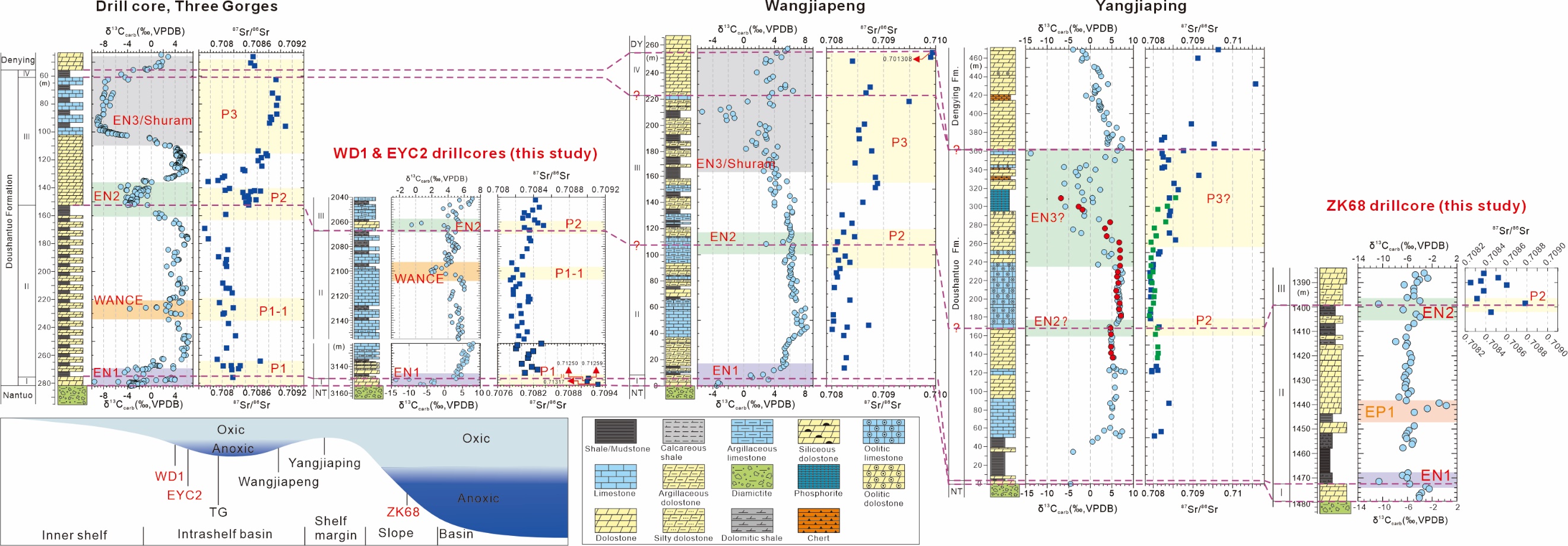


**Supplementary Fig. 6** **Correlation of ^87^Sr/^86^Sr profiles of the Doushantuo Formation, across a shelf-to-basin transect of the Yangtze platform, South China.** Data sources: Three Gorges drillcore^25^; WD1, EYC2 and ZK68 drillcores (this study); Wangjiapeng section^9^; Yangjiaping section: δ^13^C_carb_ data represented by light blue-filled symbols from ref. 84 and red-filled symbols from ref. 31, ^87^Sr/^86^Sr data represented by dark blue-filled symbols from ref. 32 and green-filled symbols from ref. 31.


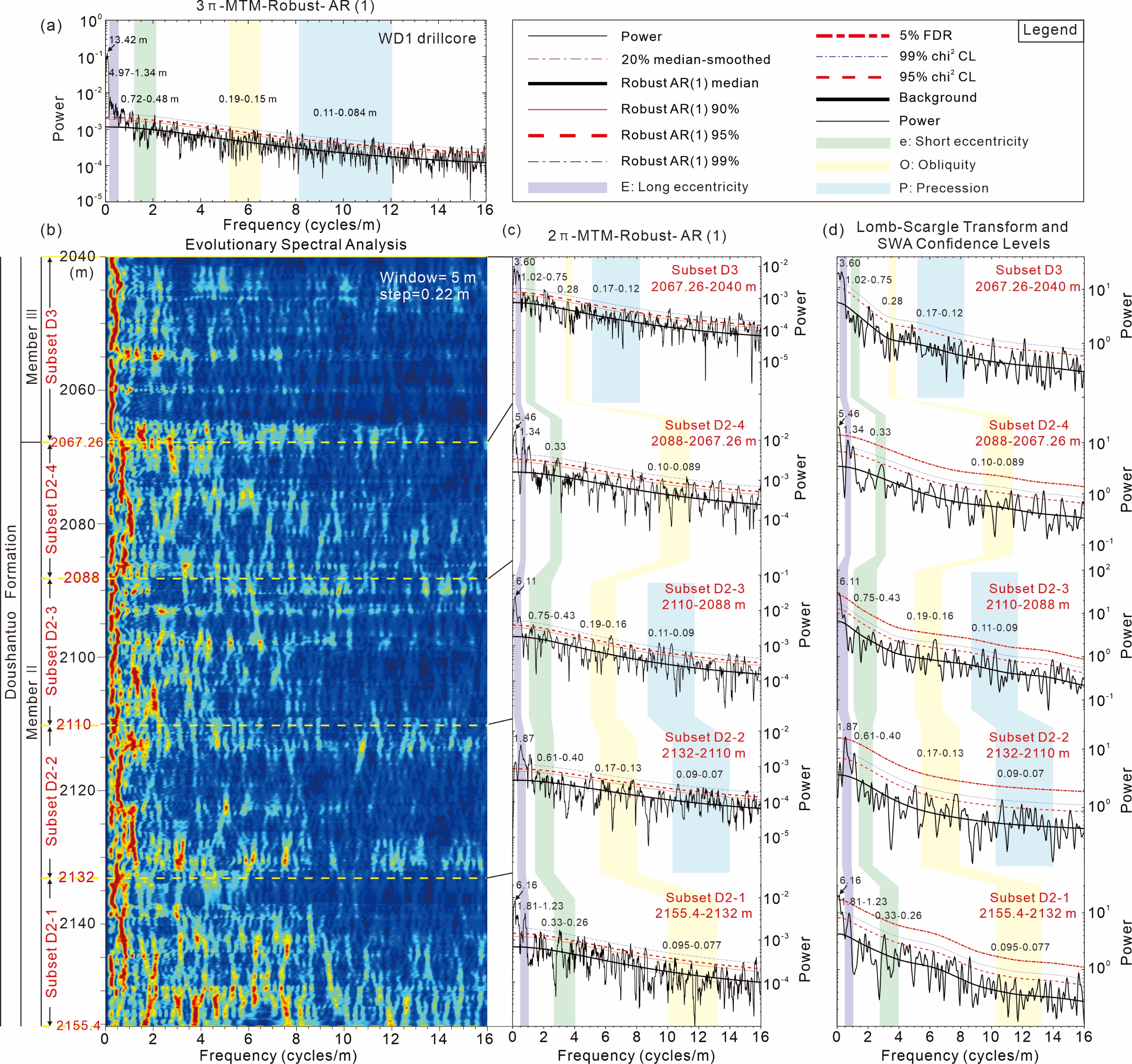


**Supplementary Fig. 7 Power spectra of the untuned MS series from the studied Doushantuo Formation in the WD1 drillcore.** **a** 3π MTM power spectrum of the entire untuned MS series after subtracting a 20% “LOESS” trend. **b** Evolutionary Faster Fourier transform (eFFT) spectrum of the detrended MS, with a 5 m sliding window and 0.22 m step length. **c** 2π MTM power spectrum of the untuned MS series for the five Subsets: 2155.4–2132 m, 2132–2110 m, 2110–2088 m, 2088–2067.26 m, and 2067.26-2040 m after subtracting “LOESS” trends of 46%, 46%, 25%, 53%, and 25%, respectively. **d** Lomb-Scargle spectra analysis of the untuned MS series for the five subsets using smoothed window averages (SWA) against the 5% false discovery rate (FDR). The significant peaks corresponding to long eccentricity, short eccentricity, obliquity, and precession cycles were identified and highlighted in each subset.


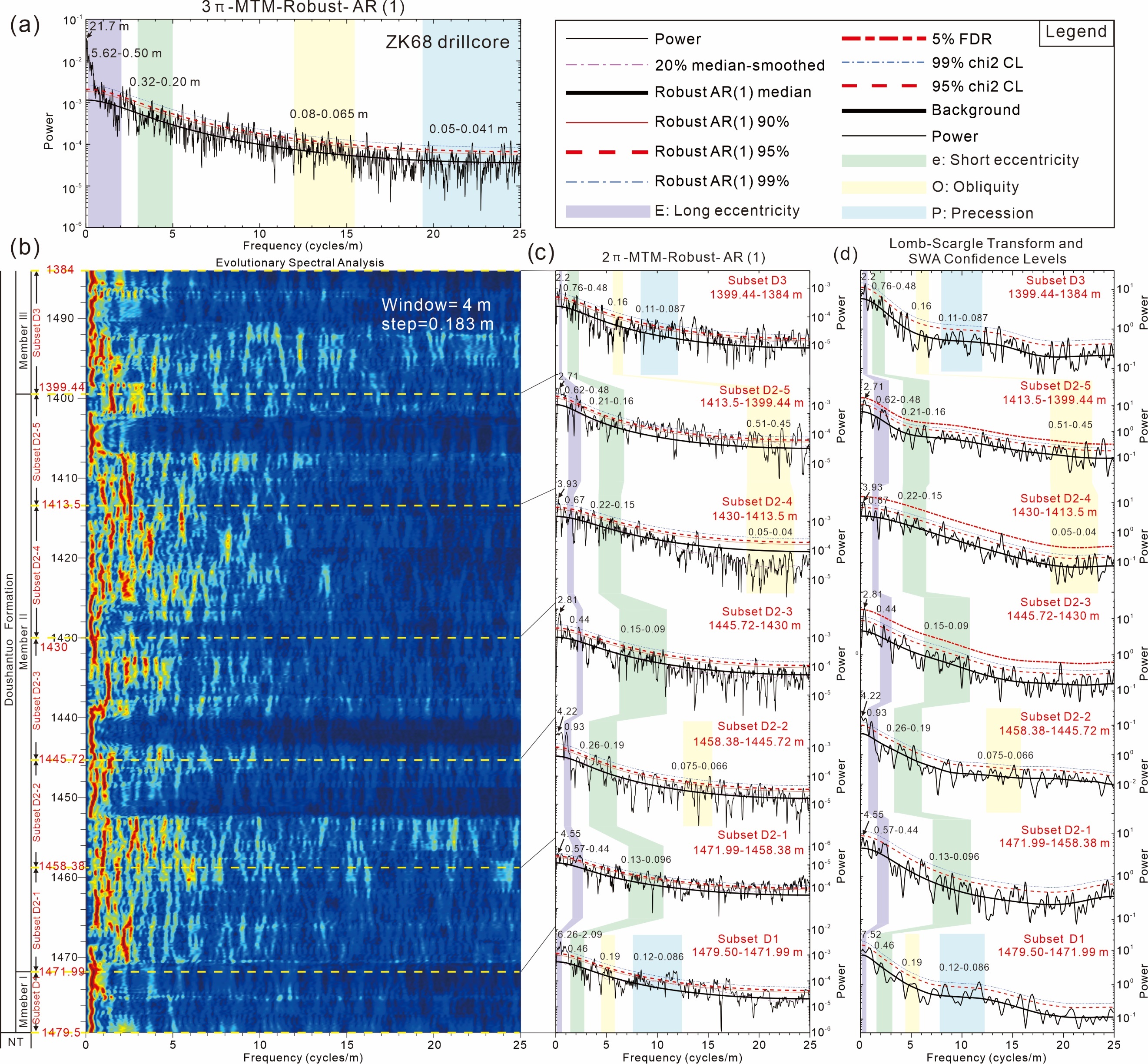


**Supplementary Fig. 8 Power spectra of the untuned MS series from the studied Doushantuo Formation in the ZK68 drillcore. a** 3π MTM power spectrum of the entire untuned MS series after subtracting a 20% “LOESS” trend. **b** Evolutionary Faster Fourier transform spectrum of the detrended MS, with a 4 m sliding window and 0.183 m step length. **c** 2π MTM power spectrum of the untuned MS series for the seven Subsets: 1479.5–1471.99 m, 1471.99–1458.38 m, 1458.38–1445.72 m, 1445.72–1430 m, 1430-1413.5 m, 1413.5-1399.44 m, and 1399.44–1384 m after subtracting “LOESS” trends of 51%, 75%, 58%, 20%, 39%, 28%, and 25%, respectively. **d** Lomb-Scargle spectra analysis of the untuned MS series for the seven subsets using smoothed window averages (SWA) against the 5% false discovery rate (FDR). The significant peaks corresponding to long eccentricity, short eccentricity, obliquity, and precession cycles were identified and highlighted in each subset. NT Nantuo Formation.


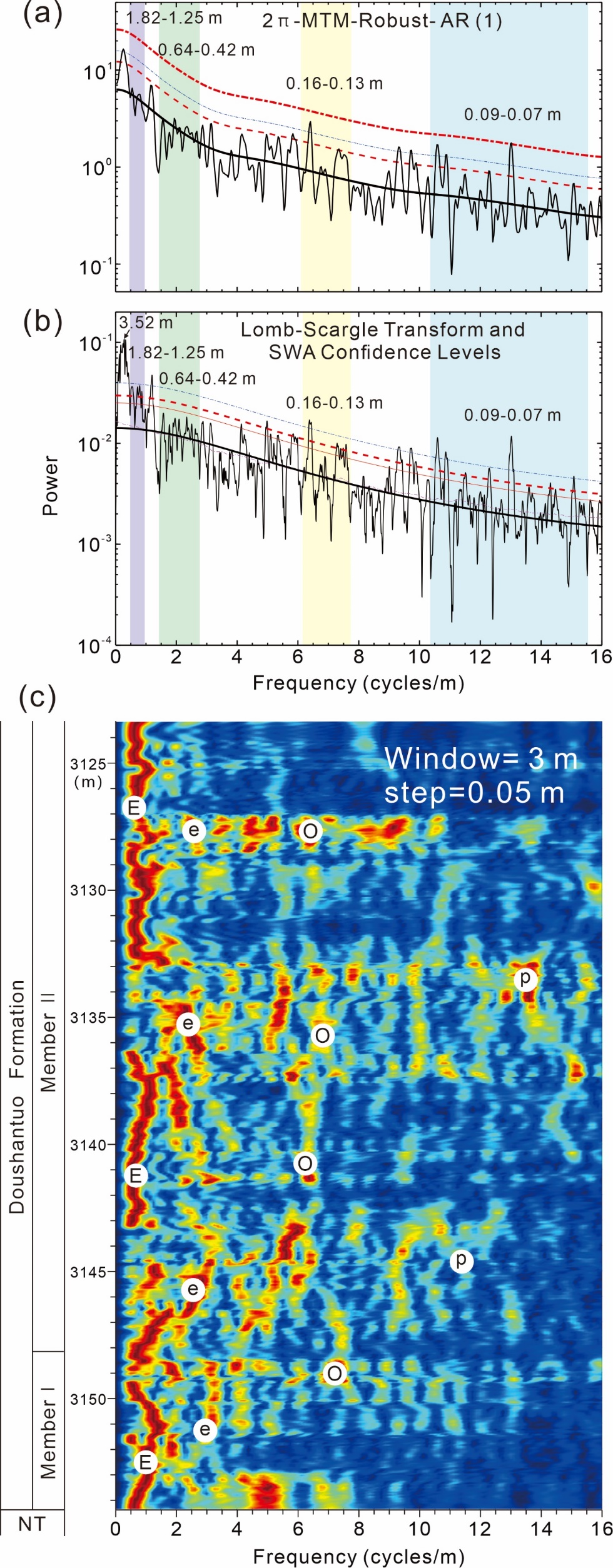


**Supplementary Fig. 9 Power spectra of the untuned MS series from the studied Doushantuo Formation in the EYC2 drillcore. a** 2π MTM power spectrum of the entire untuned MS series after subtracting a 21% locally estimated scatterplot smoothing (LOESS) trend. **b** Lomb-Scargle spectra analysis of the untuned MS series using smoothed window averages (SWA) against the 5% false discovery rate (FDR). **c** Evolutionary Faster Fourier transform spectrum of the detrended MS, with a 3 m sliding window and 0.05 m step length.


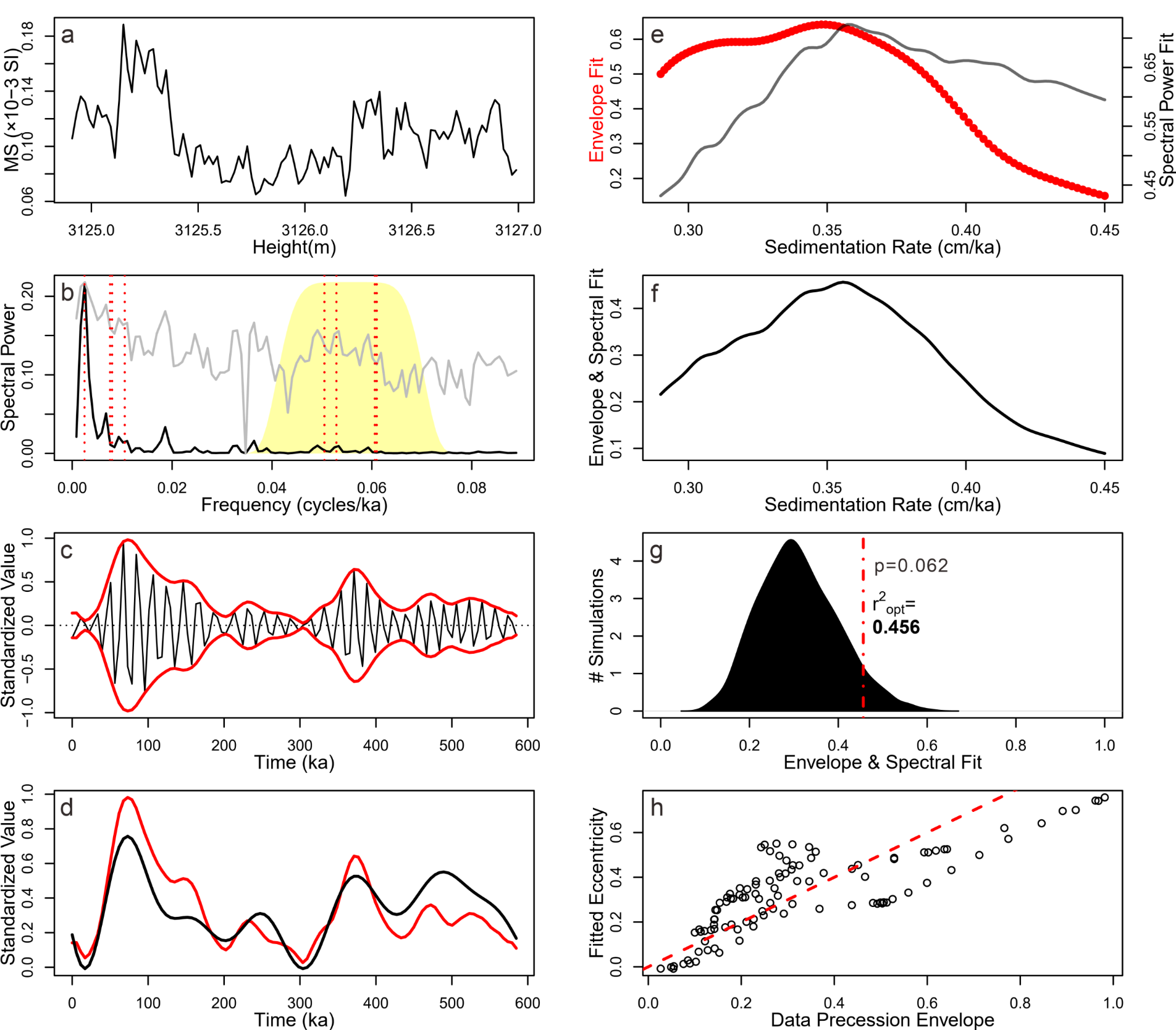


**Supplementary Fig. 10** **TimeOpt analysis of the MS data (3123.35-3154.37 m) from the EYC2 drillcore.** (A) The MS data from the interval of 3124.9-3127.0 m that is analyzed. (B) Periodogram for the MS data from the interval of 3124.9-3127.0 m, given the TimeOpt derived average sedimentation rate of 0.35 cm/ka (black line = linear spectrum; gray line = log spectrum). Yellow shaded region illustrates the bandpass filter for evaluation of the precession amplitude envelope. Vertical dashed red line indicates the precession and eccentricity target period. (C) Comparison of the band-passed precession signal (black line), and the data amplitude envelope (red line) determined via Hilbert transform. (D) Comparison of the data amplitude envelope (red line) and the TimeOpt-reconstructed eccentricity amplitude modulation model (black line; derived using Eq. (1) in ref. 85). (E) Squared Pearson correlation coefficient for the amplitude envelope fit (r^2^_envelope_; red dots) and the spectral power fit (r^2^_spectral_; dark gray line) at each evaluated sedimentation rate. (F) Combined envelope and spectral power fit (r^2^_opt_) at each evaluated sedimentation rate. (G) Summary of 2000 Monte Carlo simulations with AR1 surrogates (ρ_AR1_ = 0.77), used to evaluate the significance of the maximum observed r^2^_opt_ of 0.456 (p-value =0.062). (H) Cross plot of the data amplitude envelope and the TimeOpt-reconstructed precession amplitude modulation model in panel “d”; dashed red line is the 1:1 line. Detailed settings and parameters for the TimeOpt analysis of the EYC2 drillcore are provided in Supplementary Text 1.1.


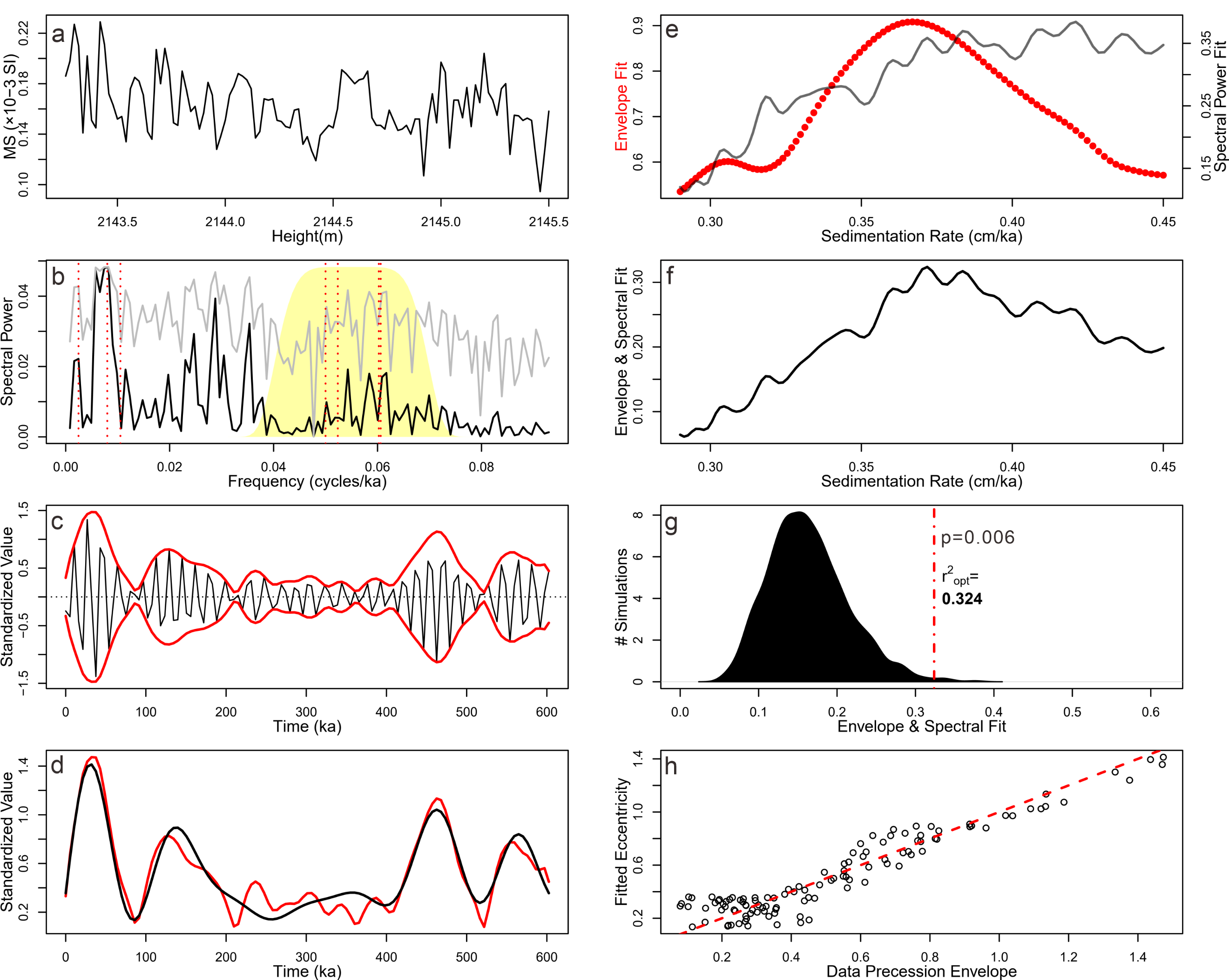


**Supplementary Fig. 11** **TimeOpt analysis of the MS data (2132-2155.4 m) from Subset D2-1 in the WD1 drillcore.** (A) The MS data from the interval of 2143.25-2145.5 m that is analyzed. (B) Periodogram for the MS data from the interval of 2143.25-2145.5 m, given the TimeOpt derived average sedimentation rate of 0.37 cm/ka (black line = linear spectrum; gray line = log spectrum). Yellow shaded region illustrates the bandpass filter for evaluation of the precession amplitude envelope. Vertical dashed red line indicates the precession and eccentricity target period. (C) Comparison of the band-passed precession signal (black line), and the data amplitude envelope (red line) determined via Hilbert transform. (D) Comparison of the data amplitude envelope (red line) and the TimeOpt-reconstructed eccentricity amplitude modulation model (black line; derived using Eq. (1) in ref. 85). (E) Squared Pearson correlation coefficient for the amplitude envelope fit (r^2^_envelope_; red dots) and the spectral power fit (r^2^_spectral_; dark gray line) at each evaluated sedimentation rate. (F) Combined envelope and spectral power fit (r^2^_opt_) at each evaluated sedimentation rate. (G) Summary of 2000 Monte Carlo simulations with AR1 surrogates (ρ_AR1_ = 0.379), used to evaluate the significance of the maximum observed r^2^_opt_ of 0.324 (p-value =0.006). (H) Cross plot of the data amplitude envelope and the TimeOpt-reconstructed precession amplitude modulation model in panel “d”; dashed red line is the 1:1 line. Detailed settings and parameters for the TimeOpt analysis of Subset D2-1 in the WD1 drillcore are provided in Supplementary Text 1.2.


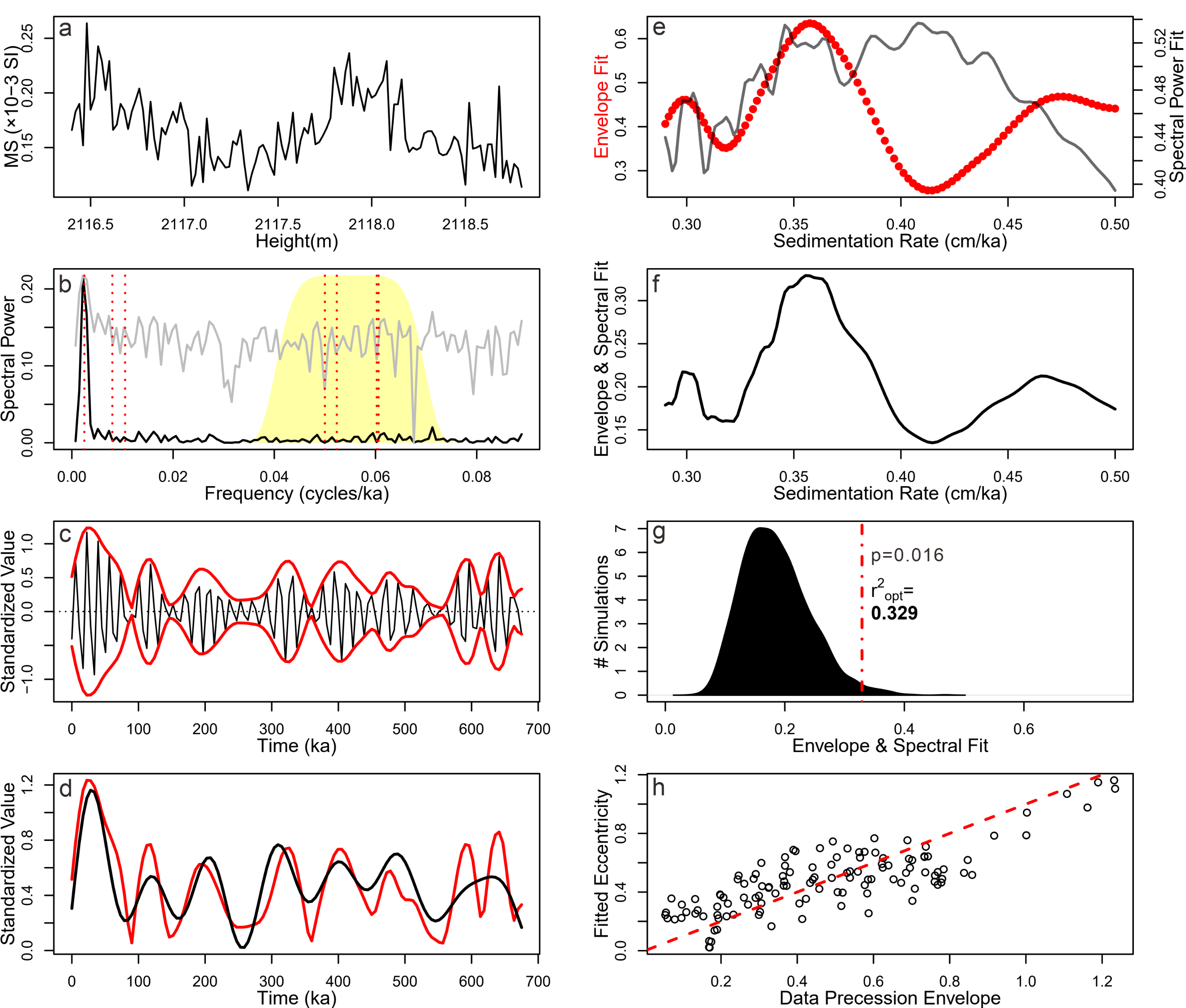


**Supplementary Fig. 12 TimeOpt analysis of the MS data (2110-2132 m) from Subset D2-2 in the WD1 drillcore.** (A) The MS data from the interval of 2116.4-2118.8 m that is analyzed. (B) Periodogram for the MS data from the interval of 2116.4-2118.8 m, given the TimeOpt derived average sedimentation rate of 0.35 cm/ka (black line = linear spectrum; gray line = log spectrum). Yellow shaded region illustrates the bandpass filter for evaluation of the precession amplitude envelope. Vertical dashed red line indicates the precession and eccentricity target period. (C) Comparison of the band-passed precession signal (black line), and the data amplitude envelope (red line) determined via Hilbert transform. (D) Comparison of the data amplitude envelope (red line) and the TimeOpt-reconstructed eccentricity amplitude modulation model (black line; derived using Eq. (1) in ref. 85). (E) Squared Pearson correlation coefficient for the amplitude envelope fit (r^2^_envelope_; red dots) and the spectral power fit (r^2^_spectral_; dark gray line) at each evaluated sedimentation rate. (F) Combined envelope and spectral power fit (r^2^_opt_) at each evaluated sedimentation rate. (G) Summary of 2000 Monte Carlo simulations with AR1 surrogates (ρ_AR1_ = 0.503), used to evaluate the significance of the maximum observed r^2^_opt_ of 0.329 (p-value =0.016). (H) Cross plot of the data amplitude envelope and the TimeOpt-reconstructed precession amplitude modulation model in panel “d”; dashed red line is the 1:1 line. Detailed settings and parameters for the TimeOpt analysis of Subset D2-2 in the WD1 drillcore are provided in Supplementary Text 1.3.


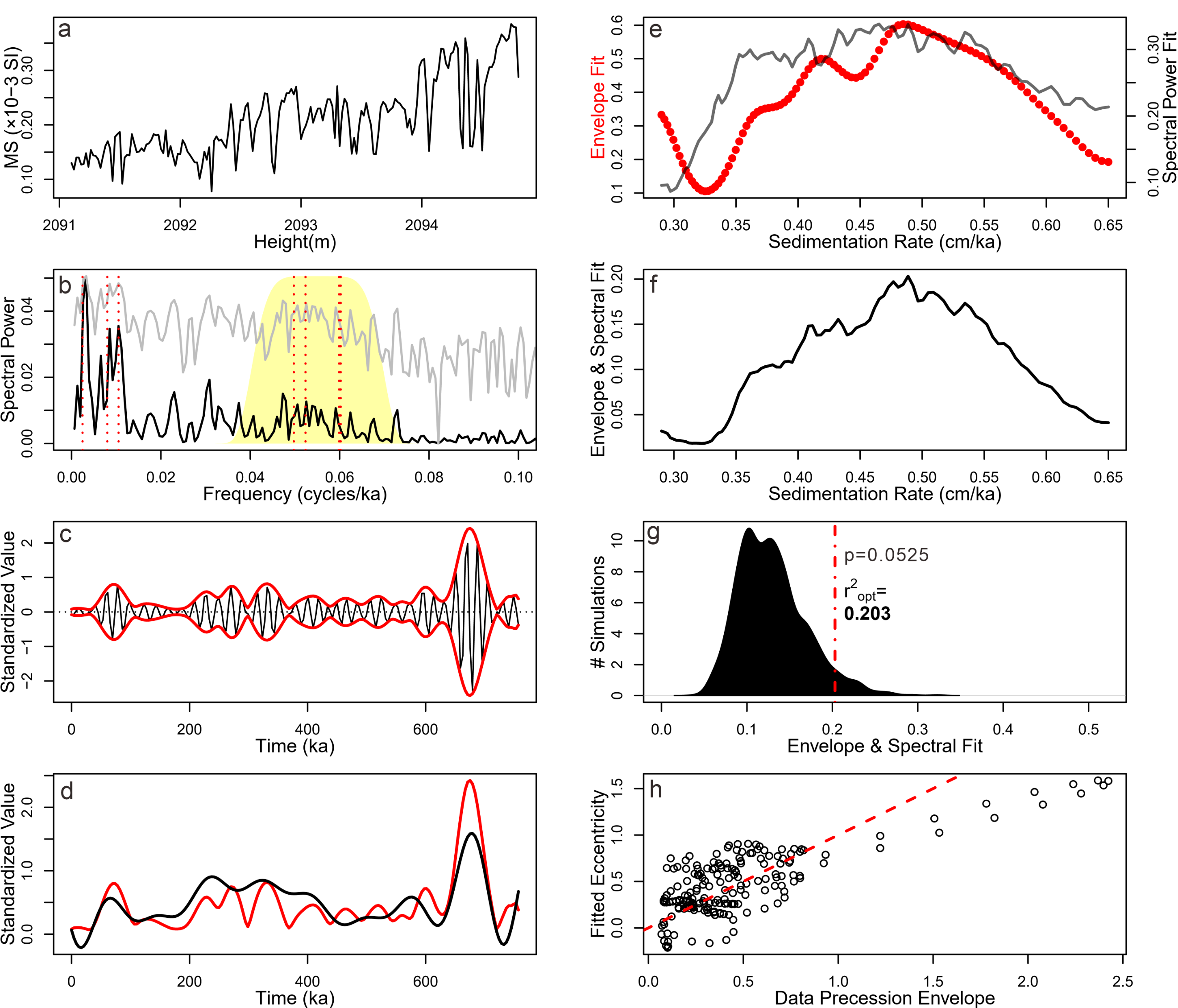


**Supplementary Fig. 13 TimeOpt analysis of the MS data (2088-2110 m) from Subset D2-3 in the WD1 drillcore.** (A) The MS data from the interval of 2091.1-2094.8 m that is analyzed. (B) Periodogram for the MS data from the interval of 2091.1-2094.8 m, given the TimeOpt derived average sedimentation rate of 0.49 cm/ka (black line = linear spectrum; gray line = log spectrum). Yellow shaded region illustrates the bandpass filter for evaluation of the precession amplitude envelope. Vertical dashed red line indicates the precession and eccentricity target period. (C) Comparison of the band-passed precession signal (black line), and the data amplitude envelope (red line) determined via Hilbert transform. (D) Comparison of the data amplitude envelope (red line) and the TimeOpt-reconstructed eccentricity amplitude modulation model (black line; derived using Eq. (1) in ref. 85). (E) Squared Pearson correlation coefficient for the amplitude envelope fit (r^2^_envelope_; red dots) and the spectral power fit (r^2^_spectral_; dark gray line) at each evaluated sedimentation rate. (F) Combined envelope and spectral power fit (r^2^_opt_) at each evaluated sedimentation rate. (G) Summary of 2000 Monte Carlo simulations with AR1 surrogates (ρ_AR1_ = 0.550), used to evaluate the significance of the maximum observed r^2^_opt_ of 0.203 (p-value =0.0525). (H) Cross plot of the data amplitude envelope and the TimeOpt-reconstructed precession amplitude modulation model in panel “d”; dashed red line is the 1:1 line. Detailed settings and parameters for the TimeOpt analysis of Subset D2-3 in the WD1 drillcore are provided in Supplementary Text 1.4.


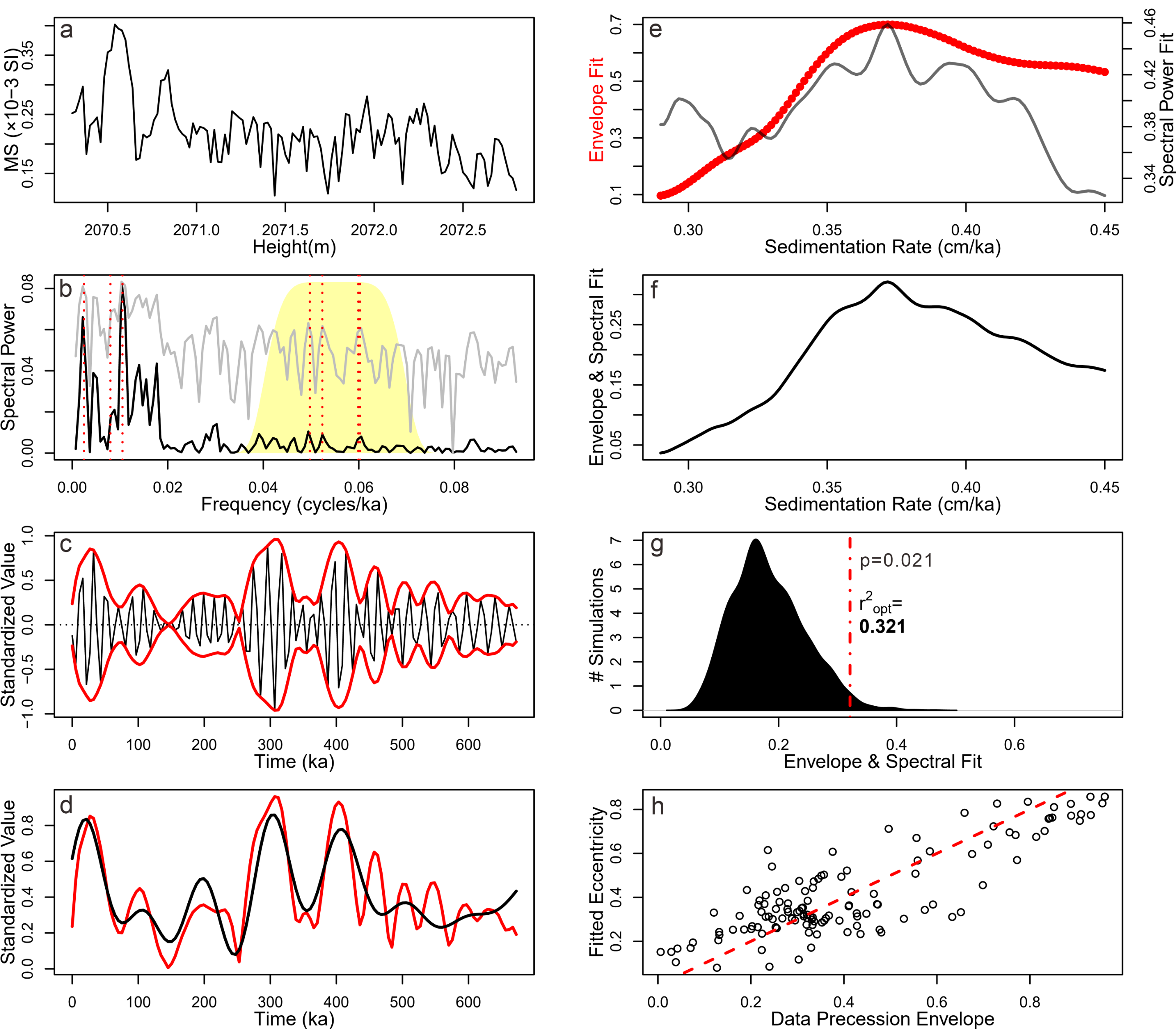


**Supplementary Fig. 14 TimeOpt analysis of the MS data (2067.2-2088 m) from Subset D2-4 in the WD1 drillcore.** (A) The MS data from the interval of 2070.3-2072.8 m that is analyzed. (B) Periodogram for the MS data from the interval of 2070.3-2072.8 m, given the TimeOpt derived average sedimentation rate of 0.37 cm/ka (black line = linear spectrum; gray line = log spectrum). Yellow shaded region illustrates the bandpass filter for evaluation of the precession amplitude envelope. Vertical dashed red line indicates the precession and eccentricity target period. (C) Comparison of the band-passed precession signal (black line), and the data amplitude envelope (red line) determined via Hilbert transform. (D) Comparison of the data amplitude envelope (red line) and the TimeOpt-reconstructed eccentricity amplitude modulation model (black line; derived using Eq. (1) in ref. 85). (E) Squared Pearson correlation coefficient for the amplitude envelope fit (r^2^_envelope_; red dots) and the spectral power fit (r^2^_spectral_; dark gray line) at each evaluated sedimentation rate. (F) Combined envelope and spectral power fit (r^2^_opt_) at each evaluated sedimentation rate. (G) Summary of 2000 Monte Carlo simulations with AR1 surrogates (ρ_AR1_ = 0.619), used to evaluate the significance of the maximum observed r^2^_opt_ of 0.321 (p-value =0.021). (H) Cross plot of the data amplitude envelope and the TimeOpt-reconstructed precession amplitude modulation model in panel “d”; dashed red line is the 1:1 line. Detailed settings and parameters for the TimeOpt analysis of Subset D2-4 in the WD1 drillcore are provided in Supplementary Text 1.5.


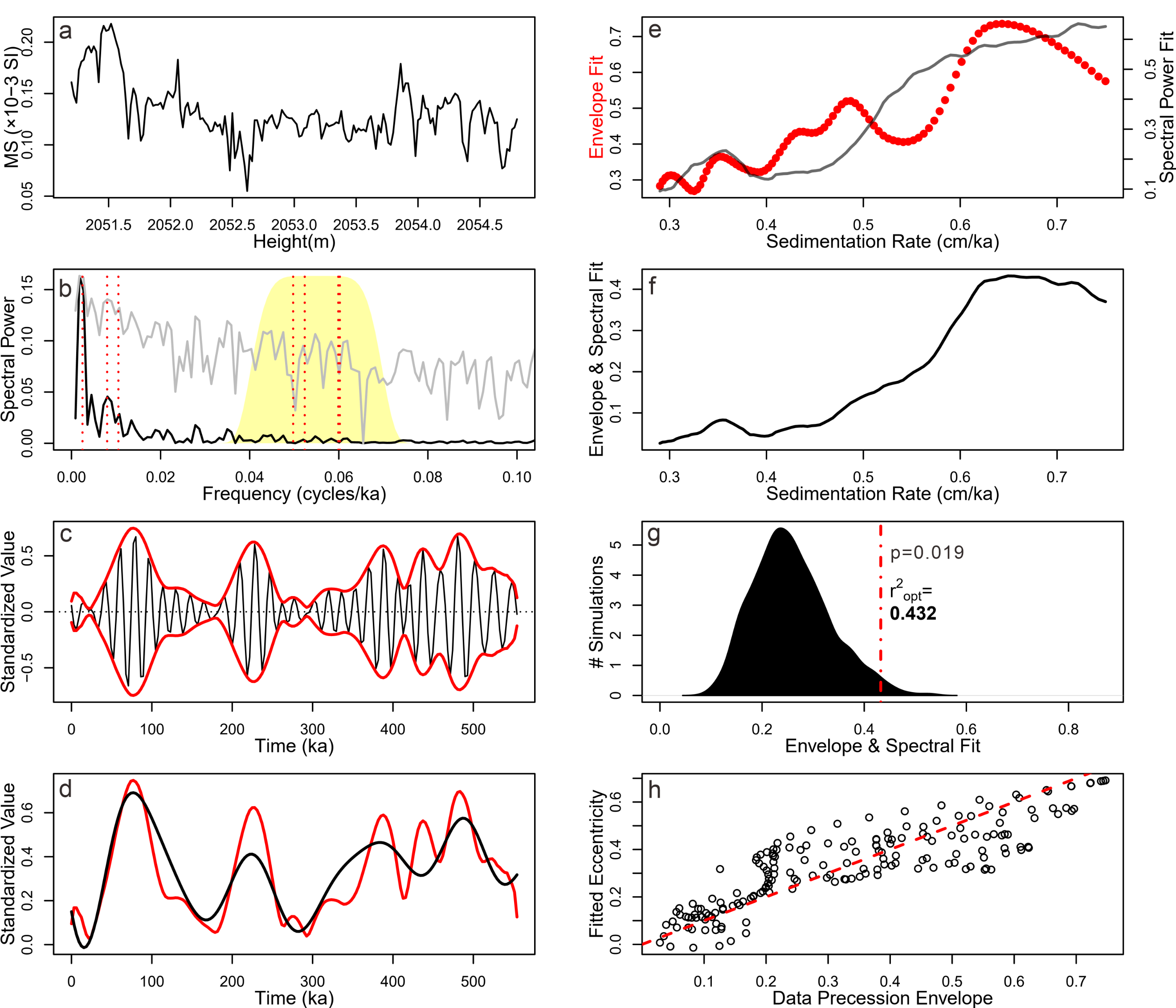


**Supplementary Fig. 15 TimeOpt analysis of the MS data (2040-2067.2 m) from Subset D3 in the WD1 drillcore.** (A) The MS data from the interval of 2051.2-2054.8 m that is analyzed. (B) Periodogram for the MS data from the interval of 2051.2-2054.8 m, given the TimeOpt derived average sedimentation rate of 0.65 cm/ka (black line = linear spectrum; gray line = log spectrum). Yellow shaded region illustrates the bandpass filter for evaluation of the precession amplitude envelope. Vertical dashed red line indicates the precession and eccentricity target period. (C) Comparison of the band-passed precession signal (black line), and the data amplitude envelope (red line) determined via Hilbert transform. (D) Comparison of the data amplitude envelope (red line) and the TimeOpt-reconstructed eccentricity amplitude modulation model (black line; derived using Eq. (1) in ref. 85). (E) Squared Pearson correlation coefficient for the amplitude envelope fit (r^2^_envelope_; red dots) and the spectral power fit (r^2^_spectral_; dark gray line) at each evaluated sedimentation rate. (F) Combined envelope and spectral power fit (r^2^_opt_) at each evaluated sedimentation rate. (G) Summary of 2000 Monte Carlo simulations with AR1 surrogates (ρ_AR1_ = 0.806), used to evaluate the significance of the maximum observed r^2^_opt_ of 0.432 (p-value =0.019). (H) Cross plot of the data amplitude envelope and the TimeOpt-reconstructed precession amplitude modulation model in panel “d”; dashed red line is the 1:1 line. Detailed settings and parameters for the TimeOpt analysis of Subset D3 in the WD1 drillcore are provided in Supplementary Text 1.6.


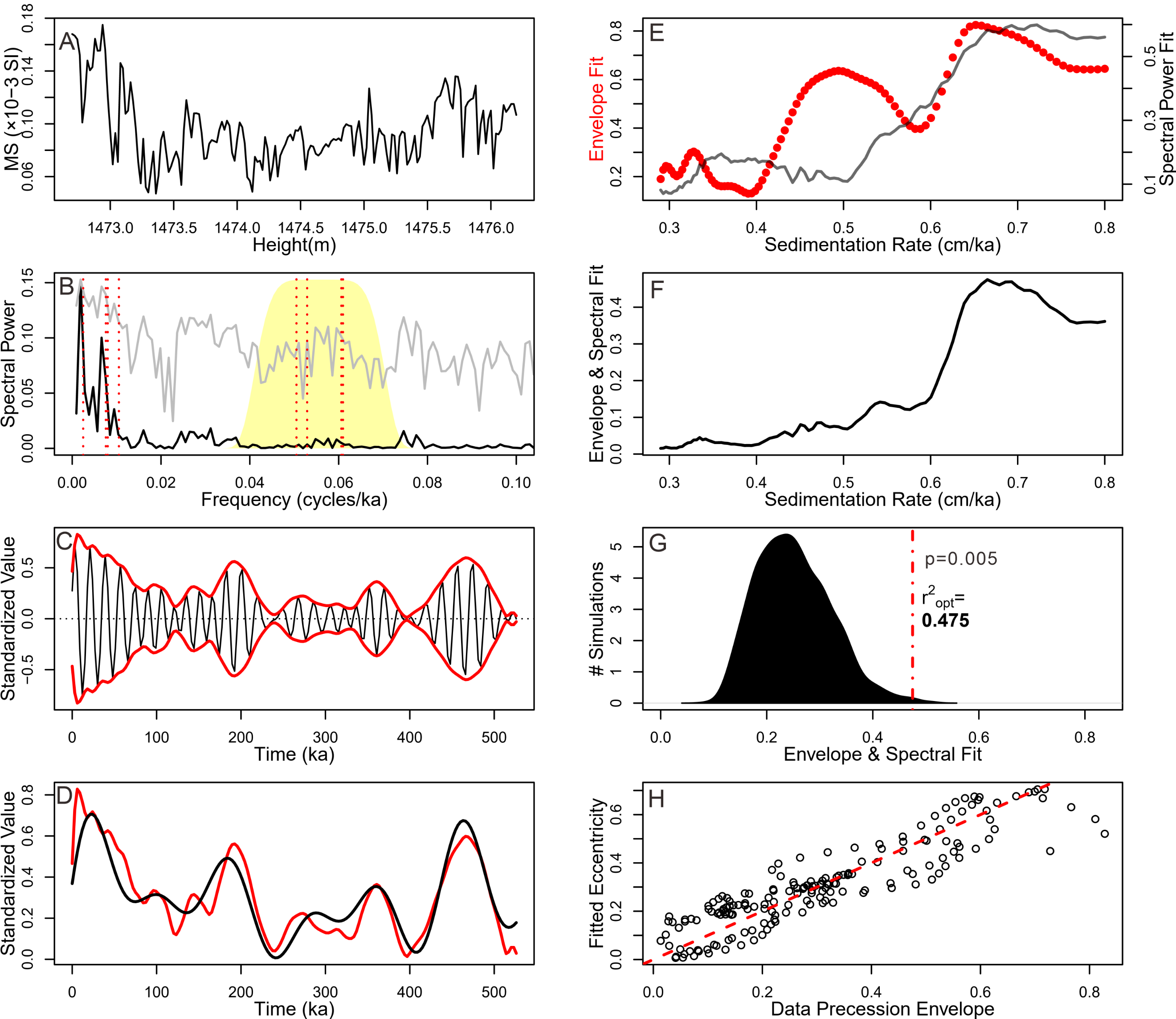


**Supplementary Fig. 16 TimeOpt analysis of the MS data (1479.5-1472 m) from Subset D1 in the ZK68 drillcore.** (A) The MS data from the interval of 1472.7-1476.2 m that is analyzed. (B) Periodogram for the MS data from the interval of 1472.7-1476.2 m, given the TimeOpt derived average sedimentation rate of 0.66 cm/ka (black line = linear spectrum; gray line = log spectrum). Yellow shaded region illustrates the bandpass filter for evaluation of the precession amplitude envelope. Vertical dashed red line indicates the precession and eccentricity target period. (C) Comparison of the band-passed precession signal (black line), and the data amplitude envelope (red line) determined via Hilbert transform. (D) Comparison of the data amplitude envelope (red line) and the TimeOpt-reconstructed eccentricity amplitude modulation model (black line; derived using Eq. (1) in ref. 85). (E) Squared Pearson correlation coefficient for the amplitude envelope fit (r^2^_envelope_; red dots) and the spectral power fit (r^2^_spectral_; dark gray line) at each evaluated sedimentation rate. (F) Combined envelope and spectral power fit (r^2^_opt_) at each evaluated sedimentation rate. (G) Summary of 2000 Monte Carlo simulations with AR1 surrogates (ρ_AR1_ = 0.749), used to evaluate the significance of the maximum observed r^2^_opt_ of 0.475 (p-value =0.005). (H) Cross plot of the data amplitude envelope and the TimeOpt-reconstructed precession amplitude modulation model in panel “d”; dashed red line is the 1:1 line. Detailed settings and parameters for the TimeOpt analysis of Subset D1 in the ZK68 drillcore are provided in Supplementary Text 1.7.


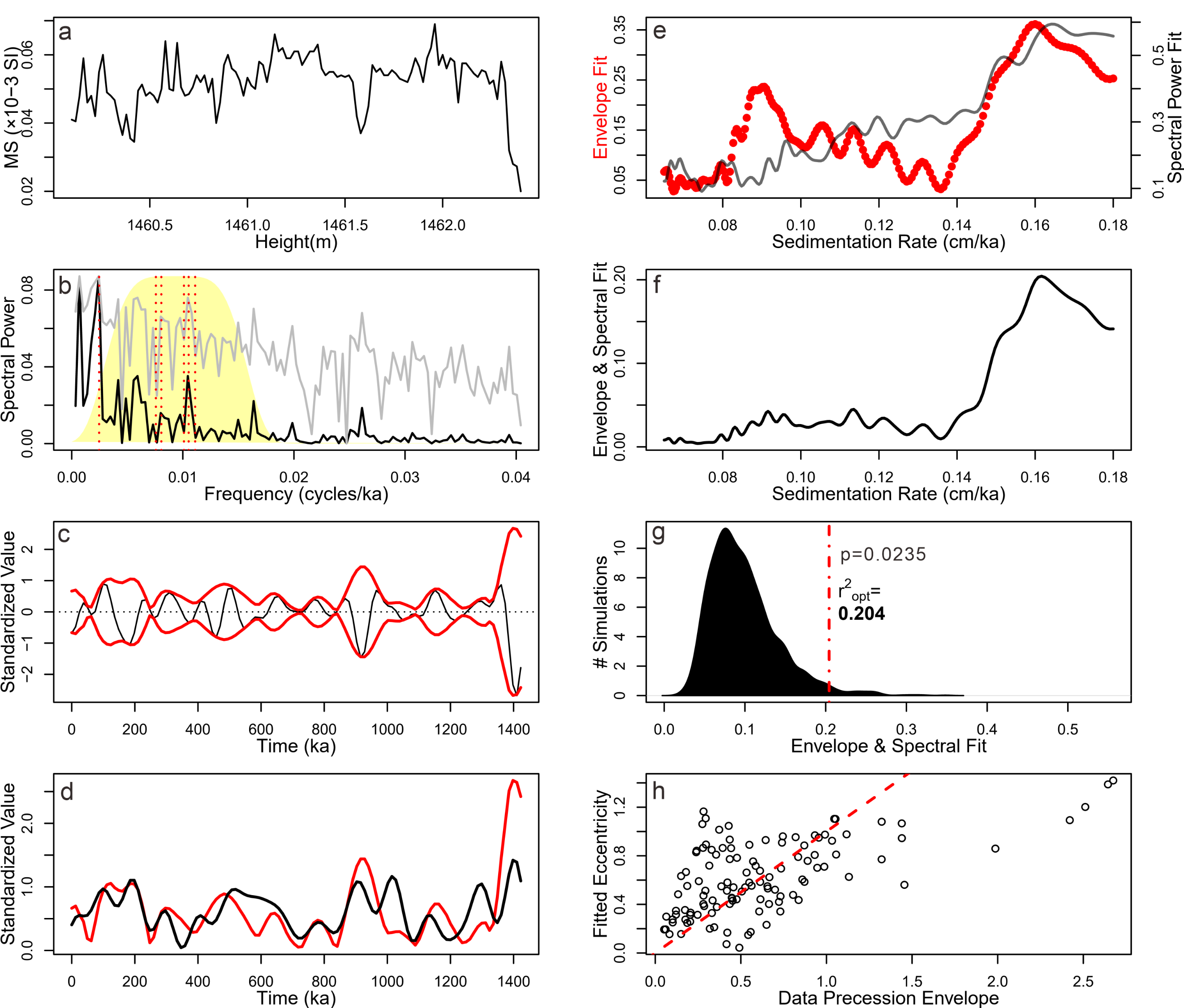


**Supplementary Fig. 17 TimeOpt analysis of the MS data (1471.99-1458.38 m) from Subset D2-1 in the ZK68 drillcore.** (A) The MS data from the interval of 1460.1-1462.4 m that is analyzed. (B) Periodogram for the MS data from the interval of 1460.1-1462.4 m, given the TimeOpt derived average sedimentation rate of 0.16 cm/ka (black line = linear spectrum; gray line = log spectrum). Yellow shaded region illustrates the bandpass filter for evaluation of the precession amplitude envelope. Vertical dashed red line indicates the precession and eccentricity target period. (C) Comparison of the band-passed precession signal (black line), and the data amplitude envelope (red line) determined via Hilbert transform. (D) Comparison of the data amplitude envelope (red line) and the TimeOpt-reconstructed eccentricity amplitude modulation model (black line; derived using Eq. (1) in ref. 85). (E) Squared Pearson correlation coefficient for the amplitude envelope fit (r^2^_envelope_; red dots) and the spectral power fit (r^2^_spectral_; dark gray line) at each evaluated sedimentation rate. (F) Combined envelope and spectral power fit (r^2^_opt_) at each evaluated sedimentation rate. (G) Summary of 2000 Monte Carlo simulations with AR1 surrogates (ρ_AR1_ = 0.693), used to evaluate the significance of the maximum observed r^2^_opt_ of 0.204 (p-value =0.0235). (H) Cross plot of the data amplitude envelope and the TimeOpt-reconstructed precession amplitude modulation model in panel “d”; dashed red line is the 1:1 line. Detailed settings and parameters for the TimeOpt analysis of Subset D2-1 in the ZK68 drillcore are provided in Supplementary Text 1.8.


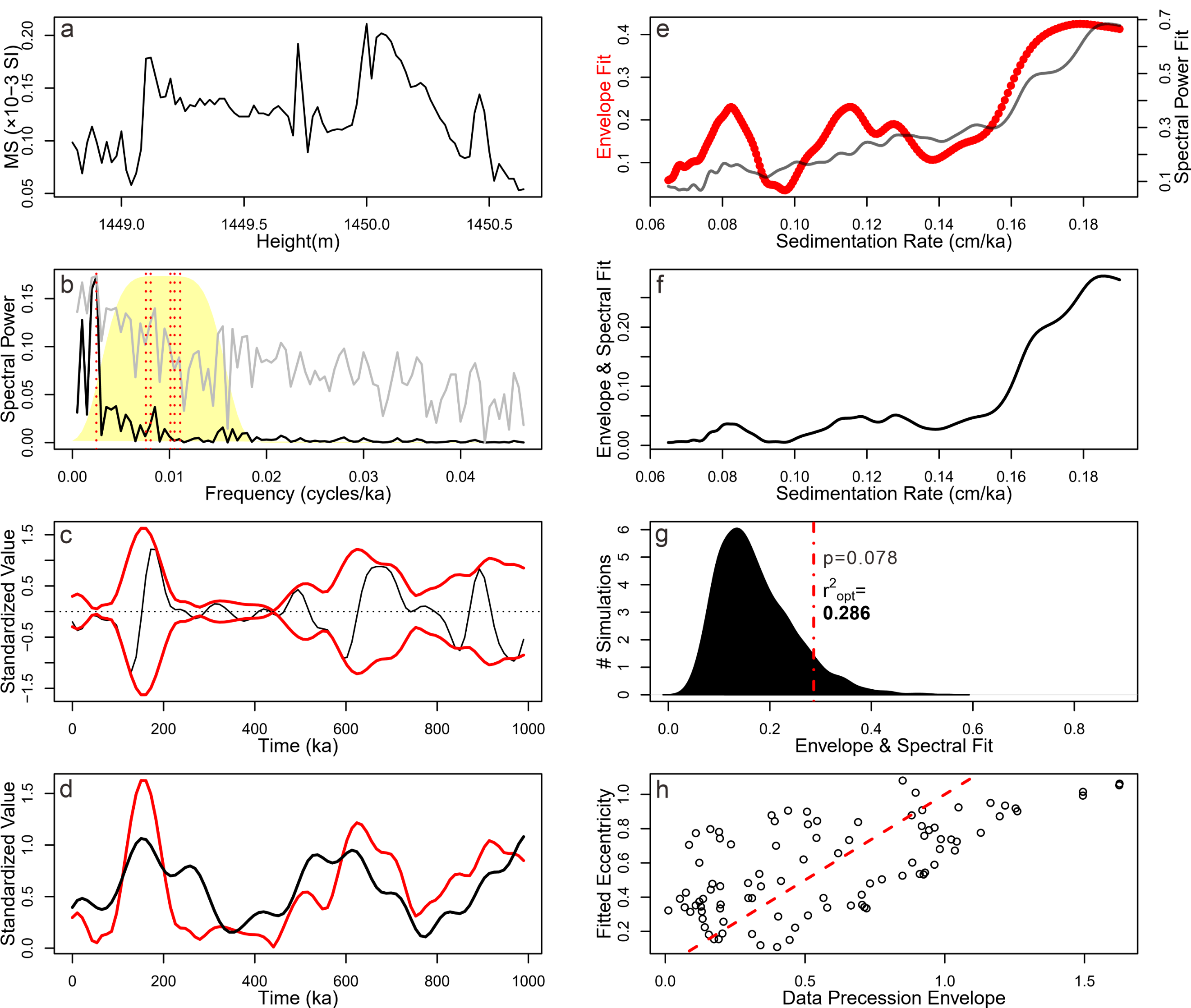


**Supplementary Fig. 18 TimeOpt analysis of the MS data from Subset D2-2 (1458.38-1445.72 m) in the ZK68 drillcore.** (A) The MS data from the interval of 1448.8-1450.65 m that is analyzed. (B) Periodogram for the MS data from the interval of 1448.8-1450.65 m, given the TimeOpt derived average sedimentation rate of 0.18 cm/ka (black line = linear spectrum; gray line = log spectrum). Yellow shaded region illustrates the bandpass filter for evaluation of the precession amplitude envelope. Vertical dashed red line indicates the precession and eccentricity target period. (C) Comparison of the band-passed precession signal (black line), and the data amplitude envelope (red line) determined via Hilbert transform. (D) Comparison of the data amplitude envelope (red line) and the TimeOpt-reconstructed eccentricity amplitude modulation model (black line; derived using Eq. (1) in ref. 85). (E) Squared Pearson correlation coefficient for the amplitude envelope fit (r^2^_envelope_; red dots) and the spectral power fit (r^2^_spectral_; dark gray line) at each evaluated sedimentation rate. (F) Combined envelope and spectral power fit (r^2^_opt_) at each evaluated sedimentation rate. (G) Summary of 2000 Monte Carlo simulations with AR1 surrogates (ρ_AR1_ = 0.813), used to evaluate the significance of the maximum observed r^2^_opt_ of 0.286 (p-value =0.078). (H) Cross plot of the data amplitude envelope and the TimeOpt-reconstructed precession amplitude modulation model in panel “d”; dashed red line is the 1:1 line. Detailed settings and parameters for the TimeOpt analysis of Subset D2-2 in the ZK68 drillcore are provided in Supplementary Text 1.9.


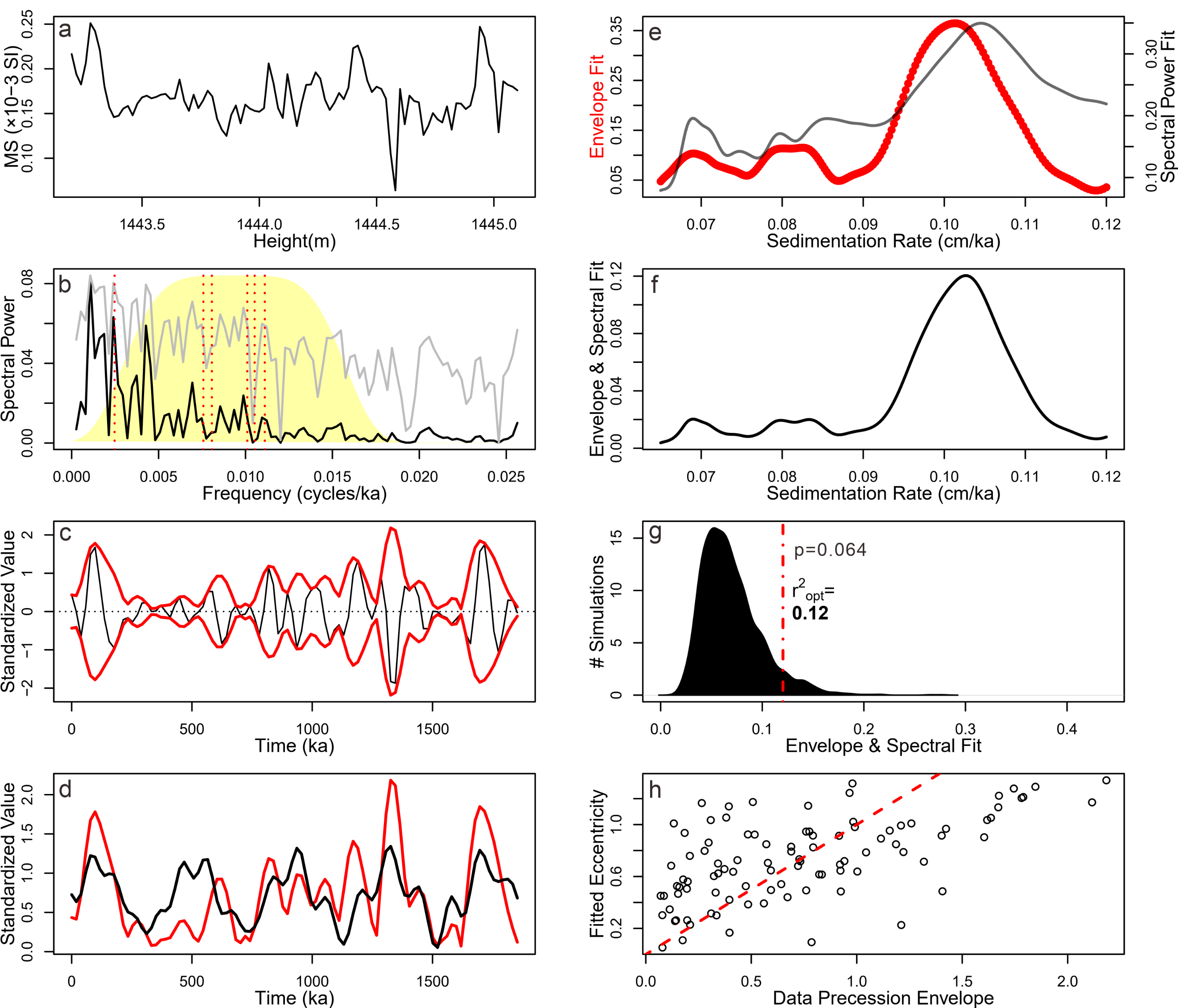


**Supplementary Fig. 19 TimeOpt analysis of the MS data from Subset D2-3 (1445.72-1430 m) in the ZK68 drillcore.** (A) The MS data from the interval of 1443.2-1445.1 m that is analyzed. (B) Periodogram for the MS data from the interval of 1443.2-1445.1 m, given the TimeOpt derived average sedimentation rate of 0.10 cm/ka (black line = linear spectrum; gray line = log spectrum). Yellow shaded region illustrates the bandpass filter for evaluation of the precession amplitude envelope. Vertical dashed red line indicates the precession and eccentricity target period. (C) Comparison of the band-passed precession signal (black line), and the data amplitude envelope (red line) determined via Hilbert transform. (D) Comparison of the data amplitude envelope (red line) and the TimeOpt-reconstructed eccentricity amplitude modulation model (black line; derived using Eq. (1) in ref. 85). (E) Squared Pearson correlation coefficient for the amplitude envelope fit (r^2^_envelope_; red dots) and the spectral power fit (r^2^_spectral_; dark gray line) at each evaluated sedimentation rate. (F) Combined envelope and spectral power fit (r^2^_opt_) at each evaluated sedimentation rate. (G) Summary of 2000 Monte Carlo simulations with AR1 surrogates (ρ_AR1_ = 0.603), used to evaluate the significance of the maximum observed r^2^_opt_ of 0.12 (p-value =0.064). (H) Cross plot of the data amplitude envelope and the TimeOpt-reconstructed precession amplitude modulation model in panel “d”; dashed red line is the 1:1 line. Detailed settings and parameters for the TimeOpt analysis of Subset D2-3 in the ZK68 drillcore are provided in Supplementary Text 1.10.


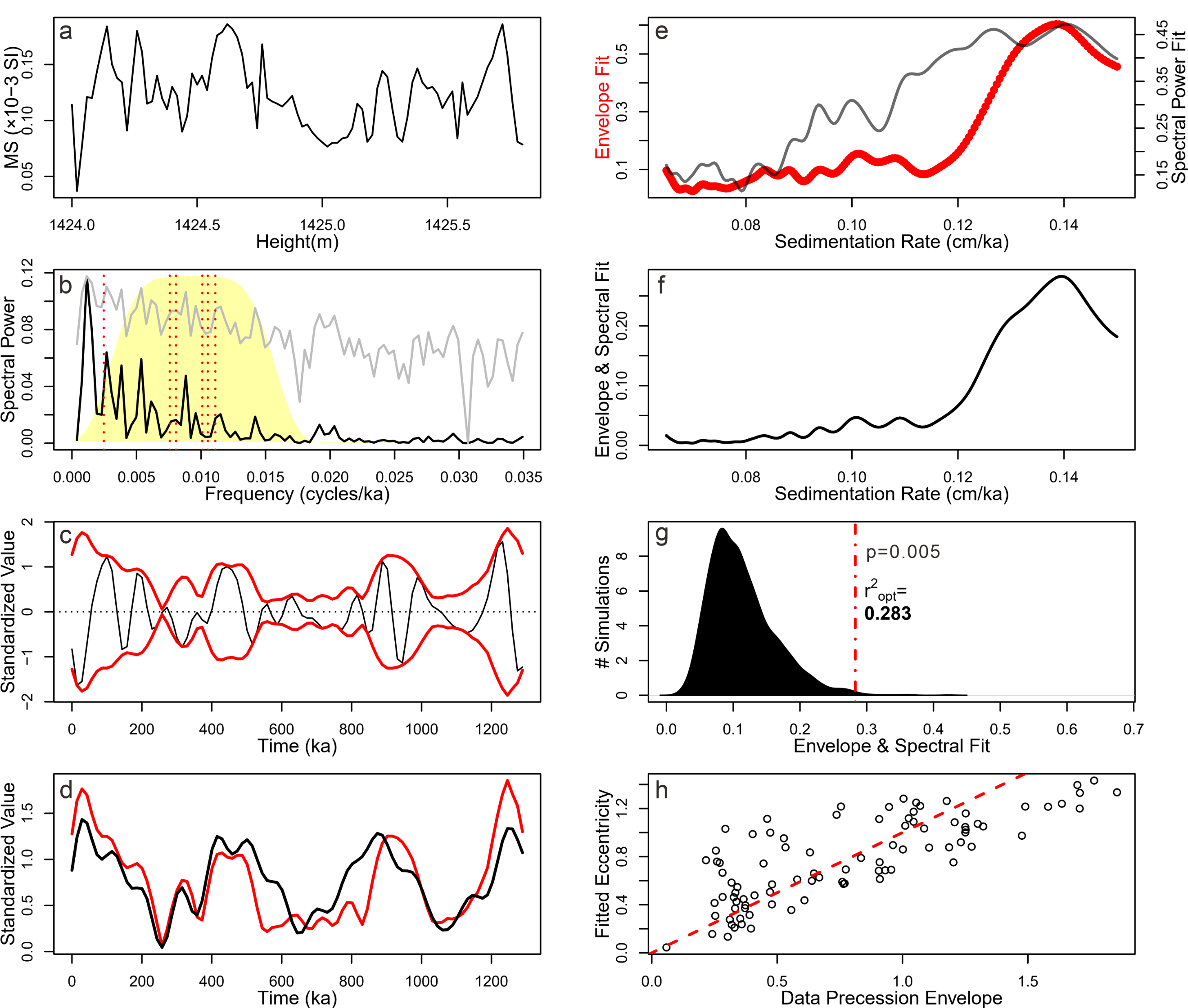


**Supplementary Fig. 20 TimeOpt analysis of the MS data from Subset D2-4 (1430-1413.5 m) in the ZK68 drillcore.** (A) The MS data from the interval of 1424-1425.8 m that is analyzed. (B) Periodogram for the MS data from the interval of 1424-1425.8 m, given the TimeOpt derived average sedimentation rate of 0.14 cm/ka (black line = linear spectrum; gray line = log spectrum). Yellow shaded region illustrates the bandpass filter for evaluation of the precession amplitude envelope. Vertical dashed red line indicates the precession and eccentricity target period. (C) Comparison of the band-passed precession signal (black line), and the data amplitude envelope (red line) determined via Hilbert transform. (D) Comparison of the data amplitude envelope (red line) and the TimeOpt-reconstructed eccentricity amplitude modulation model (black line; derived using Eq. (1) in ref. 85). (E) Squared Pearson correlation coefficient for the amplitude envelope fit (r^2^_envelope_; red dots) and the spectral power fit (r^2^_spectral_; dark gray line) at each evaluated sedimentation rate. (F) Combined envelope and spectral power fit (r^2^_opt_) at each evaluated sedimentation rate. (G) Summary of 2000 Monte Carlo simulations with AR1 surrogates (ρ_AR1_ = 0.679), used to evaluate the significance of the maximum observed r^2^_opt_ of 0.283 (p-value =0.005). (H) Cross plot of the data amplitude envelope and the TimeOpt-reconstructed precession amplitude modulation model in panel “d”; dashed red line is the 1:1 line. Detailed settings and parameters for the TimeOpt analysis of Subset D2-4 in the ZK68 drillcore are provided in Supplementary Text 1.11.


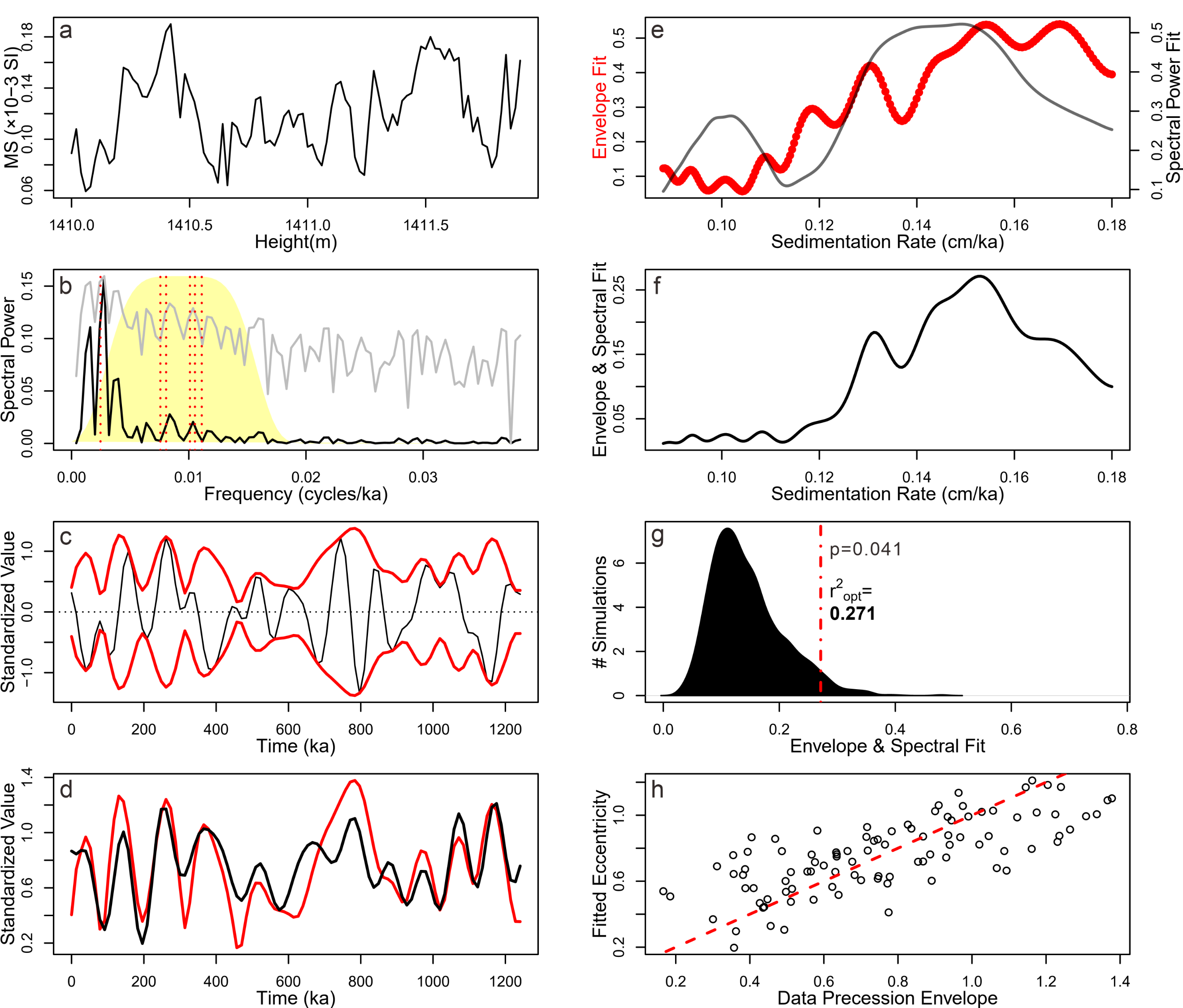


**Supplementary Fig. 21 TimeOpt analysis of the MS data from Subset D2-5 (1413.5-1399.4 m) in the ZK68 drillcore.** (A) The MS data from the interval of 1410-1411.9 m that is analyzed. (B) Periodogram for the MS data from the interval of 1410-1411.9 m, given the TimeOpt derived average sedimentation rate of 0.15 cm/ka (black line = linear spectrum; gray line = log spectrum). Yellow shaded region illustrates the bandpass filter for evaluation of the precession amplitude envelope. Vertical dashed red line indicates the precession and eccentricity target period. (C) Comparison of the band-passed precession signal (black line), and the data amplitude envelope (red line) determined via Hilbert transform. (D) Comparison of the data amplitude envelope (red line) and the TimeOpt-reconstructed eccentricity amplitude modulation model (black line; derived using Eq. (1) in ref. 85). (E) Squared Pearson correlation coefficient for the amplitude envelope fit (r^2^_envelope_; red dots) and the spectral power fit (r^2^_spectral_; dark gray line) at each evaluated sedimentation rate. (F) Combined envelope and spectral power fit (r^2^_opt_) at each evaluated sedimentation rate. (G) Summary of 2000 Monte Carlo simulations with AR1 surrogates (ρ_AR1_ = 0.758), used to evaluate the significance of the maximum observed r^2^_opt_ of 0.271 (p-value =0.041). (H) Cross plot of the data amplitude envelope and the TimeOpt-reconstructed precession amplitude modulation model in panel “d”; dashed red line is the 1:1 line. Detailed settings and parameters for the TimeOpt analysis of Subset D2-5 in the ZK68 drillcore are provided in Supplementary Text 1.12.


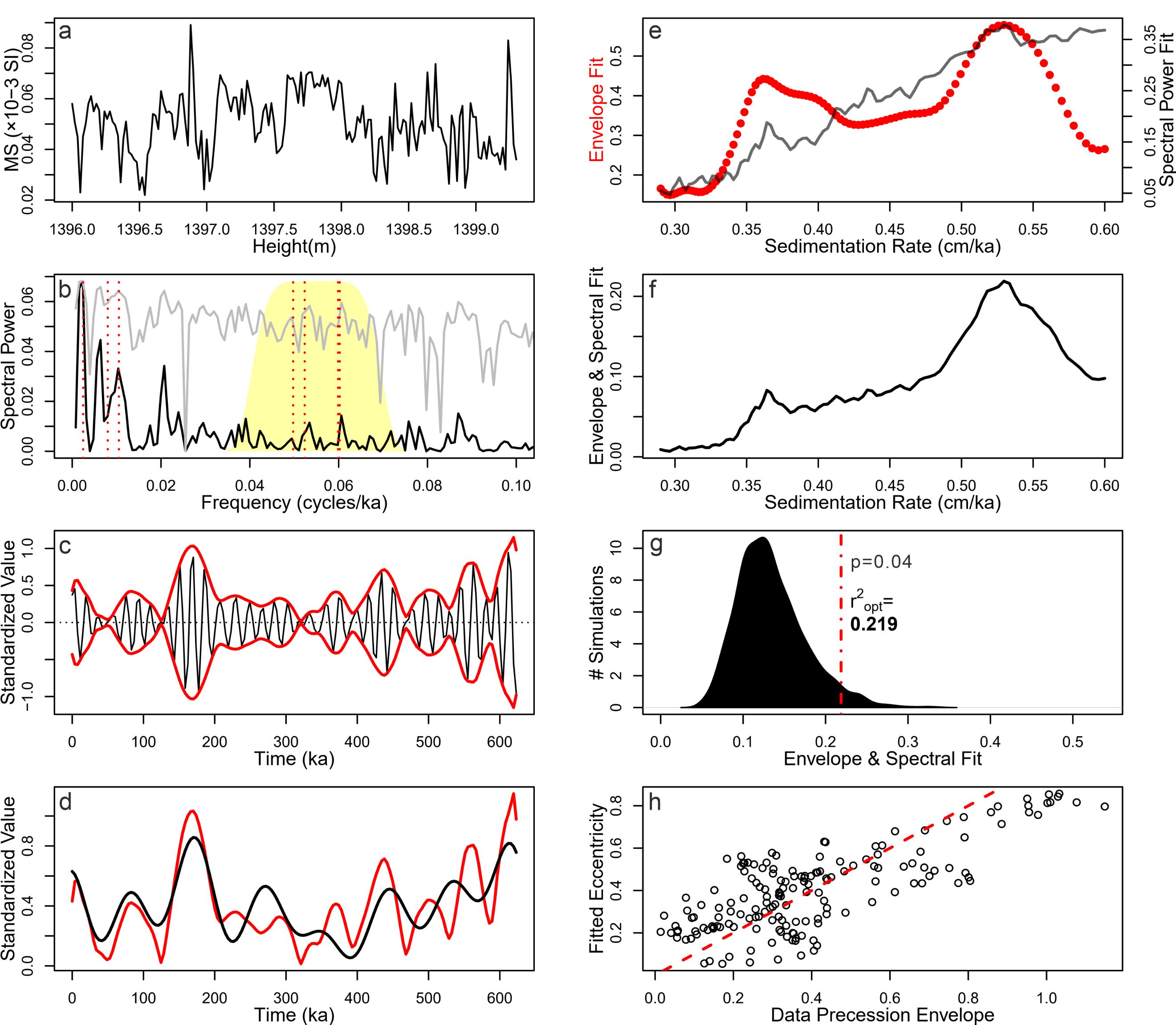


**Supplementary Fig. 22 TimeOpt analysis of the MS data from Subset D3 (1399.4-1332.28 m) in the ZK68 drillcore.** (A) The MS data from the interval of 1396-1399.3 m that is analyzed. (B) Periodogram for the MS data from the interval of 1396-1399.3 m, given the TimeOpt derived average sedimentation rate of 0.53 cm/ka (black line = linear spectrum; gray line = log spectrum). Yellow shaded region illustrates the bandpass filter for evaluation of the precession amplitude envelope. Vertical dashed red line indicates the precession and eccentricity target period. (C) Comparison of the band-passed precession signal (black line), and the data amplitude envelope (red line) determined via Hilbert transform. (D) Comparison of the data amplitude envelope (red line) and the TimeOpt-reconstructed eccentricity amplitude modulation model (black line; derived using Eq. (1) in ref. 85). (E) Squared Pearson correlation coefficient for the amplitude envelope fit (r^2^_envelope_; red dots) and the spectral power fit (r^2^_spectral_; dark gray line) at each evaluated sedimentation rate. (F) Combined envelope and spectral power fit (r^2^_opt_) at each evaluated sedimentation rate. (G) Summary of 2000 Monte Carlo simulations with AR1 surrogates (ρ_AR1_ = 0.499), used to evaluate the significance of the maximum observed r^2^_opt_ of 0.219 (p-value =0.04). (H) Cross plot of the data amplitude envelope and the TimeOpt-reconstructed precession amplitude modulation model in panel “d”; dashed red line is the 1:1 line. Detailed settings and parameters for the TimeOpt analysis of Subset D3 in the ZK68 drillcore are provided in Supplementary Text 1.13.


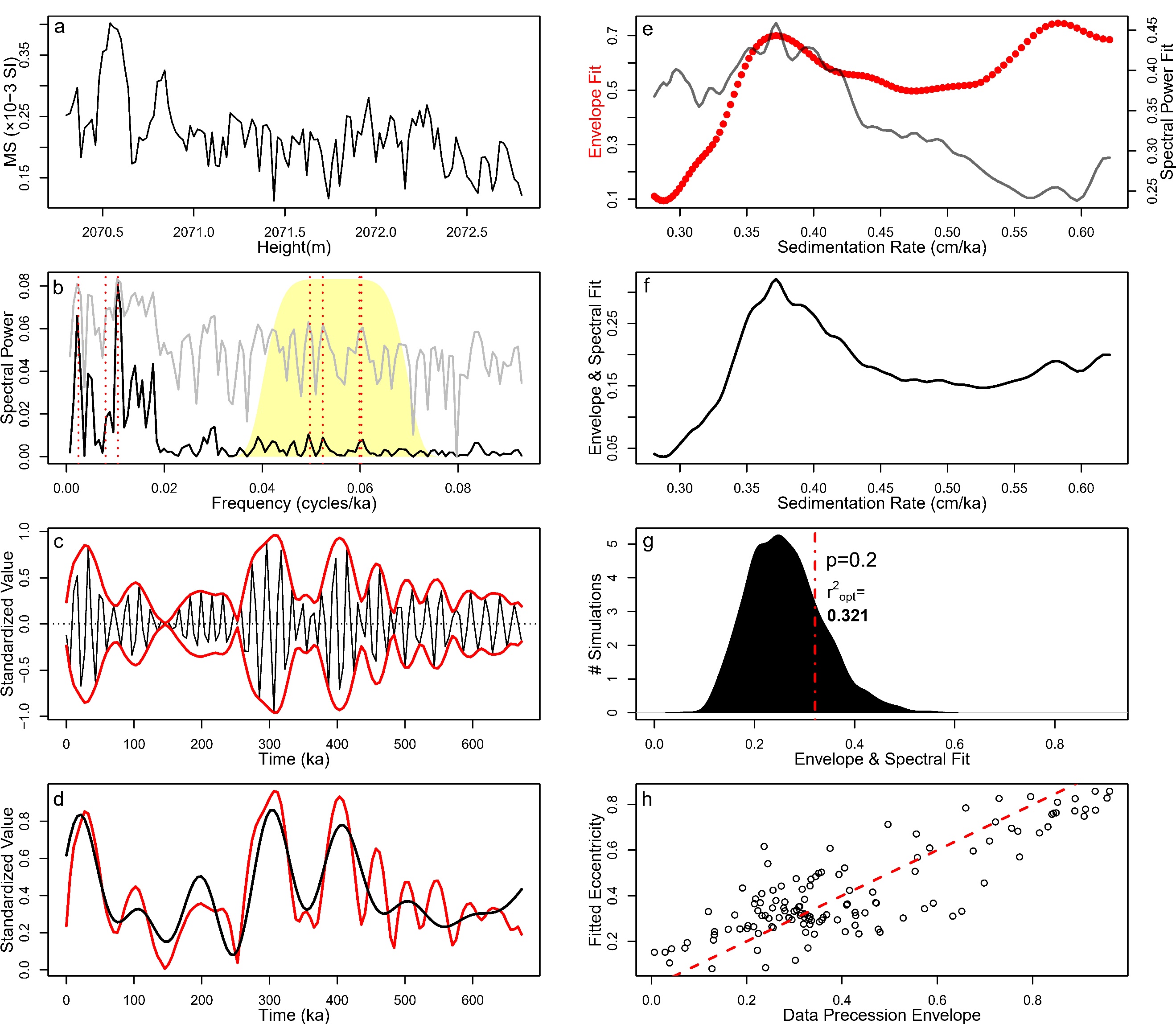


**Supplementary Fig. 23 Supplementary TimeOpt analyses of the MS data from Subset D2-4 (2067.2-2088 m) in the WD1 drillcore using expanded sedimentation rate ranges.** (A) The MS data from the interval of 2070.3-2072.8 m that is analyzed. (B) Periodogram for the MS data from the interval of 2070.3-2072.8 m, given the TimeOpt derived average sedimentation rate of 0.37 cm/ka (black line = linear spectrum; gray line = log spectrum). Yellow shaded region illustrates the bandpass filter for evaluation of the precession amplitude envelope. Vertical dashed red line indicates the precession and eccentricity target period. (C) Comparison of the band-passed precession signal (black line), and the data amplitude envelope (red line) determined via Hilbert transform. (D) Comparison of the data amplitude envelope (red line) and the TimeOpt-reconstructed eccentricity amplitude modulation model (black line; derived using Eq. (1) in ref. 85). (E) Squared Pearson correlation coefficient for the amplitude envelope fit (r^2^_envelope_; red dots) and the spectral power fit (r^2^_spectral_; dark gray line) at each evaluated sedimentation rate. (F) Combined envelope and spectral power fit (r^2^_opt_) at each evaluated sedimentation rate. (G) Summary of 2000 Monte Carlo simulations with AR1 surrogates (ρ_AR1_ = 0.6199), used to evaluate the significance of the maximum observed r^2^_opt_ of 0.321 (p-value =0.2). (H) Cross plot of the data amplitude envelope and the TimeOpt-reconstructed precession amplitude modulation model in panel “d”; dashed red line is the 1:1 line. Detailed settings and parameters for the Supplementary TimeOpt analysis of Subset D2-4 in the WD1 drillcore are provided in Supplementary Text 1.14.


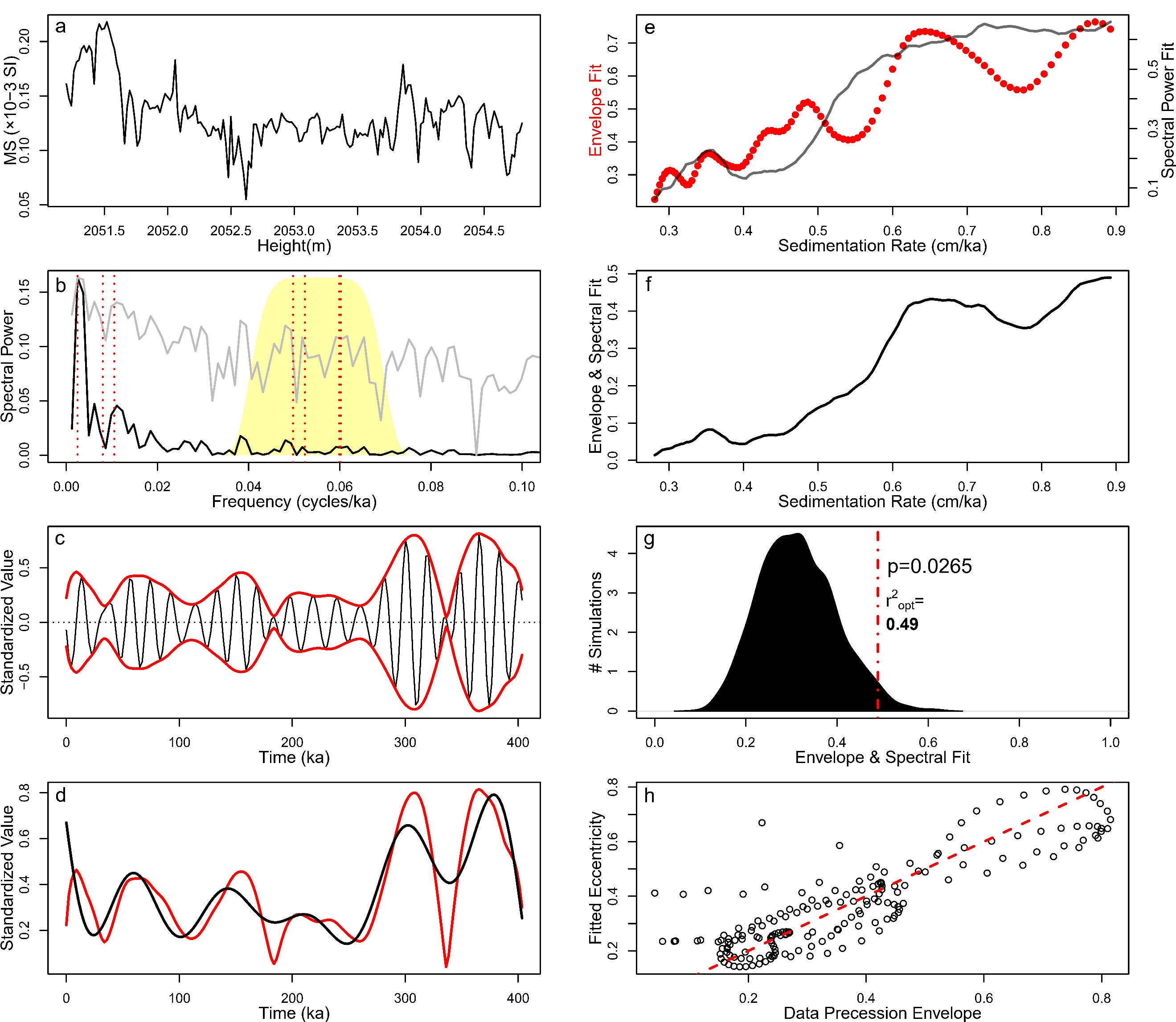


**Supplementary Fig. 24 Supplementary TimeOpt analyses of the MS data from Subset D3 (2040-2067.2 m) in the WD1 drillcore using expanded sedimentation rate ranges.** (A) The MS data from the interval of 2051.2-2054.8 m that is analyzed. (B) Periodogram for the MS data from the interval of 2051.2-2054.8 m, given the TimeOpt derived average sedimentation rate of 0.89 cm/ka (black line = linear spectrum; gray line = log spectrum). Yellow shaded region illustrates the bandpass filter for evaluation of the precession amplitude envelope. Vertical dashed red line indicates the precession and eccentricity target period. (C) Comparison of the band-passed precession signal (black line), and the data amplitude envelope (red line) determined via Hilbert transform. (D) Comparison of the data amplitude envelope (red line) and the TimeOpt-reconstructed eccentricity amplitude modulation model (black line; derived using Eq. (1) in ref. 85). (E) Squared Pearson correlation coefficient for the amplitude envelope fit (r^2^_envelope_; red dots) and the spectral power fit (r^2^_spectral_; dark gray line) at each evaluated sedimentation rate. (F) Combined envelope and spectral power fit (r^2^_opt_) at each evaluated sedimentation rate. (G) Summary of 2000 Monte Carlo simulations with AR1 surrogates (ρ_AR1_ = 0.8065), used to evaluate the significance of the maximum observed r^2^_opt_ of 0.489 (p-value =0.0265). (H) Cross plot of the data amplitude envelope and the TimeOpt-reconstructed precession amplitude modulation model in panel “d”; dashed red line is the 1:1 line. Detailed settings and parameters for the Supplementary TimeOpt analysis of Subset D3 in the WD1 drillcore are provided in Supplementary Text 1.15.


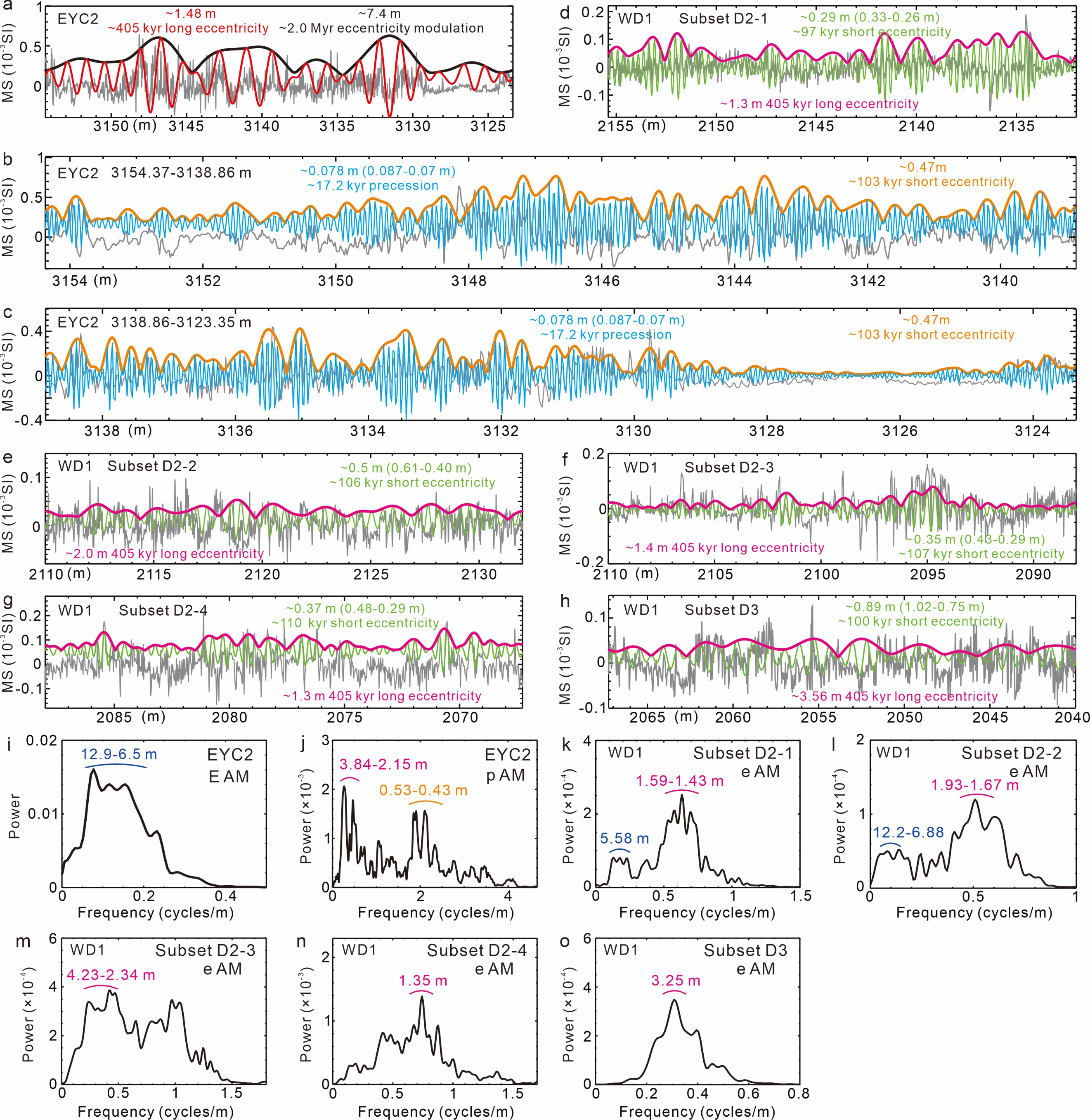


**Supplementary Fig. 25 Amplitude modulation (AM) envelopes from bandpass filters on EYC2 and WD1 drillcores, characterized via the Hilbert transform. (a-c)** Hilbert transform envelopes (~2.0 Myr eccentricity in black, ~103 kyr short eccentricity in orange) extracted using Tanner-Hilbert bandpass filters from the long eccentricity (red; passband: 0.47–0.88 cycles/m) and precession (blue; passband: 11.06–15.52 cycles/m) signals in the EYC2 drillcore. **(d-h)** Hilbert transform envelopes (~405 kyr eccentricity in bright red) extracted from short eccentricity (green) signals using filters with passbands for Subsets D2-1 (passband: 2.78–3.96 cycles/m), D2-2 (passband: 1.78–2.68 cycles/m), D2-3 (passband: 2.12–3.6 cycles/m), D2-4 (passband: 1.91–3.72 cycles/m), and D3 (passband: 0.84–1.44 cycles/m) in the WD1 drillcore. **(i–o)** MTM power spectra of the Hilbert transform envelopes from **a**–**h**, showing power in the 2.0 Myr eccentricity modulation and eccentricity bands (compared to Supplementary Figs. 7c, d and 9). Note: Hilbert transform envelopes (orange) from precession bandpass filters in **b** and **c** are shown separately for clarity, with **j** presenting their combined MTM power spectrum. AM amplitude modulation; E long eccentricity; e short eccentricity; P precession.


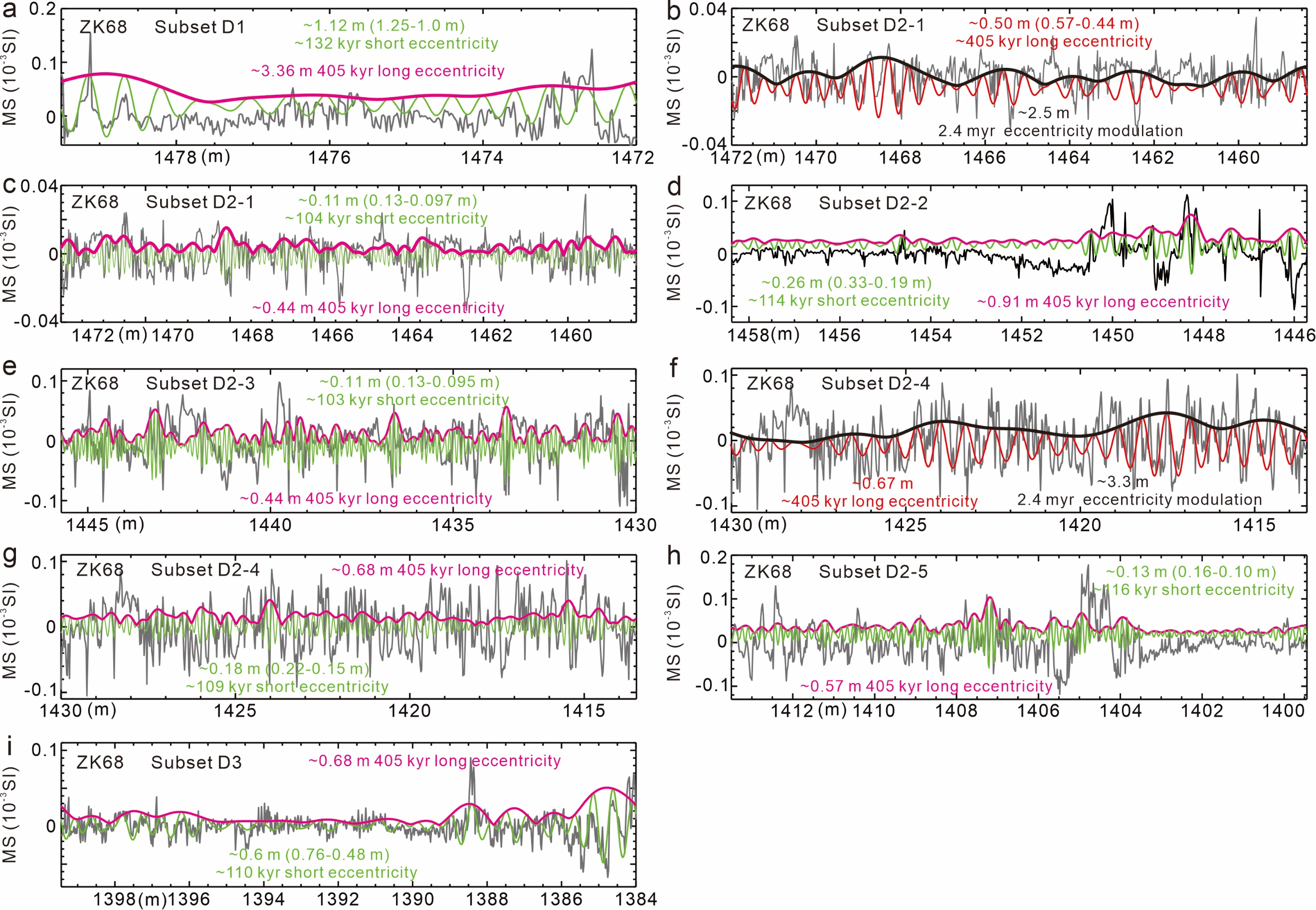


**Supplementary Fig. 26 Amplitude modulation (AM) envelopes from bandpass filters on ZK68 drillcores, characterized via the Hilbert transform. (a-i)** Hilbert transform envelopes (~2.4 Myr eccentricity modulation in black, ~405 kyr eccentricity in bright red) extracted using Tanner-Hilbert bandpass filters from long eccentricity (red) and short eccentricity (green) signals. Passbands used include Subsets D1 (0.7–1.3 cycles/m), D2-1 (1.61-2.49 cycles/m for long eccentricity amplitude modulation and 7.27-10.54 cycles/m for short eccentricity amplitude modulation), D2-2 (2.66-5.58 cycles/m), D2-3 (6.96-10.7 cycles/m), D2-4 (1.18-1.76 cycles/m for long eccentricity amplitude modulation and 4.26-7.06 cycles/m for short eccentricity amplitude modulation), D2-5 (6.05-9.47 cycles/m), and D3 (1.14–2.26 cycles/m) in the ZK68 drillcore.


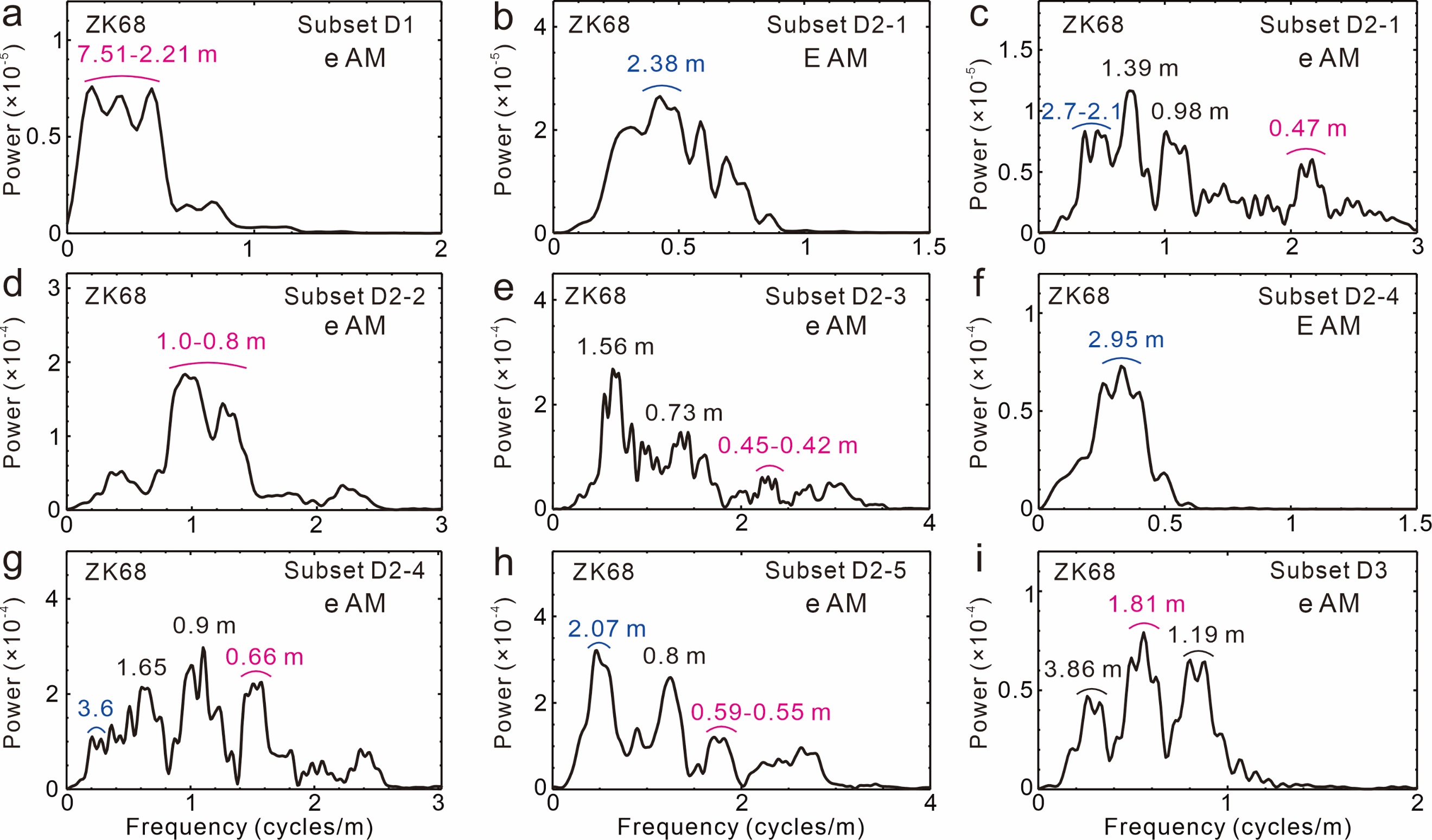


**Supplementary Fig. 27 MTM power spectra of the Hilbert transform envelopes from** **Supplementary Fig. 26 a-i**, showing power in the 2.4 Myr eccentricity modulation and eccentricity bands (compared to Supplementary Figs. 8c, d).


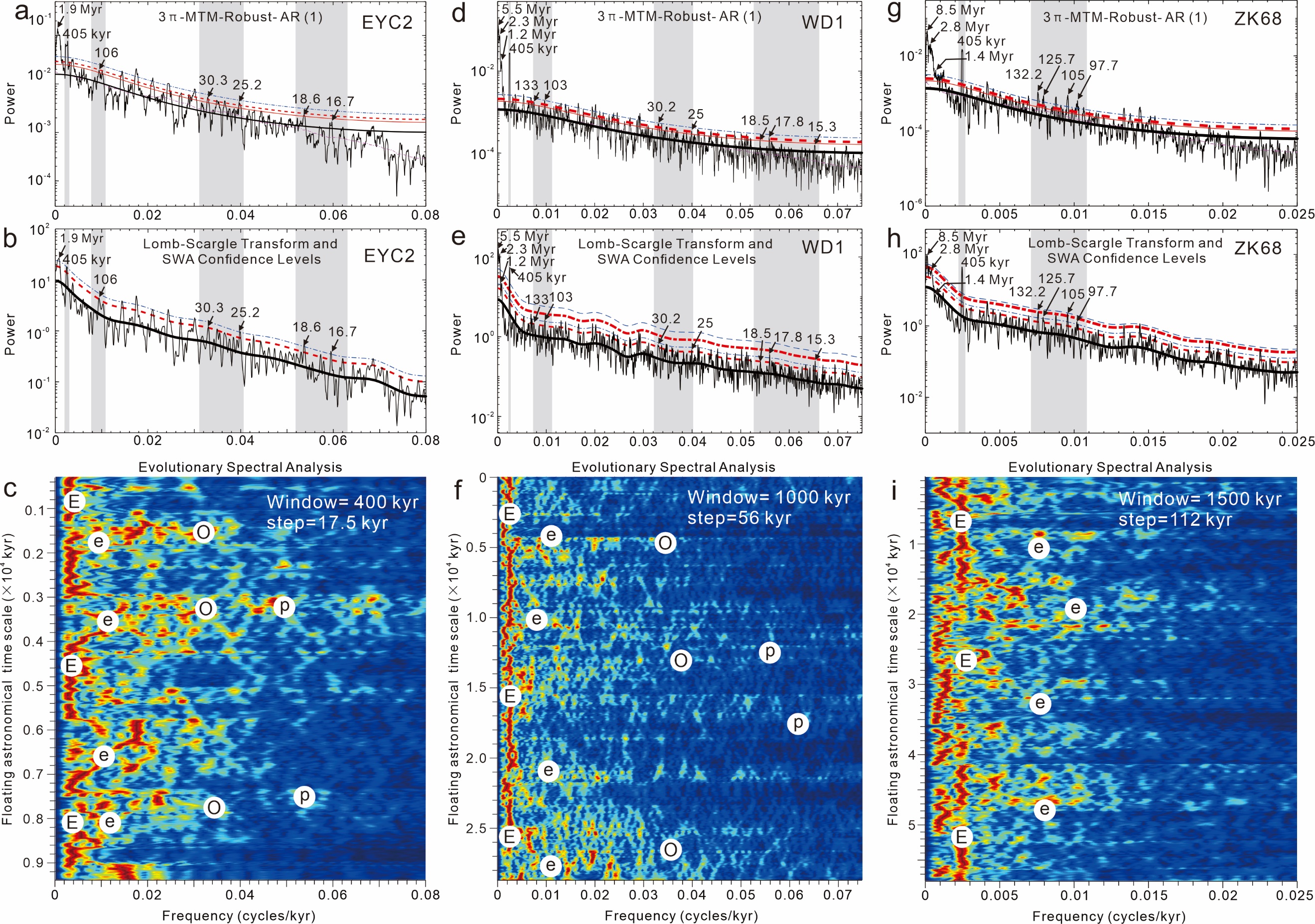


**Supplementary Fig. 28 Spectrum analyses of the tuned MS series of the EYC2, WD1 and ZK68 drillcores.** (**a, d, g**) 3π Multi-Taper Method (MTM) spectrum; (**b, e, g**) Lomb-Scargle periodogram using smoothed window averages (SWA) against the 5% false discovery rate (FDR); (**c f, i**). Evolutionary Fast Fourier Transform (eFFT) spectrum using 400 kyr, 1000 kyr and 1500 kyr sliding window with 17.5 kyr, 56 kyr, and 112 kyr steps for EYC2, WD1 and ZK68 drillcore, respectively. All analyses were applied to the tuned MS series after removing LOESS trends of 25% for EYC2, 35% for WD1 and 35% for ZK68.

**
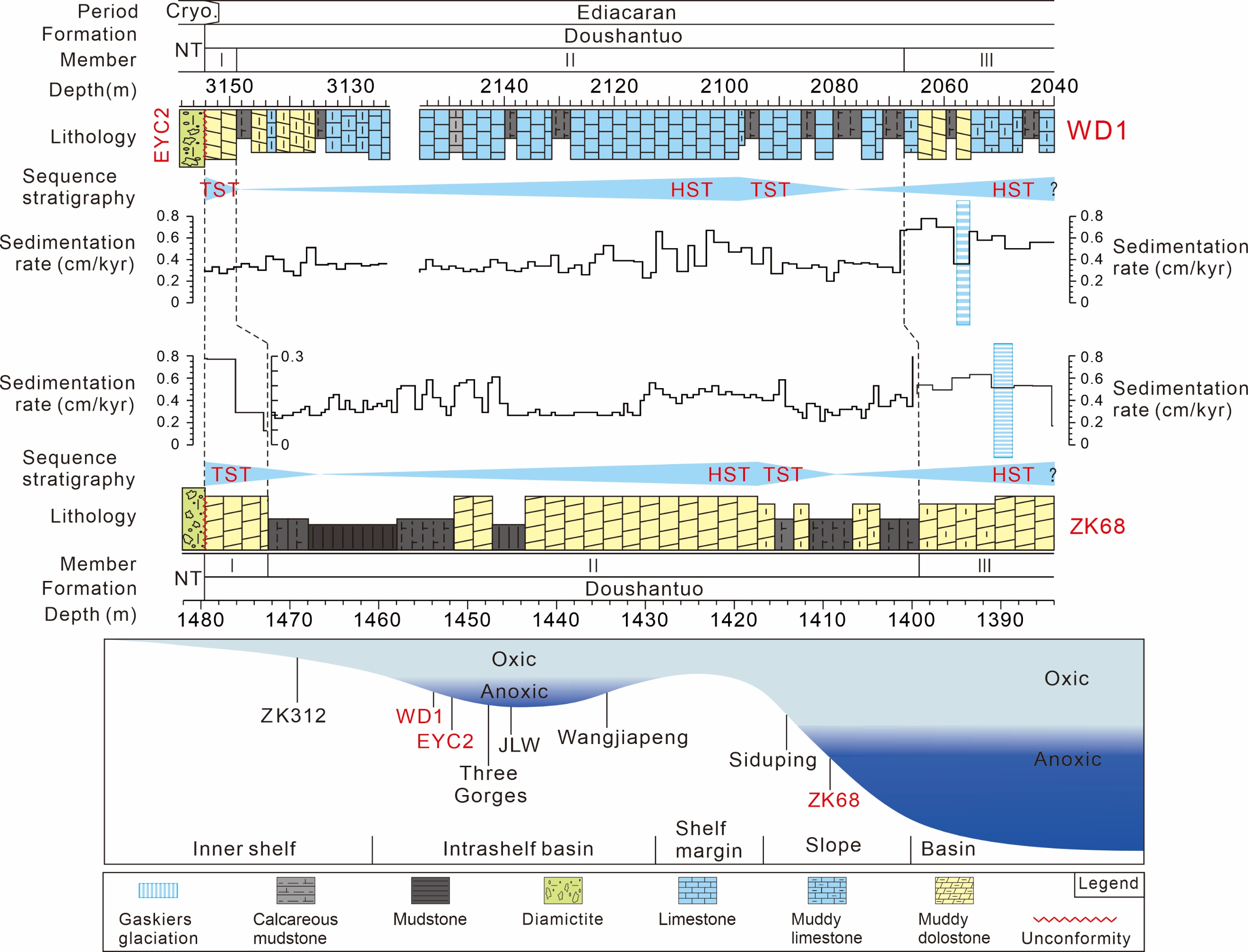
**

**Supplementary Figure 29 The integrated analysis of lithostratigraphy, sequence stratigraphy, and sedimentation rate variations.** The sedimentation rates are derived from the maxima of 405-kyr eccentricity cycles (black line, see Supplementary Data 2 for data). The sequence stratigraphy is based on ref. 12. TST Transgressive systems tract; HST Highstand systems tract.


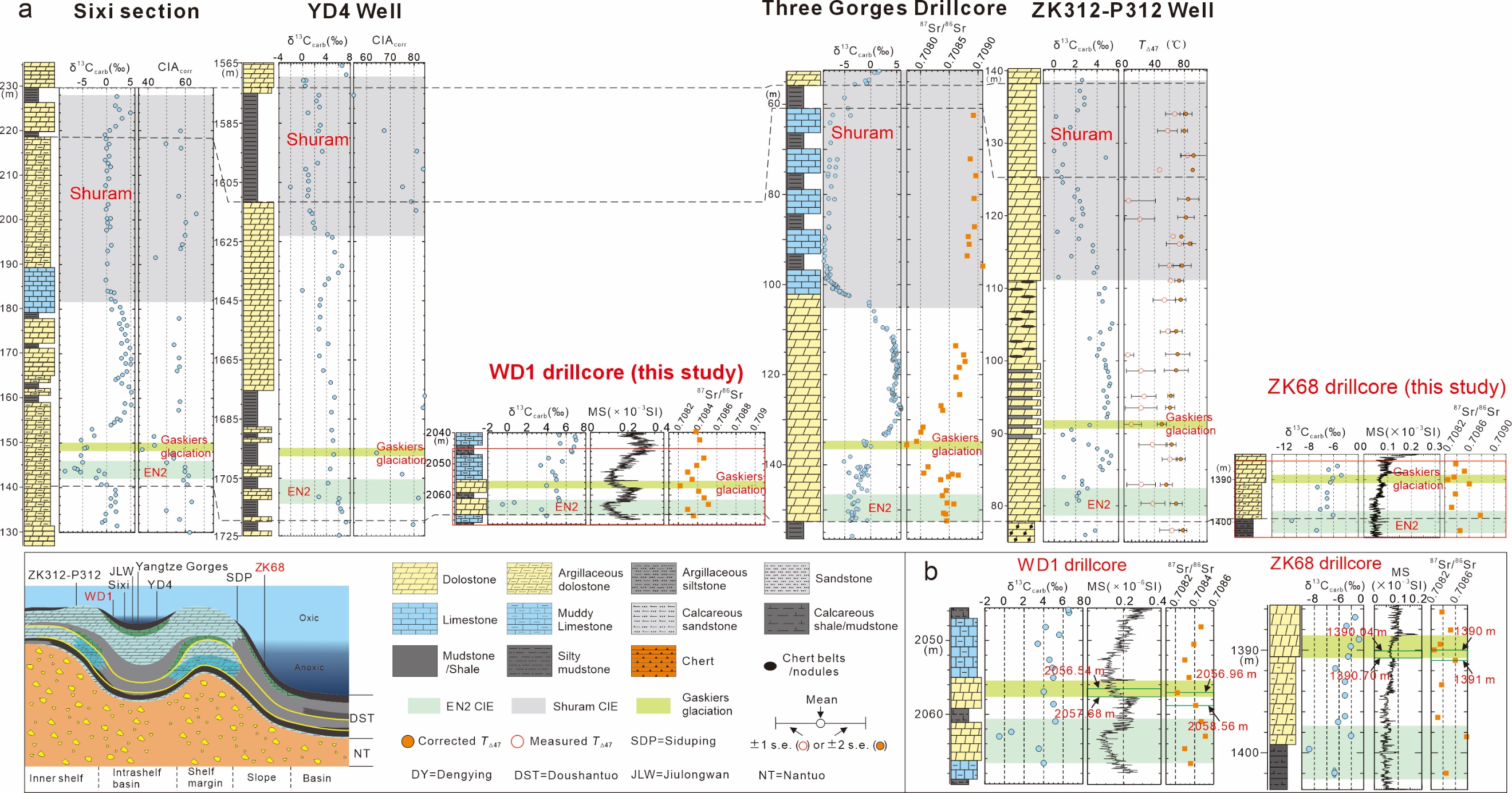


**Supplementary Fig. 30 The Gaskiers glaciation in South China. a** Multi-proxy paleoclimate correlation of the Doushantuo Formation Members III and IV in South China based on chemostratigraphy and lithostratigraphy: ^87^Sr/^86^Sr ratios, magnetic susceptibility (MS), Chemical Index of Alteration (CIA), and carbonate clumped-isotope temperatures (*T_Δ47_*). Data sources: Sixi section: δ^13^C_carb_^86^ and CIA^87^, YD4 Well: δ^13^C_carb_^70^ and CIA^87^, Three Gorges Drillcore: δ^13^C_carb_ and ^87^Sr/^86^Sr^25^, ZK312-P312 Well: δ^13^C_carb_ and *T*_Δ47_^23^. *T_Δ47_* represents the temperature of carbonate formation based on the carbonate clumped-isotope (Δ47) geothermometer (For more details on *T_Δ47_* please refer to ref. 23). The bottom left schematic diagram showing sedimentary facies variations of the Doushantuo Formation across a transect from north to south in the middle-upper part of the Yangtze Platform (modified from refs. 12,65). **b** Enlargement view of the red box area in **a.** The green box highlights a significant declining stage, defined by the transition from the initiation of significant decreases in MS and ^87^Sr/^86^Sr records to their glacial minima. Red numbers denote the corresponding stratigraphic depth range. CIA_corr_ indicates corrected chemical index of alteration (CIA) values for K-metasomatism.


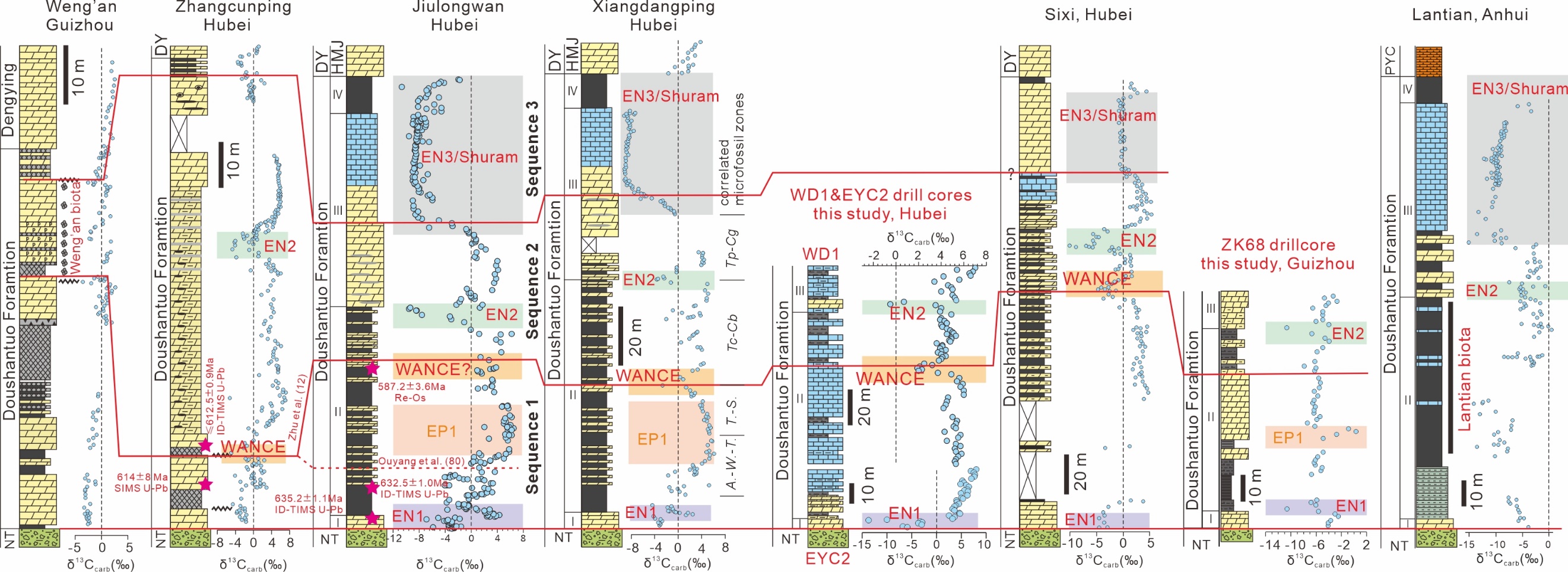


**Supplementary Fig. 31 Integrating sequence stratigraphy, radiometric dating, and fossil records from the Doushantuo Formation, South China.** Stratigraphic data sources: Weng’an^7^, Zhangcunping^62,69,80,88^, Jiulongwan^6,69^, WD1, ECY2 and ZK68 drillcores (this study), Xiang dangping^12,15,82^, Sixi^86^, and Lantian^72,89^. The solid red lines indicate the two sequence boundaries in the Doushantuo Formation proposed by Zhu et al.^12^, while the dashed red line represents the proposed correlation of the middle Doushantuo Member II carbon isotope excursion (CIE) between sections in the Weng'an and Yangtze Gorges areas, as suggested by Ouyang et al.^80^.

**Supplementary References**

1. Knauth, L. P. & Kennedy, M. J. The late Precambrian greening of the Earth. *Nature* **460**, 728–732 (2009).

2. Kaufman, A. J. & Knoll, A. H. Neoproterozoic variations in the C-isotopic composition of seawater: stratigraphic and biogeochemical implications. *Precambrian Res.* **73**, 27–49 (1995).

3. Banner, J. L. & Hanson, G. N. Calculation of simultaneous isotopic and trace element variations during water-rock interaction with applications to carbonate diagenesis. *Geochim. Cosmochim. Acta* **54**, 3123–3137 (1990).

4. Sreenivas, B. & Das Sharma, S. The Sr, C and O isotopic evolution of neoproterozoic seawater-comment. *Chem. Geol.* **181**, 193–195 (2001).

5. Ader, M. *et al.* A multilayered water column in the Ediacaran Yangtze platform? Insights from carbonate and organic matter paired δ13C. *Earth Planet. Sci. Lett.* **288**, 213–227 (2009).

6. McFadden, K. A. *et al.* Pulsed oxidation and biological evolution in the Ediacaran Doushantuo Formation. *Proc. Natl. Acad. Sci. U. S. A.* **105**, 3197–3202 (2008).

7. Zhou, C. & Xiao, S. Ediacaran δ13C chemostratigraphy of South China. *Chem. Geol.* **237**, 89–108 (2007).

8. Zhu, M., Strauss, H. & Shields, G. A. From snowball earth to the Cambrian bioradiation: Calibration of Ediacaran-Cambrian earth history in South China. *Palaeogeogr. Palaeoclimatol. Palaeoecol.* **254**, 1–6 (2007).

9. Lan, Z. *et al.* An integrated chemostratigraphic (δ13C-δ18O-87Sr/86Sr-δ15N) study of the Doushantuo Formation in western Hubei Province, South China. *Precambrian Res.* **320**, 232–252 (2019).

10. Wang, X., Jiang, G., Shi, X. & Xiao, S. Paired carbonate and organic carbon isotope variations of the Ediacaran Doushantuo Formation from an upper slope section at Siduping, South China. *Precambrian Res.* **273**, 53–66 (2016).

11. Xiao, S. *et al.* Towards an ediacaran time scale: Problems, protocols, and prospects. *Episodes* **39**, 540–555 (2016).

12. Zhu, M. *et al.* Carbon isotope chemostratigraphy and sedimentary facies evolution of the Ediacaran Doushantuo Formation in western Hubei, South China. *Precambrian Res.* **225**, 7–28 (2013).

13. Li, C. *et al.* Uncovering the spatial heterogeneity of Ediacaran carbon cycling. *Geobiology* **15**, 211–224 (2017).

14. Xiao, S. H. & Narbonne, G. M. *The Ediacaran Period*. *Geologic Time Scale 2020* (BV, 2020). doi:10.1016/B978-0-12-824360-2.00018-8.

15. An, Z. *et al.* Stratigraphic position of the Ediacaran Miaohe biota and its constrains on the age of the upper Doushantuo δ13C anomaly in the Yangtze Gorges area, South China. *Precambrian Res.* **271**, 243–253 (2015).

16. Calver, C. R. *et al.* Globally synchronous marinoan deglaciation indicated by U-Pb geochronology of the cottons Breccia, Tasmania, Australia. *Geology* **41**, 1127–1130 (2013).

17. Fike, D. A., Grotzinger, J. P., Pratt, L. M. & Summons, R. E. Oxidation of the Ediacaran ocean. *Nature* **444**, 744–747 (2006).

18. Moczydłowska, M. & Nagovitsin, K. E. Ediacaran radiation of organic-walled microbiota recorded in the Ura Formation, Patom Uplift, East Siberia. *Precambrian Res.* **198**–**199**, 1–24 (2012).

19. Zhu, M. *et al.* Cambrian integrative stratigraphy and timescale of China. *Sci. China Earth Sci.* **62**, 25–60 (2019).

20. Halverson, G. P., Hoffman, P. F., Schrag, D. P., Maloof, A. C. & Rice, A. H. N. Toward a Neoproterozoic composite carbon-isotope record. *Geol. Soc. Am. Bull.* **117**, 1181 (2005).

21. Canfield, D. E., Knoll, A. H., Poulton, S. W., Narbonne, G. M. & Dunning, G. R. Carbon isotopes in clastic rocks and the neoproterozoic carbon cycle. *Am. J. Sci.* **320**, 97–124 (2020).

22. Zhu, M., Zhang, J. & Yang, A. Integrated Ediacaran (Sinian) chronostratigraphy of South China. *Palaeogeogr. Palaeoclimatol. Palaeoecol.* **254**, 7–61 (2007).

23. Chen, B. *et al.* A short-lived oxidation event during the early Ediacaran and delayed oxygenation of the Proterozoic ocean. *Earth Planet. Sci. Lett.* **577**, 117274 (2022).

24. Gao, Y., Zhang, X., Zhang, G., Chen, K. & Shen, Y. Ediacaran negative C-isotopic excursions associated with phosphogenic events: Evidence from South China. *Precambrian Res.* **307**, 218–228 (2018).

25. Sawaki, Y. *et al.* The Ediacaran radiogenic Sr isotope excursion in the Doushantuo Formation in the Three Gorges area, South China. *Precambrian Res.* **176**, 46–64 (2010).

26. Fairchild, I. J. *et al.* Tonian-Cryogenian boundary sections of Argyll, Scotland. *Precambrian Res.* **319**, 37–64 (2018).

27. Walther, J. V. *Essentials of Geochemistry (READ)*. *Jones and Bartlett Publishers* (Jones & Bartlett Learning, 2005).

28. Brand, U., Azmy, K., Tazawa, J. I., Sano, H. & Buhl, D. Hydrothermal diagenesis of Paleozoic seamount carbonate components. *Chem. Geol.* **278**, 173–185 (2010).

29. Cui, H. *et al.* Primary or secondary? A dichotomy of the strontium isotope anomalies in the Ediacaran carbonates of Saudi Arabia. *Precambrian Res.* **343**, 105720 (2020).

30. Satkoski, A. M., Fralick, P., Beard, B. L. & Johnson, C. M. Initiation of modern-style plate tectonics recorded in Mesoarchean marine chemical sediments. *Geochim. Cosmochim. Acta* **209**, 216–232 (2017).

31. Cui, H. *et al.* Redox architecture of an Ediacaran ocean margin: Integrated chemostratigraphic (δ13C-δ34S-87Sr/86Sr-Ce/Ce*) correlation of the Doushantuo Formation, South China. *Chem. Geol.* **405**, 48–62 (2015).

32. Furuyama, S., Kano, A., Kunimitsu, Y., Ishikawa, T. & Wang, W. Diagenetic overprint to a negative carbon isotope anomaly associated with the Gaskiers glaciation of the Ediacaran Doushantuo Formation in South China. *Precambrian Res.* **276**, 110–122 (2016).

33. Wei, G. Y. *et al.* Long-term evolution of terrestrial inputs from the Ediacaran to early Cambrian: Clues from Nd isotopes in shallow-marine carbonates, South China. *Palaeogeogr. Palaeoclimatol. Palaeoecol.* **535**, 109367 (2019).

34. Broecker, W. S. & Peng, T. H. Tracers in the Sea, Lamont-Doherty Geological Observatory Press, Palisades, N. at (1982).

35. Chen, X., Zhou, Y. & Shields, G. A. Progress towards an improved Precambrian seawater 87Sr/86Sr curve. *Earth-Science Rev.* **224**, 103869 (2022).

36. Hodell, D. A., Mead, G. A. & Mueller, P. A. Variation in the strontium isotopic composition of seawater (8 Ma to present) : Implications for chemical weathering rates and dissolved fluxes to the oceans. *Chem. Geol. Isot. Geosci. Sect.* **80**, 291–307 (1990).

37. Albarède, F., Michard, A., Minster, J. F. & Michard, G. 87Sr86Sr ratios in hydrothermal waters and deposits from the East Pacific Rise at 21°N. *Earth Planet. Sci. Lett.* **55**, 229–236 (1981).

38. Richter, F. M., Rowley, D. B. & DePaolo, D. J. Sr isotope evolution of seawater: the role of tectonics. *Earth Planet. Sci. Lett.* **109**, 11–23 (1992).

39. Shields, G. A. A normalised seawater strontium isotope curve: possible implications for Neoproterozoic-Cambrian weathering rates and the further oxygenation of the Earth. *eEarth* **2**, 35–42 (2007).

40. Spooner, E. T. C. The strontium isotopic composition of seawater, and seawater-oceanic crust interaction. *Earth Planet. Sci. Lett.* **31**, 167–174 (1976).

41. Veizer, J., Hoefs, J., Lowe, D. R. & Thurston, P. C. Geochemistry of Precambrian carbonates: II. Archean greenstone belts and Archean sea water. *Geochim. Cosmochim. Acta* **53**, 859–871 (1989).

42. Palmer, M. R. & Edmond, J. M. The strontium isotope budget of the modern ocean. *Earth Planet. Sci. Lett.* **92**, 11–26 (1989).

43. Kirschvink, J. L., Ripperdan, R. L. & Evans, D. A. Evidence for a large-scale reorganization of Early Cambrian continental masses by inertial interchange true polar wander. *Science (80-. ).* **277**, 541–545 (1997).

44. Laskar, J. The chaotic motion of the solar system: A numerical estimate of the size of the chaotic zones. *Icarus* **88**, 266–291 (1990).

45. Laskar, J., Fienga, A., Gastineau, M. & Manche, H. La2010: A new orbital solution for the long-term motion of the Earth. *Astron. Astrophys.* **532**, A89 (2011).

46. Berger, A., Loutre, M. F. & Laskar, J. Stability of the Astronomical Frequencies Over the Earth’s History for Paleoclimate Studies. *Science (80-. ).* **255**, 560–566 (1992).

47. Berger, A. & Loutre, M. F. Astronomical Forcing through Geological Time. in *International Association of Sedimentologists Series* vol. 19 15–24 (Wiley, 2009).

48. Laskar, J. *et al.* A long-term numerical solution for the insolation quantities of the Earth. *Astron. Astrophys.* **428**, 261–285 (2004).

49. Waltham, D. Milankovitch period uncertainties and their impact on cyclostratigraphy. *J. Sediment. Res.* **85**, 990–998 (2015).

50. Zeebe, R. E. & Lantink, M. L. Milanković Forcing in Deep Time. *Paleoceanogr. Paleoclimatology* **39**, (2024).

51. Zeebe, R. E. & Lantink, M. L. A Secular Solar System Resonance that Disrupts the Dominant Cycle in Earth’s Orbital Eccentricity (g 2 − g 5 ): Implications for Astrochronology. *The Astronomical Journal* vol. 167 204 at https://doi.org/10.3847/1538-3881/ad32cf (2024).

52. Zeebe, R. E. & Kocken, I. J. Applying astronomical solutions and Milanković forcing in the Earth sciences. *Earth-Science Rev.* **261**, 104959 (2025).

53. Hinnov, L. A. Cyclostratigraphy and Astrochronology in 2018. in *The geologic time scale* 1–80 (Elsevier, 2018). doi:10.1016/bs.sats.2018.08.004.

54. Weedon, G. P., Page, K. N. & Jenkyns, H. C. Cyclostratigraphy, stratigraphic gaps and the duration of the Hettangian Stage (Jurassic): Insights from the Blue Lias Formation of southern Britain. *Geol. Mag.* **156**, 1469–1509 (2019).

55. Li, M. *et al.* Paleoclimate proxies for cyclostratigraphy: Comparative analysis using a Lower Triassic marine section in South China. *Earth-Science Rev.* **189**, 125–146 (2019).

56. Zhang, T. *et al.* Orbital forcing of tropical climate dynamics in the Early Cambrian. *Glob. Planet. Change* **219**, 103985 (2022).

57. Hoffman, P. F. & Li, Z. X. A palaeogeographic context for Neoproterozoic glaciation. *Palaeogeogr. Palaeoclimatol. Palaeoecol.* **277**, 158–172 (2009).

58. Li, Z. X., Evans, D. A. D. & Halverson, G. P. Neoproterozoic glaciations in a revised global palaeogeography from the breakup of Rodinia to the assembly of Gondwanaland. *Sediment. Geol.* **294**, 219–232 (2013).

59. McGee, B., Collins, A. S. & Trindade, R. I. F. A glacially incised canyon in Brazil: Further evidence for mid-ediacaran glaciation? *J. Geol.* **121**, 275–287 (2013).

60. EYLES, N. & EYLES, C. H. Glacially‐influenced deep‐marine sedimentation of the Late Precambrian Gaskiers Formation, Newfoundland, Canada. *Sedimentology* **36**, 601–620 (1989).

61. Myrow, P. M. & Kaufman, A. J. A newly discovered cap carbonate above varanger-age glacial deposits in newfoundland, CANADA. *J. Sediment. Res.* **69**, 784–793 (1999).

62. Wang, Z. *et al.* Silicified glendonites in the Ediacaran Doushantuo Formation (South China) and their potential paleoclimatic implications. *Geology* **45**, 115–118 (2017).

63. Chang, B. *et al.* A ∼60-Ma-long, high-resolution record of Ediacaran paleotemperature. *Sci. Bull.* **67**, 910–913 (2022).

64. Suess, E. *et al.* Calcium carbonate hexahydrate from organic-rich sediments of the antarctic shelf: Precursors of glendonites. *Science (80-. ).* **216**, 1128–1131 (1982).

65. Zhou, X. *et al.* Ikaite abundance controlled by porewater phosphorus level: Potential links to dust and productivity. *J. Geol.* **123**, 269–281 (2015).

66. Matthews, J. J. *et al.* A Chronostratigraphic Framework for the Rise of the Ediacaran Macrobiota: New Constraints from Mistaken Point Ecological Reserve, Newfoundland. *Bull. Geol. Soc. Am.* **133**, 612–624 (2021).

67. Pu, J. P. *et al.* Dodging snowballs: Geochronology of the Gaskiers glaciation and the first appearance of the Ediacaran biota. *Geology* **44**, 955–958 (2016).

68. Condon, D. *et al.* U-Pb ages from the neoproterozoic Doushantuo Formation, China. *Science (80-. ).* **308**, 95–98 (2005).

69. Yang, C. *et al.* The tempo of Ediacaran evolution. *Sci. Adv.* **7**, eabi9643 (2021).

70. Wang, Z. *et al.* Wide but not ubiquitous distribution of glendonite in the Doushantuo Formation, South China: Implications for Ediacaran climate. *Precambrian Res.* **338**, 105586 (2020).

71. Zhao, G. *et al.* Geological reconstructions of the East Asian blocks: From the breakup of Rodinia to the assembly of Pangea. *Earth-Science Rev.* **186**, 262–286 (2018).

72. Yuan, X., Chen, Z., Xiao, S., Zhou, C. & Hua, H. An early Ediacaran assemblage of macroscopic and morphologically differentiated eukaryotes. *Nature* **470**, 390–393 (2011).

73. Jiang, G., Shi, X., Zhang, S., Wang, Y. & Xiao, S. Stratigraphy and paleogeography of the Ediacaran Doushantuo Formation (ca. 635-551Ma) in South China. *Gondwana Res.* **19**, 831–849 (2011).

74. Xiao, S., Zhang, Y. & Knoll, A. H. Three-dimensional preservation of algae and animal embryos in a neoproterozoic phosphorite. *Nature* **391**, 553–558 (1998).

75. Xiao, S. *et al.* The Weng’an biota and the Ediacaran radiation of multicellular eukaryotes. *Natl. Sci. Rev.* **1**, 498–520 (2014).

76. Barfod, G. H. *et al.* New Lu-Hf and Pb-Pb age constraints on the earliest animal fossils. *Earth Planet. Sci. Lett.* **201**, 203–212 (2002).

77. Chen, D. F., Dong, W. Q., Zhu, B. Q. & Chen, X. P. Pb-Pb ages of Neoproterozoic Doushantuo phosphorites in South China: Constraints on early metazoan evolution and glaciation events. *Precambrian Res.* **132**, 123–132 (2004).

78. Chen, Y. Q., Jiang, S. Y., Ling, H. F. & Yang, J. H. Pb-Pb dating of black shales from the Lower Cambrian and Neoproterozoic strata, South China. *Chemie der Erde* **69**, 183–189 (2009).

79. Zhou, C. *et al.* A new SIMS zircon U-Pb date from the Ediacaran Doushantuo Formation: Age constraint on the Weng’an biota. *Geol. Mag.* **154**, 1193–1201 (2017).

80. Ouyang, Q., Zhou, C., Xiao, S., Chen, Z. & Shao, Y. Acanthomorphic acritarchs from the Ediacaran Doushantuo Formation at Zhangcunping in South China, with implications for the evolution of early Ediacaran eukaryotes. *Precambrian Res.* **320**, 171–192 (2019).

81. Sui, Y. *et al.* Astronomical time scale for the lower Doushantuo Formation of early Ediacaran, South China. *Sci. Bull.* **63**, 1485–1494 (2018).

82. Liu, P. & Moczydłowska, M. *Ediacaran microfossils from the doushantuo formation chert nodules in the yangtze gorges area, South China, and new biozones, Volume 65*. *Ediacaran Microfossils from the Doushantuo Formation Chert Nodules in the Yangtze Gorges Area, South China, and New Biozones* vol. 65 (John Wiley & Sons, Ltd, 2019).

83. Tahata, M. *et al.* Carbon and oxygen isotope chemostratigraphies of the Yangtze platform, South China: Decoding temperature and environmental changes through the Ediacaran. *Gondwana Res.* **23**, 333–353 (2013).

84. Kunimitsu, Y., Setsuda, Y., Furuyama, S., Wang, W. & Kano, A. Ediacaran chemostratigraphy and paleoceanography at a shallow marine setting in northwestern Hunan Province, South China. *Precambrian Res.* **191**, 194–208 (2011).

85. Meyers, S. R. The evaluation of eccentricity-related amplitude modulation and bundling in paleoclimate data: An inverse approach for astrochronologic testing and time scale optimization. *Paleoceanography* **30**, 1625–1640 (2015).

86. Chen, X. H., Zhou, P., Zhang, B. M. & Wang, C. S. Stable isotope records of the Ediacaran Doushantuo Formation in the Eastern Yangtze Gorges and its significance for choronostratigraphy. *Geol. China* **42**, 207–223 (2015).

87. Chen, C. *et al.* Variation of chemical index of alteration (CIA) in the Ediacaran Doushantuo Formation and its environmental implications. *Precambrian Res.* **347**, 105829 (2020).

88. Liu, P., Yin, C., Gao, L., Tang, F. & Chen, S. New material of microfossils from the Ediacaran Doushantuo Formation in the Zhangcunping area, Yichang, Hubei Province and its zircon SHRIMP U-Pb age. *Chinese Sci. Bull.* **54**, 1058–1064 (2009).

89. Cui, H. *et al.* An authigenic response to Ediacaran surface oxidation: Remarkable micron-scale isotopic heterogeneity revealed by SIMS. *Precambrian Res.* **377**, 106676 (2022).
