# Supplementary material for: Astronomically calibrating early Ediacaran evolution
Source: Nat Commun. 2025 Mar 28;16:3049. doi: 10.1038/s41467-025-57201-1 (PMC11953472; doi:10.1038/s41467-025-57201-1)
Supplement: Supplementary file 2 — Description of Additional Supplementary Files [file 41467_2025_57201_MOESM2_ESM.pdf]

Description of Additional Supplementary Files:

Supplementary Data 1: All isotopic results and geochemical data in this study

Supplementary Data 2: Magnetic susceptibility data, age models, floating astronomical time scale and sedimentation rate on 405 kyr scale

Supplementary Data 3: The predicted estimates of long eccentricity cycles from the astronomical solution ZB23-N64 at 610 Ma

Supplementary Data 4: A compilation of the Ediacaran strontium isotope ratios ($^{87}Sr/^{86}Sr$) and their age model from South China, Australia, and Namibia

Supplementary Data 5: A compilation of the Ediacaran $\delta^{13}C_{carb}$ data and their age model from Australia, South China, Laurientia, Mongolia, Namibia, Oman, and Siberia
